# Imidazolium‐Derived Porous Organic Polymer as Robust Platform for Rhodium‐Catalyzed N_2_O Hydrogenation and Alcohol Oxygenation

**DOI:** 10.1002/anie.202511917

**Published:** 2025-09-19

**Authors:** Sven Thomas Nappen, Veit Dippold, Darosch Asgari, Sarah Vogl, Hüseyin Küçükkeçeci, Arne Thomas, Monica Trincado, Hansjörg Grützmacher

**Affiliations:** ^1^ Department of Chemistry and Applied Biosciences ETH Zürich Vladimir‐Prelog‐Weg 1 Zürich CH‐8093 Switzerland; ^2^ Technische Universität Berlin Department of Chemistry/ Functional Materials Hardenbergstr. 40 10623 Berlin Germany; ^3^ Department of Chemistry University of Zürich Winterthurerstrasse 190 Zurich CH‐8057 Switzerland; ^4^ LIFM, IGCME School of Chemistry Sun Yat‐Sen University Guangzhou 510006 China

**Keywords:** Hydrogenation, Immobilization, Nitrous oxide, Porous organic polymers, Rhodium

## Abstract

The catalytic conversion of nitrous oxide, a potent greenhouse gas and ozone‐depleting substance, offers a promising strategy for mitigating emissions but requires efficient catalysts that operate under mild conditions. Here, a porous organic polymer was designed as a functional platform for accommodating built‐in catalytic sites. The polymer incorporates rigid 1,3‐dimethylbenzimidazolium iodide units as stable precursors to N‐heterocyclic carbenes, providing suitable coordination sites to molecular catalysts, high surface area, and chemical robustness. Deprotonating these precursors generates free carbene ligands that effectively coordinate and immobilize a rhodium bis(olefin) amine complex. Upon activation with base, the air stable immobilized complex forms reactive metal‐ligand cooperative Rh–N sites that convert nitrous oxide to nitrogen via in situ generated rhodium(I) hydride species. Exceptional performance was observed during catalytic hydrogenation of nitrous oxide under heterogeneous solid–liquid–gas conditions in batch reactors (using tetrahydrofuran or water) and under solid–gas conditions. Furthermore, the catalyst enabled the dehydrogenative coupling of primary alcohols (methanol, ethanol and benzyl alcohol) with nitrous oxide as a hydrogen acceptor, achieving turnover numbers that surpass all previously reported catalysts. These findings demonstrate the potential of porous organic polymer–metal complexes as robust, recyclable and efficient catalysts.

## Graphical abstract

A porous NHC‐polymer with large surface area anchors Rh amide sites to yield a stable and highly active catalyst for efficient hydrogenation of N_2_O and selective alcohol dehydrogenation, outperforming molecular analogues. Spatially arranged NHC sites prevent Rh deactivation and leaching, enabling sustainable conversion of a climate‐relevant gas into benign/valuable chemical products.

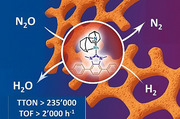

## Introduction

Nitrous oxide (N_2_O), commonly known as laughing gas, is not only a potent greenhouse gas, approximately 300 times more effective than CO_2_,^[^ ^1^ ^]^ but also currently the most ozone‐depleting substance in our stratosphere.^[^ ^2^ ^]^ Emissions continue to rise,^[^ ^3^, ^4^, ^5^ ^]^ largely driven by an increasing use of nitrogenous fertilizers^[^ ^6^ ^]^ and direct emissions from the chemical industry (ca. 10%). Coupled with its long atmospheric lifetime (approximately 115 years),^[^ ^7^ ^]^ the concentration of N_2_O in both the troposphere and stratosphere is expected to continue to increase unless effective mitigation strategies are implemented. Although N_2_O shows promise as a versatile and relatively little toxic^[^ ^8^, ^9^, ^10^ ^]^ reagent for oxygen‐ and nitrogen‐transfer reactions, dehydrogenations, and redox processes, its inertness limits broader utilization.^[^ ^11^, ^12^, ^13^ ^]^ Therefore, research on the catalytic depletion of N_2_O or its conversion into valuable chemicals is critical for mitigating emissions and unlocking its potential for chemical transformations.

Heterogeneous catalysts such as metal surfaces or zeolites can convert N_2_O to N_2_ under solid–gas conditions by decomposition or hydrogenation, but these processes currently require high temperatures (300–650 °C) and pressures.^[^ ^14^ ^]^ One of the most effective examples are iron‐containing acidic zeolites, which, at elevated temperatures, cleave N_2_O to form iron‐oxo species that can be used to oxygenate methane or benzene, yielding methanol or phenol, respectively.^[^ ^15^, ^16^, ^17^, ^18^ ^]^ Similarly, molybdenum on silica selectively oxidizes methanol to formaldehyde using N_2_O.^[^ ^19^ ^]^ Although partial oxidation of methanol with N_2_O has been studied,^[^ ^20^, ^21^, ^22^, ^23^, ^24^ ^]^ complete oxidation to CO_2_ remains largely unexplored—which is surprising given the interest in methanol as a potential fuel.^[^ ^25^, ^26^ ^]^ Methanol/N_2_O mixtures offer the potential to be a potent and relatively safe fuel, provided the energy from the reaction: 3 N_2_O + CH_3_OH → 3 N_2_ + CO_2_ + 2 H_2_O (Δ*H*ᵣ = −220.4 kcal·mol^−1^) can be effectively harnessed.

**Figure 1:**
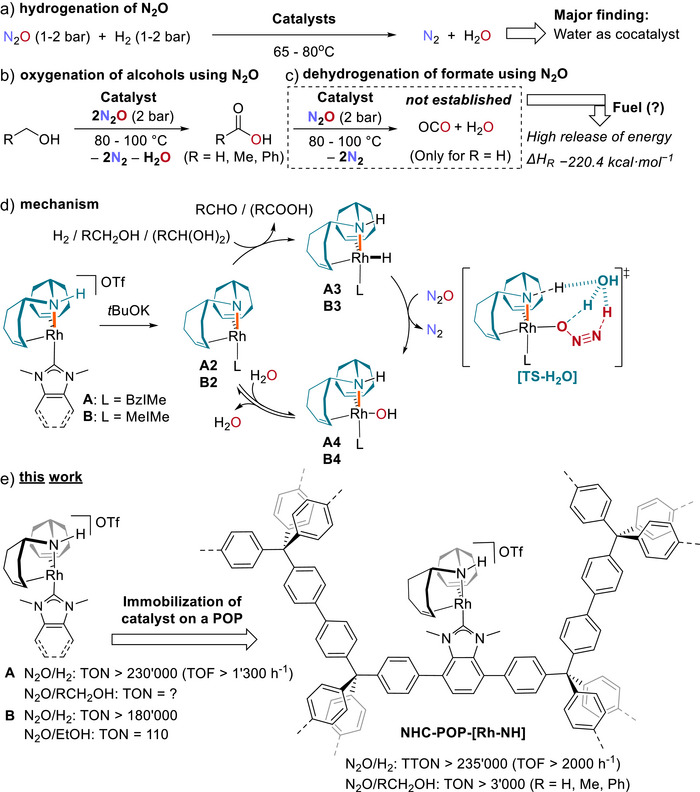
The development of a heterogeneous catalyst based on a molecular bis(trop)amine NHC rhodium complex. a) the hydrogenation reaction of nitrous oxide; b) the dehydrogenation of alcohols to carboxylic acids using N_2_O as the hydrogen acceptor; c) the unestablished oxidation of methanol to CO_2_ using N_2_O as the oxidant; d) simplified mechanism involving metal‐ligand cooperativity with the reactive Rh─N bond (highlighted in orange) in complexes **A‐A4**,**B‐B4**. e) This work shows the immobilization of the molecular complex on a NHC‐based porous organic polymer conforming a more efficient catalyst.

Homogeneous catalysts that activate N_2_O at mild temperatures in solution are equally intriguing and provide valuable mechanistic insights into the efficient transformation of N_2_O. (Figure 1d).^[^ ^27^, ^28^, ^29^, ^30^, ^31^ ^]^ Recently, a rhodium(I) olefin‐amine complex, [Rh(trop_2_NH)(BzIMe)]OTf (**A**) [trop_2_NH = bis(5H‐dibenzo[a,d]cycloheptene‐5‐yl)amine, BzIMe = 1,3‐dimethylbenzimidazol‐2‐ylidene], was discovered to be highly active for converting N_2_O/H_2_ mixtures to N_2_ and H_2_O, achieving turnover numbers (TONs) of 230′000 and turnover frequencies (TOFs) of 1′300 h^−1^ in tetrahydrofuran (THF), with water acting as a co‐catalyst (Figure 1a).^[^ ^32^ ^]^ A related NHC catalyst [Rh(trop_2_NH)(MeIMe)]OTf (**B**) (MeIMe = 1,2,3,4‐tetramethylimidazolium carbene) also promotes the dehydrogenative coupling of alcohols to carboxylic acids (or esters) using N_2_O as a hydrogen acceptor (Figure 1b).^[^ ^33^ ^]^ This would be a highly interesting reaction especially when the oxidation of light alcohols (e.g., methanol, ethanol) is targeted, which are produced annually at million ton scales.^[^ ^27^, ^31^ ^]^ Since catalysts **A** and **B** are insoluble in water, organic solvents must be used for their operation, and their solid‐state crystal packing limits their broader application in materials technologies. Heterogenization of the complex presents a compelling approach to obtain an efficient catalyst that can operate under solid–gas conditions, eliminating the need for solvents (Figure 1e).

The molecular catalysts mentioned above use metal‐ligand cooperativity to activate H_2_ and ultimately reduce N_2_O to N_2_,^[^ ^34^, ^35^, ^36^, ^37^, ^38^, ^39^, ^40^ ^]^ a mechanistic principle that is also found in nature. The enzyme N_2_O reductase catalyzes the conversion of N_2_O to N_2_ and H_2_O under mild conditions, using binuclear and tetranuclear copper complexes to activate N_2_O, along with a lysine unit that shuttles protons to the active site.^[^ ^41^, ^42^, ^43^ ^]^ Although replicating the enzyme's active site has proven to be difficult owing to its structural complexity, insights into its cooperative catalytic mechanism have inspired the development of laboratory‐designed synthetic molecular compounds.^[^ ^27^, ^28^, ^29^, ^30^, ^31^ ^]^

Immobilizing molecular catalysts in porous solids offers a possibility to yield heterogeneous catalysts featuring well‐defined active sites and local environments that can function efficiently under mild conditions. The combination of structural complexity with straightforward characterization might help to obtain better insight into the reaction mechanism of heterogeneous catalysts. Carbon–carbon bonded porous organic polymers (POPs) are especially attractive for the heterogenization of homogeneous metal–organic catalysts as they provide tunable and robust covalently linked networks.^[^ ^44^, ^45^, ^46^ ^]^ Among various synthetic approaches, Yamamoto coupling (YC) enables (co)polymerization of halide‐functionalized monomers, enabling access to polymers with high structural diversity and high porosity.^[^ ^47^, ^48^ ^]^ Incorporating N‐heterocyclic carbenes (NHCs) into POPs can yield hybrid catalysts with well‐defined active sites.^[^ ^49^, ^50^ ^]^ To date, NHCs are typically incorporated in the POPs via a long carbon side‐chain or via the flexible and bent N–R group on the imidazole unit, leading to NHC‐POPs with reduced surface areas and thereby limiting their broader catalytic potential.^[^ ^51^, ^52^, ^53^, ^54^ ^]^ To overcome this, we designed a benzimidazole‐based precursor with rigid, linear connectivity that enables the synthesis of a highly porous NHC‐POP. Copolymerization with tetrakis(4‐bromophenyl)methane via YC, followed by post synthetic modifications, allowed the rhodium bis(trop) amine complex (**3**) to be immobilized within a hydrophobic pore environment, generating a heterogenized version of **A**. Remarkably, the resulting catalyst outperforms its homogeneous analogue in N_2_O hydrogenation, successfully operating in water and even under solid–gas conditions.

## Results and discussion

### Synthesis and Characterization

**Scheme 1:**
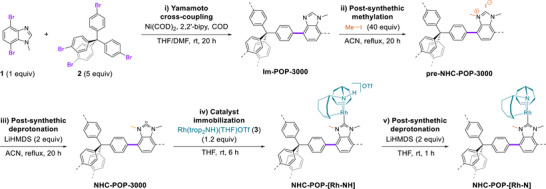
Synthesis and activation of the NHC‐POP‐immobilized rhodium olefin‐amine catalyst. i) Yamamoto cross‐coupling polymerization of monomers **1** and **2** to yield **Im‐POP‐3000**. ii) Post‐synthetic methylation to form **preNHC‐POP‐3000**. iii) Post‐synthetic deprotonation to produce **NHC‐POP‐3000**. iv) Immobilization of complex **3** resulting in **NHC‐POP‐[Rh‐NH]**. v) Activation of the catalyst through post‐synthetic deprotonation to yield the active catalyst **NHC‐POP‐[Rh‐N]**.

**Figure 2:**
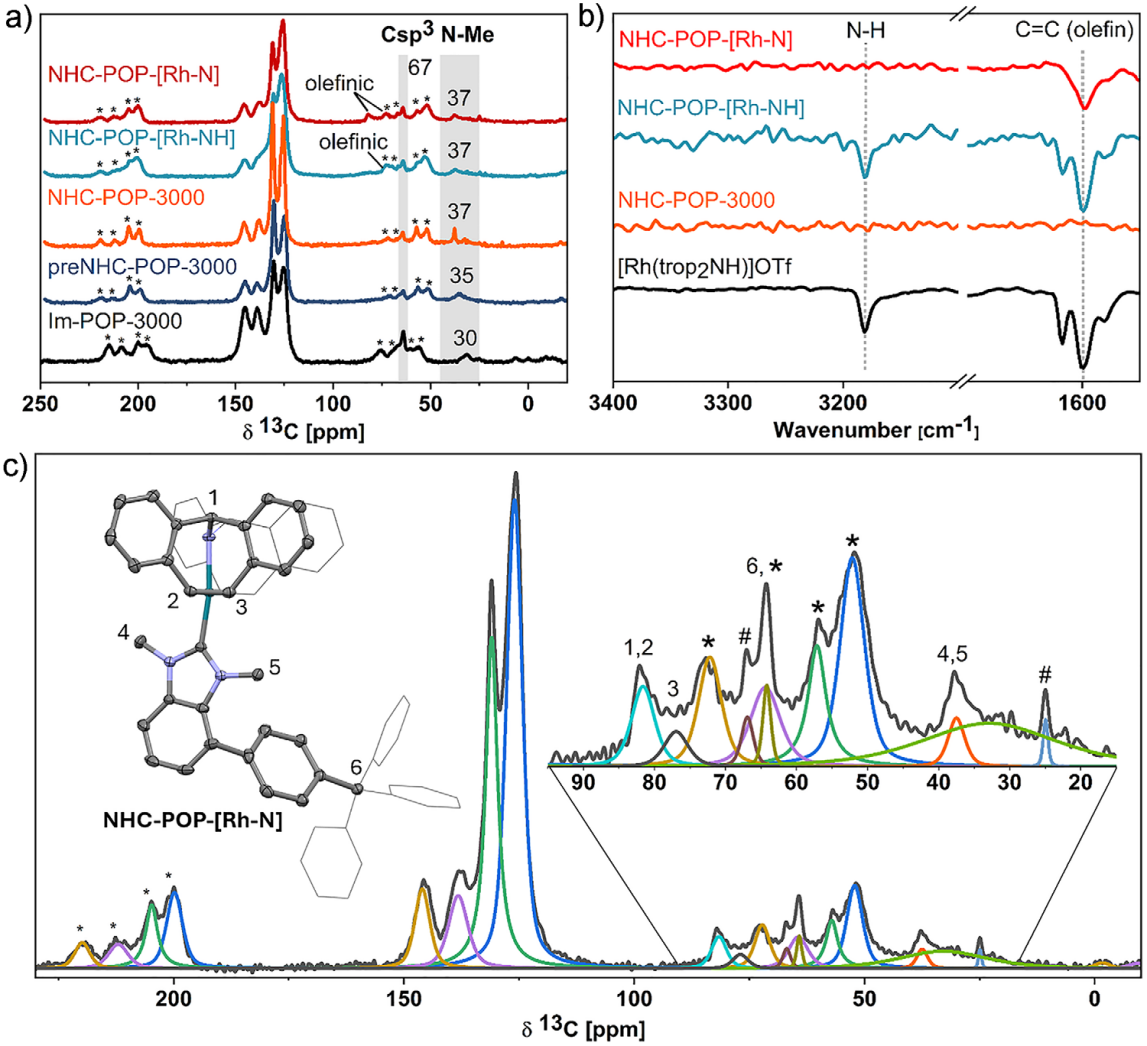
Selected ^13^C CP/MAS NMR and FT‐IR spectroscopy spectra of the POP before and after catalyst immobilization and activation. a) stacked ^13^C CP/MAS NMR spectra of **Im‐POP‐3000** (black), **preNHC‐POP‐3000** (dark blue), **NHC‐POP‐3000** (orange), **NHC‐POP‐[Rh‐NH]** (light blue), and **NHC‐POP‐[Rh‐N]** (red). b) stacked FT‐IR spectra of complex **3** (black), **NHC‐POP‐3000** (yellow), **NHC‐POP‐[Rh‐NH]** (green), and **NHC‐POP‐[Rh‐N]** (red). c) deconvoluted ^13^C CP/MAS NMR spectrum of **NHC‐POP‐[Rh‐N]** with assigned peaks (1–6). Spinning‐sidebands are marked with (*) and THF with (#). The **NHC‐POP‐[Rh‐NH]** unit in the figure is custom made from the SC‐XRD structures of [Rh(trop_2_N)(BzIMe)] (**A2**) and tetraphenylmethane (TPM).

The complete synthesis of the activated immobilized catalyst proceeds through a five‐step procedure, of which the last four steps are post synthetic modifications (Scheme 1, i). The molecular building blocks and rhodium complexes were synthetized and analyzed as described in the supplementary information (see Figures S1–S21). First, a porous polymer (**Im‐POP‐3000)** was prepared by copolymerizing the monomer 4,7‐dibromo‐1‐methylbenzimidazole (**1**) with tetrakis(4‐bromophenyl)methane (**2**) in a 1:5 ratio via YC reaction (Scheme 1). The solid state ^13^C cross‐polarized (CP) magic angle spinning (MAS) nuclear magnetic resonance (NMR) spectrum of **Im‐POP‐3000** displays a distinct resonance at 67 ppm, corresponding to the sp^3^‐C nucleus in the tetraphenylmethane units, and resonances between 146 and 125 ppm, assigned to the aromatic carbon nuclei of the phenyl rings (Figures 2a and S23). The N–CH_3_ methyl nucleus of the 1‐methylbenzimidazole unit resonates at 30 ppm, confirming successful copolymerization. In the second step, the 1‐methylbenzimidazole NH units were methylated with methyl iodide, yielding the yellow‐colored **preNHC‐POP‐3000**. A downfield shift of the N–CH_3_^13^C resonance from 30 to 35 ppm confirms the formation of 1,3‐dimethylbenzimidazolium units (Scheme 1, ii).

**Figure 3:**
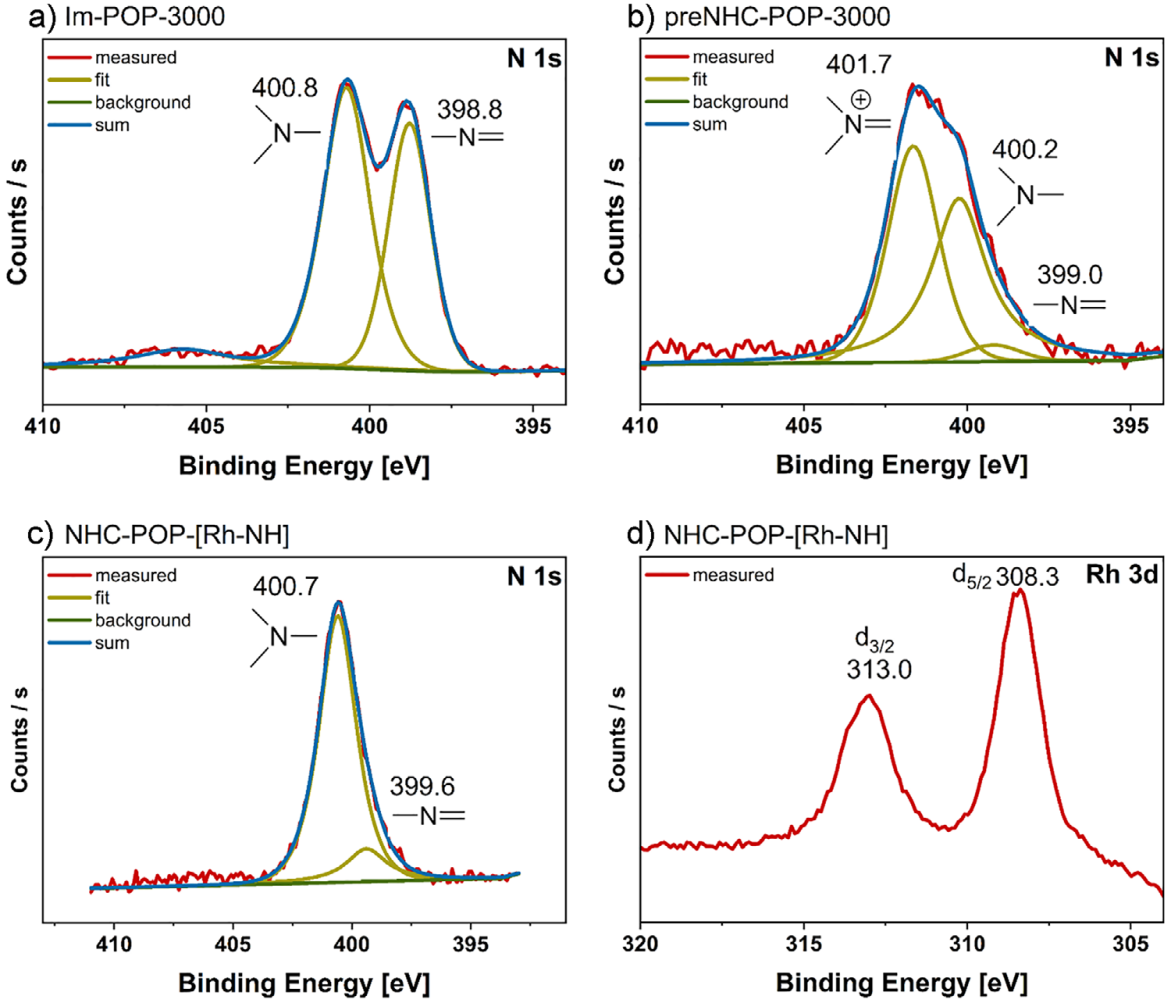
Selected XPS spectra of the POP before and after catalyst immobilization. a)–c) N 1s spectrum of **Im‐POP‐3000**, **preNHC‐POP‐3000,** and **NHC‐POP‐[Rh‐NH]**, respectively. d) 3d Rh spectrum of **NHC‐POP‐[Rh‐N]**.

X‐ray photoelectron spectroscopy (XPS) analyses further confirm the modifications (Figure 3). In the deconvoluted nitrogen 1s core‐level spectrum of **Im‐POP‐3000**, two signals in a 1:1 ratio at 400.8 and 398.8 eV are attributed to the tertiary amine and imine nitrogen atoms of the 1‐methylbenzimidazole units, respectively (Figure 3a and S25). Upon methylation to **preNHC‐POP‐3000** the signal at 398.8 eV diminishes, and a new signal at 401.7 eV emerges, corresponding to the positively charged iminium nitrogen atom on the 1,3‐dimethylimidazolium iodide units (Figure 3b). Integration of the residual signal at 399.0 eV suggests a high efficiency of the methylation step (93%). As expected, the iodine 3d XPS spectrum shows a doublet at 618.7 eV (3d_5/2_) and 630.3 eV (3d_3/2_) confirming the presence of iodide counterions (Figure S26).

**Figure 4:**
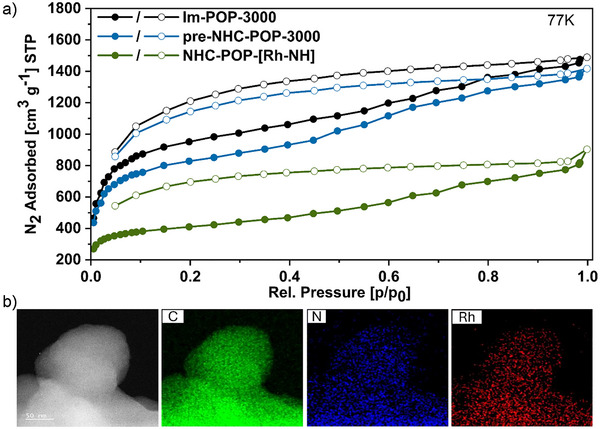
a) BET‐plots of the POP before and after catalyst immobilization. N_2_ sorption isotherms at 77 K of **Im‐POP‐3000** (black), **preNHC‐POP‐3000** (blue), and **NHC‐POP‐[Rh‐NH]** (green). b) EDX images of **NHC‐POP‐[Rh‐N]** and mapping distributions of carbon (green), nitrogen (blue), and rhodium (red) elements.

The permanent porosity of the polymers was assessed by low‐temperature nitrogen sorption measurements, which showed a type I sorption isotherm with steep uptake at very low relative pressures (<0.2 p/p_0_), characteristic for microporous materials (Figure 4a). Using Brunauer–Emett–Teller (BET) theory, **Im‐POP‐3000** exhibited a surface area of 3608 m^2^ g^−1^ and a pore size distribution centered around 1.73 nm (Figure S22a). Methylation to **preNHC‐POP‐3000** reduced the surface area, due to partial pore occupation by methyl groups and iodide anions, resulting in a still high BET surface area of 3110 m^2^ g^−1^ with a pore size distribution centered around 1.70 nm (Figure S22b). Both isotherms display a pronounced hysteresis as often observed for this material class due to delayed desorption.^[^ ^55^, ^56^ ^]^ Thermogravimetric analysis (TGA) revealed excellent thermal stability of **Im‐POP‐3000** and **preNHC‐POP‐3000** with no weight loss below 500 °C under air and nitrogen atmosphere (Figure S36).

### Catalyst Immobilization

Deprotonation of **preNHC‐POP‐3000** with bis(trimethylsilyl)amide (LiHMDS) afforded a pink‐colored, air‐sensitive **NHC‐POP‐3000** containing free NHC sites (Scheme 1, iii). Fourier‐transform infrared (FT‐IR) spectroscopy confirmed the deprotonation of the benzimidazolium units by the loss of the N_2_C–H absorption, and ^13^C CP/MAS NMR revealed a downfield shift of the N–CH_3_ resonance from 35 to 37 ppm (Figures 2a,b and S37). Coordination of the cationic rhodium complex Rh(trop_2_NH)(THF)OTf (**1**) to the newly formed free carbene sites yielded the orange‐colored, air‐stable material **NHC‐POP‐[Rh‐NH]** (Scheme 1, iv). In the ^13^C CP/MAS NMR spectrum, a new broad resonance at 73 ppm is assigned to overlapping olefinic and benzylic carbons of the immobilized rhodium complex, and the N–CH_3_ resonance broadened, likely as the binding to rhodium results in two magnetically inequivalent N–CH_3_ nuclei, pointing either toward or away from the active Rh‐N site (Figure 2a). In the ^19^F MAS NMR spectrum, a strong signal at 79 ppm corresponds to the triflate counterion (Figure S24). FT‐IR spectroscopy revealed new N–H and C = C absorptions at 3180 and 1599 cm^−1^, respectively (Figures 2b and S37). XPS analysis corroborated these findings, with the N 1s spectrum showing a peak at 400.7 eV attributed to the bis‑tropamine and NHC nitrogens, and a signal at 399.1 eV assigned to residual imidazole nitrogen (Figure 3c and S27). The Rh 3d core‐level spectrum exhibited a doublet at 308.3 eV (3d_5/2_) and 313.0 eV (3d_3/2_), consistent with rhodium(I), while the F 1s spectrum displayed a peak at 688.4 eV indicative of the triflate anion (Figure 3d and S28). The XPS analysis of the polymer matches closely that of the molecular complex **A** (Figure S29). Even after immobilization and the molecular catalyst residing within the pores, **NHC‐POP‐[Rh‐NH]** still possessed a BET surface area of 1519 m^2^ g^−1^ (Figure 4a). No other metal contamination was detected by ICP‐OES and SEM‐EDX with a Rh loading between 2.4–3.4 wt% over multiple synthetic batches (Figure S39 and S43–S45). High‐angle annular dark‐field scanning transmission electron microscopy (HAADF‐STEM) and TEM‐EDX analysis of the activated catalyst **NHC‐POP‐[Rh‐N]** (vide infra) also showed that the complex was uniformly distributed on the POP and the absence of nanoparticles (Figure 4b and S47 and S48). Provided that all methylbenzimidazole was embedded within the polymer, this corresponds to an immobilization efficiency of 55–77%. Overall, the analyses are consistent with the successful immobilization of [Rh(trop_2_NH)]OTf complexes by coordination to the NHC sites within the polymer (see Figure 1).

### Catalyst Activation

The relatively acidic NH protons of the trop_2_NH ligand in **NHC‐POP‐[Rh‐NH]** were deprotonated with LiHMDS to yield the green, air‐sensitive material **NHC‐POP‐[Rh‐N]** containing polarized and reactive Rh─N bonds (Scheme 1).^[^ ^57^, ^58^, ^59^ ^]^ This transformation was confirmed with ^19^F MAS NMR spectroscopy by the absence of the resonance signal attributed to the triflate counterion in the NMR spectrum (Figure S24) and with FT‐IR spectroscopy by the disappearance of the N─H stretching frequency (Figures 2b and S37). XPS analyses showed no significant changes in the Rh or N species (Figure S30), while ICP‐OES showed that the rhodium loading remained at 2.4 wt%, matching the Rh content of the pre‐catalyst **NHC‐POP‐[Rh‐NH]** and ruling out significant leaching (Figure S27 and S44). The color shift corresponds to that of the homogeneous amido complex [Rh(trop_2_N)(BzIMe)] (**A2**). In contrast to the amido complex in solution, the immobilized complex displays reduced dynamics in the solid‐state at room temperature by which inversion of the [Rh(trop_2_N)] unit occurs along the Rh─N bond.^[^ ^59^, ^60^ ^]^ Indeed, the deconvoluted ^13^C CP/MAS NMR spectrum of **NHC‐POP‐[Rh‐N]** shows high frequency‐shifted benzylic and olefinic carbons at 82 and 69 ppm, respectively (Figure 2c). The signals are assumed to arise from partial occupation of the pores by THF which weakly coordinates to the vacant Rh coordination sites in the pores. Such interactions may also account for the observed catalytic activity (vide infra) by allowing water molecules to enter the pores through association and dissociation at the Rh–N sites and thereby enabling water‐mediated proton shuttling crucial for efficient catalysis.

## Catalysis

### N_2_O Hydrogenation

**Figure 5:**
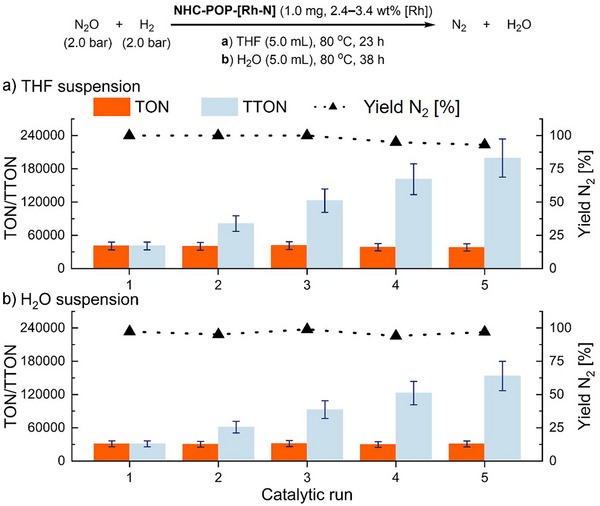
Reusability of **NHC‐POP‐[Rh‐N]** catalyst in N_2_O hydrogenation under different solvent conditions. a) catalytic runs in diluted THF suspensions. b) catalytic runs in diluted, nonsuspended pure water mixtures. **Experimental conditions**: Catalyst **NHC‐POP‐[Rh‐N]** (1.0 mg, 0.05–0.07 mM [Rh]), N_2_O (2.0 bar absolute, 11.8 mmol [w/solvent]), H_2_ (2.0 bar absolute, 10.9 mmol), and solvent (THF or H_2_O, 5.0 mL). After 23 h a) or 38 h b), the reactor was depressurized, subjected to three freeze‐pump‐thaw cycles, and repressurized with reagents to initiate a new run. TONs and TOFs were determined via GC‐TCD analysis of N_2_ (± 7.5% standard deviation) and, for (a), verified by ^1^H‐NMR quantification of H_2_O (±2.0% error) using mesitylene (20.0 µL) as an internal standard. Detection of H_2_ by GC‐TCD served as a control (See Supporting Information). The activity range of the catalyst is given as a dependency on the range of catalyst loading 2.4–3.4 wt% over the synthetic batches (±17% standard deviation).

The immobilized rhodium complex was tested for the catalytic hydrogenation of nitrous oxide (N_2_O), resulting in the formation of nitrogen (N_2_) and water (H_2_O) (Figure 5). The reactions were carried out under heterogeneous solid–liquid–gas conditions in batch reactors. The catalyst **NHC‐POP‐[Rh‐N]** (1.0 mg, 20–30 ppm [Rh]) was tested in highly diluted suspensions of THF and H_2_O (5.0 mL, 0.05–0.07 mM [Rh]), enabling direct comparison with the homogeneous molecular catalyst Rh(trop_2_N)(BzIMe) (**A2**) (see Table S4).^[^ ^32^ ^]^ Reactions were conducted under mild conditions with N_2_O (2.0 bar, 11.8 mmol w/solvent) and H_2_ (2.0 bar, 10.9 mmol) at 80 °C. Nitrogen formation was measured using GC‐TCD analysis; in THF suspensions, water formation was also quantified. To assess the reusability of the catalyst, five catalytic runs were performed where the reactor was depressurized after 23 h for THF suspensions and 38 h when water was used as solvent. After each run, the reactor was repressurized with N_2_O and H_2_ and restarted under the same conditions. These results were reproduced in parallel experiments, confirming the reproducibility of the procedure.

Across multiple synthetic batches, the activated and immobilized catalyst **NHC‐POP‐[Rh‐N]** displayed exceptionally high activity in THF suspensions, achieving a TON of 40′900 (±7′050) and TOF of 1′780 (±306) h^−1^ (Figure 5). Its robustness was evident by reusability tests: **NHC‐POP‐[Rh‐N]** retained its high activity over five consecutive runs, with N_2_ yields decreasing only slightly from 100% to 93%, resulting in a total TON (TTON) of 199′300 (±34′360). Notably, the immobilized catalyst also offered a key advantage over its homogeneous counterpart (**A**) when water was used as reaction medium due to its hydrophobic nature. Whereas the molecular catalyst **A** forms a gel‐like precipitate at the bottom of the reactor and fails to form a reactive suspension, **NHC‐POP‐[Rh‐N]** remains active in water by floating at the water–gas interface. Under these conditions, it reached a TON of 30′950 (±5′330) and a TOF of 815 (±140) h^−1^ during the first run, accumulating a total TON of 153′450 (±26′460) over five cycles. N_2_O is less soluble in water than in THF leading to less N_2_O available for conversion partly explaining the slightly lower performance in water. Nevertheless, the retention of high conversion rates (95–99%) over successive runs indicates the catalyst's high stability in an aqueous medium.

To examine the post‐catalysis state of **NHC‐POP‐[Rh‐N]**, the experiment above was repeated with 50 times more catalyst (50.0 mg, 6′600–9′380 ppm [Rh]) to have enough material for analysis. Control experiments showed that the filtered catalyst retained only about one‐third of its original activity under high‐loading conditions (Table S5, Figure S52). The filtrate is catalytically inactive, confirming that any leached rhodium species do not contribute to catalysis. XPS analyses of **NHC‐POP‐[Rh‐N]** after catalysis revealed no significant changes in the NHC nitrogen signals (400 eV, 399.5 eV) or the Rh 3d doublets (308.5 eV, 313.2 eV), matching the data of the unused catalyst (Figure S31–S32). Notably, no additional signals were detected in the Rh 3d region pointing toward a deactivated rhodium complex. In comparison to that, partial catalyst deactivation was observed for the homogeneous analogue in which the Rh–N active site has inserted into a C─H bond (Figure S14–S17, S33, and S49, and Table S4). Minimal leaching of the Rh‐complex from the NHC binding sites on the polymer were shown by ICP‐OES (Figure S43–S46). Furthermore, SEM‐EDX analysis revealed no changes in the polymer morphology or in the rhodium distribution (Figure S41). It may be that at higher concentrations residual triflate anions in the pores react with H_2_O and the rhodium sites, thereby reforming the pre‐catalyst (as previously observed in the molecular system).^[^ ^32^ ^]^

**Figure 6:**
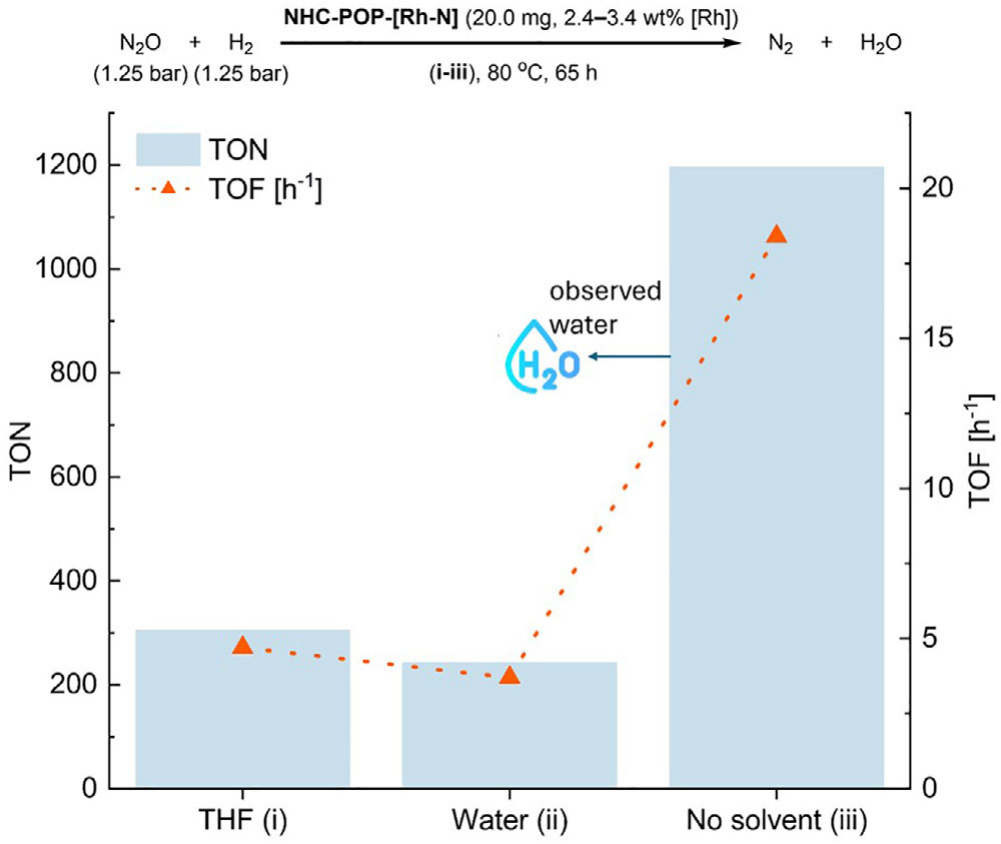
Comparison of **NHC‐POP‐[Rh‐N]** activity under solvent and nonsolvent conditions. i) catalytic run in THF (5.0 mL) suspension. ii) catalytic run in water (5.0 mL). iii) Catalytic run in solid–gas mode (no added solvent). Note: During the solid–gas experiment, water condensation was observed on the reactor walls. See Supporting Information for experimental details.

The catalyst performance under heterogeneous solid–gas conditions was compared with those using a solvent. Three new experiments were set up using a higher catalyst loading to enable post‐catalysis analysis (Figure 6). The catalyst was pre‐impregnated with water to provide humidity and facilitate initiation. The water‐impregnated **NHC‐POP‐[Rh‐N]** (20.0 mg, 430–600 ppm [Rh]) was placed at the bottom of a batch reactor, pressurized with N_2_O and H_2_ (1.25 bar, 6.8 mmol each), and heated to 80 °C. Under these conditions, the catalyst achieved 96% conversion after 65 h, with a TON of 1′195 (±205) and TOF of 18 (±3) h^−1^. Notably, when the catalyst is tested under these conditions the performance exceeded that in concentrated suspensions (20 mg/5 mL) by approximately a factor of 4 (THF) or 5 (water). The absence of further Rh 3d signals in the XPS spectrum of the used catalyst than the one of the precatalyst attests minimal deactivation of the active sites also under solid–gas conditions (Figure S34–S35). Minor leaching is observed, and the rhodium sites are retained to 93% after catalysis based on ICP‐OES of the post‐catalysis state (Figures S43–S46). Additionally, no change of the polymer morphology was observed during catalysis (Figure S38).

### Dehydrogenation of Primary Alcohols Using N_2_O as Hydrogen Acceptor

N_2_O and H_2_ form explosive mixtures and a safer catalytic system for N_2_O hydrogenation is desirable. This could consist in the conversion of nitrous oxide and methanol mixtures to N_2_ according to: 3·N_2_O + H_3_COH → 3·N_2_ + CO_2_ + 2·H_2_O (Δ*H*_R_ = −220.4 kcal·mol^−1^), which also would be an interesting fuel.^[^ ^19^, ^20^, ^21^, ^22^, ^23^, ^24^, ^25^, ^26^ ^]^ In line with this idea, in situ prepared **NHC‐POP‐[Rh‐N]** was tested in the oxygenation of primary alcohols (RCH_2_OH, R = Ph, Me, H) to carboxylates (RCOO^−^) in presence of base using N_2_O as the hydrogen acceptor generating N_2_ and water as the only byproducts according to: RCH_2_OH + 2·N_2_O + base → (baseH)^+^ + RCOO^−^ + 2·N_2_ + H_2_O) (Table 1). The catalyst (1.7 mg, 300 ppm) was combined with alcohol (1.0 mL) and *tert*‐butoxide *t*BuOM (1.6 mmol; M = Na or K) in THF (5.0 mL), then pressurized with N_2_O (3.0 bar, 17.8 mmol) and heated at 100 °C for 115 h. Under these conditions, benzyl alcohol was almost fully converted to benzoate (94%), while methanol and ethanol showed moderate conversions to formate (65%) and acetate (33%) respectively. Despite these moderate efficiencies, the catalyst achieved benchmark TONs of 2′160 (± 370) for methanol and 1′080 (± 185) for ethanol, surpassing previous reported TONs using different catalytic systems (up to 210).^[^ ^31^ ^]^

**Table 1:** Oxygenation of primary alcohols using N_2_O.

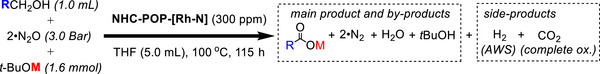								
Entry	R	M	[RC(O)O]M [P] (mmol)	N_2_ (mmol)	H_2_ (mmol)	CO_2_ (detected)	Yield_[P]_ (%)	TON_P_ (TON_N2_)
**1**	**Ph**	**K**	1.51	2.14	0.84	–	94	3140 (4450)
**2**	**H**	**Na**	1.04	3.16	0.47	^a)^	65	2160 (6580)
**3**	**Me**	**Na**	0.52	0.72	0.27	–	33	1080 (1500)
**4** ^b)^	**Ph**	**K**	0.36	0.85	0	–	23	750 (1770)
**5** ^c)^	**H**	–	0.16^d)^	0.62	0.25	^e)^	10	23 (89)

^a)^ CO_2_ was detected, approximately 12 times more concentrated than in ambient air (∼ 4800 ppm).

^b)^ THF/H_2_O (2.5/2.5 mL) was used as solvent mixture.

^c)^ Reaction conditions: Catalyst **B** (5.0 mg, 0.007 mmol, 140 ppm), no base, no solvent, MeOH (0.45 mL, neat), 48 h; H_2_O was detected as a product (0.42 mmol).

^d)^ The main product is formic acid (not formate).

^e)^ CO_2_ was detected, approximately 8 times more concentrated than in ambient air. Determination of yields: The carboxylate product yield is expressed as a percentage relative to the limiting reagent. For entries 1–4, the base is the limiting reagent; for entry 5, MeOH is considered the limiting reagent (the yield would be 37.5% if calculated using a hypothetical base amount). The carboxylate product was quantified by NMR analysis of the crude reaction mixture with Na_3_‐citrate as an internal standard, and the gas‐phase products were measured and quantified via GC‐TCD analysis of the reaction.

Reaction monitoring revealed that carboxylate formation proceeds via a classical two‐step oxidation through the aldehyde as intermediate, which is in equilibrium with the gem‐diol in presence of water. The latter is subsequently dehydrogenated to the corresponding carboxylate. In total, two equivalents of N_2_O per carboxylate product are consumed (supporting previous findings).^[^ ^31^, ^33^ ^]^ In benzyl alcohol oxidation, water can act as an alternative oxidant converting benzaldehyde to benzoate via an aldehyde–water shift (AWS) reaction that liberates H_2_ as byproduct (Table 1, entry 1: benzoate = 1.51 mmol, N_2_ = 2.14 mmol, H_2_ = 0.84 mmol). Contrary to Le‐Chatelier's principle, the AWS reaction was suppressed when using a H_2_O:THF (1:1) solvent mixture (Table 1, entry 4), suggesting that the reaction is likely under kinetic control via a hydroxide (or related) complex as intermediate (Figure 1d). In methanol oxidation, the excess of N_2_ (3.16 mmol) relative to formate (1.04 mmol) indicated further oxidation to CO_2_, which was indeed confirmed by gas‐phase analysis showing an approximate 12‐fold higher CO_2_ concentration than is usually present in air. This reaction was further probed by the molecular complex (**B**), which when heated in neat methanol (no base or extra solvent) produced CO_2_ and formic acid but in small amounts only. Subjecting the amide complex **B2** to a N_2_O/CO_2_/H_2_ mixture yielded a stable rhodium O‐bound formate complex (**B‐HCO_2_**), confirmed by single crystal X‐ray diffraction (Figure S50), via CO_2_ insertion into the in situ generated Rh─H bond. The formation of this complex and its stability makes the active Rh‐N site inaccessible for N_2_O as reagent, explaining the limited methanol‐to‐CO_2_ conversion.

## Conclusion

A porous organic polymer bearing a N‐heterocycle was successfully synthesized by Yamamoto cross‐coupling consisting of 1‐methylbenzimidazole and tetraphenylmethane building blocks. Subsequent conversion to 1,3‐dimethylbenzimidazol‐2‐ylidene enabled the efficient immobilization of a cationic olefin–amine rhodium complex. This material was activated resulting in the immobilized amide complex **NHC‐POP‐[Rh‐N]** without detectable metal‐leaching. As catalyst for N_2_O hydrogenation it demonstrated exceptional activity and stability under diverse conditions including diluted THF suspensions, water–gas interfaces, and solid–gas phases, surpassing its molecular analogue (TTON of up to 235′000 and TOF > 2′000h^−1^). The purposeful structural design, combining spatially separated NHC coordination sites with strong electron‐donating characteristics, not only prevents Rh aggregation and dimerization‐induced deactivation (previously postulated for molecular catalysts)^[^ ^32^ ^]^ but also firmly anchors the Rh–amine active species, effectively minimizing metal leaching and allowing reutilization.

The dehydrogenative coupling of primary alcohols, achieving benchmark turnover numbers (TONs) of 2160 (± 370) for the N_2_O‐driven methanol‐to‐formate conversion, represents a significant advance over the previous state of the art. These preliminary results also indicate that a conversion of methanol‐to‐CO_2_ may be established. The results reported here show that the polymeric material **NHC‐POP‐[Rh‐NH]**, designed and constructed from a molecular Rh(I) amine precursor, can be activated in the same manner as its molecular Rh(I) amine NHC catalysts, yielding immobilized Rh(I) amide sites that promote N_2_O/H_2_ conversion likely on the same minimum energy reaction pathway (MERP).^[^ ^32^ ^]^ These findings underscore the utility of N_2_O as an oxidant for alcohol dehydrogenation reactions and show a possible way of harnessing the energy of methanol/NO*_x_* mixtures (best in form of ammonium nitrate as precursor to N_2_O and methanol as a potential fuel). Metal amido bonds are promising as cooperative catalytically active sites but must be stabilized against protonation or insertion into C─H bonds while metal hydrides on the MERP should not be transformed into stable carbonates. This may be achieved by continuous‐flow systems to mitigate CO_2_ and acid buildup. Designing a catalyst with selectivity over the two isoelectronic and isostructural N_2_O/CO_2_ molecules still stand as a grand challenge, which when resolved could yield materials with great potential as catalysts, sensors or gas‐separation applications.

## Author Contributions

S.T.N., V.D.: Conceptualization, Data Curation, Visualization, Investigation, Writing—original draft, Writing—review & editing, D.A.: Conceptualization, Investigation, Writing—original draft, Writing—review & editing, S.V.: Conceptualization, Data curation, Investigation, H.K.: Investigation, A.T.: Conceptualization, Writing—review & editing, Project Administration, Funding Acquisition, Supervision, M.T.: Conceptualization, Funding Acquisition, Supervision, Review & editing, H.G.: Conceptualization, Writing—review & editing, Project Administration, Funding Acquisition, Supervision.

## Conflict of Interests

The authors declare no conflict of interest.

## Supporting information



Supporting Information

## Data Availability

The data that support the findings of this study are available in the Supporting Information of this article.
