# Supplementary material for: Imidazolium‐Derived Porous Organic Polymer as Robust Platform for Rhodium‐Catalyzed N2O Hydrogenation and Alcohol Oxygenation
Source: Angew Chem Int Ed Engl. 2025 Sep 19;64(46):e202511917. doi: 10.1002/anie.202511917 (PMC12603983; doi:10.1002/anie.202511917)
Supplement: Supplementary file 1 — Supporting Information [file ANIE-64-e202511917-s001.pdf]

Supplementary Information

Imidazolium-Derived Porous Organic Polymer as Robust Platform for Rhodium-Catalyzed N_2O Hydrogenation and Alcohol Oxygenation

Sven Thomas Nappen,[1+] Veit Dippold,[2+] Darosch Asgari,[2+] Sarah Vogl,[2] Hüseyin Küçükkeçeci,[2] Arne Thomas,[2] Monica Trincado,[1,3] and Hansjörg Grützmacher[1,4]

[1] Department of Chemistry and Applied Biosciences, ETH Zürich, Vladimir-Prelog-Weg 1, CH-8093 Zürich, Switzerland

[2] Technische Universität Berlin, Department of Chemistry/ Functional Materials, Hardenbergstr. 40, 10623 Berlin, Germany

[3] Department of Chemistry, University of Zürich, Winterthurerstrasse 190, CH-8057 Zurich, Switzerland

[4] LIFM, IGCME, School of Chemistry, Sun Yat-Sen University, 510006 Guangzhou, China

List of Contents

1. Supplementary notes of general techniques 4
   1.1 Handling of chemicals and chemical equipment 4
   1.2 Thermogravimetric analysis (TGA) 4
   1.3 Physisorption measurements 4
   1.4 Scanning electron microscopy (SEM) with energy dispersive X-ray (EDX) analysis 4
   1.5 Nuclear magnetic resonance (NMR) spectroscopy 4
   1.6 Fourier-transform infrared (FT-IR) spectroscopy 5
   1.7 X-ray photoelectron spectroscopy (XPS) 5
   1.8 Single crystal X-ray diffraction (SC-XRD) measurements 5
   1.9 Gas chromatography (GC) thermal conductivity detector (GC-TCD) analysis 5
   1.10 High-angle annular dark-field scanning TEM (HAADF-STEM) and energy-dispersive X-ray analysis 5
2. Supplementary notes of syntheses 6
   2.1 Synthesis of complexes and ligands 6
   2.2 Synthesis of monomers 10
   2.3 Synthesis of copolymer 10
   2.4 Immobilization of complex on polymer 11
   2.5 Activation of catalyst 12
3. Supplementary figures of liquid NMR spectra 13
4. Supplementary tables of liquid NMR data 23
5. Supplementary figures of physisorption measurements 24
6. Supplementary figures of solid-state NMR spectra 24
7. Supplementary figures of XPS spectra 26
8. Supplementary figures of TGA spectra 32
9. Supplementary figures of FT-IR spectra 33
10. SEM-EDX Analysis 34
11. Supplementary figures of ICP-OES measurements 39
12. Supplementary figures of HAADF-STEM EDX Analysis 42
13. Supplementary notes of crystallographic data 43
    13.1 Supplementary notes of crystal structure information 43
    13.2 Supplementary tables and figures of structural data 43
14. Supplementary notes of catalytic investigations 47
    14.1 Supplementary notes of the reaction setup 47

14.2 Supplementary notes of the of the general procedure of the N_2O hydrogenation reaction in suspension 48

14.3 Supplementary notes of the general procedure of the N_2O hydrogenation reaction in heterogeneous solid-gas phase 48

14.4 Supplementary notes of the general procedure for alcohol dehydrogenation reaction using N_2O as hydrogen acceptor 49

14.5 Supplementary tables and figures of catalysis screening 50

14.6 Supplementary notes of H_2O quantification using ^1H NMR-spectroscopy 54

14.7 Supplementary notes of the N_2 quantification using GC-TCD 57

14.8 Supplementary notes of the H_2 quantification using GC-TCD 67

15. Supplementary notes of the catalyst robustness 69

16. References 71

1. Supplementary notes of general techniques

1.1 Handling of chemicals and chemical equipment

Glassware was pre-cleaned with appropriate solvents and washed in a dishwasher employing neodisher LaboClean A8 and deionized water. Unless otherwise noted, all reactions were carried out in oven-dried (120 °C) glassware. Fritted glass funnels (POR 4) used for filtration were cleaned with piranha-solution (H_2SO_4:H_2O_2 = 3:1) and flushed with deionized water. Magnetic stir bars were cleaned, depending on the amount and type of impurities, either with acetone and deionized water or in a H_2SO_4-bath and rinsed with deionized water. Reactions requiring inert conditions were performed under argon atmosphere using standard Schlenk and vacuum-line techniques or in an MBraun glove box (Argon). Glassware was flame dried on a Schlenk line or kept at 120 °C overnight prior to use. Solvents were dried and stored over 4 Å molecular sieves under Ar. Deuterated solvents were purchased from Eurisotop, degassed, distilled from the proper drying agent, and stored over 4 Å molecular sieves under argon. The argon was provided by PanGas and further purified with an MBraun >99 HP gas purification system. Other gases (N_2O and H_2) were received from PanGas. Nitrous oxide (5.0) and hydrogen (5.0) was used as received. 3,6-dibromo-1,2-benzenediamine and tetrakis(4-bromophenyl)methane was purchased from BLDpharm (97%). Triethyl orthoformiate, cyclooctadiene and methyl iodide were purchased from Sigma-Aldrich. Bis(cycloocta-1,5-diene)nickel was purchased from ABCR. Anhydrous solvents were purchased from Acros Chemicals. 2,2'-Bipyridine was purchased from TCI.

1.2 Thermogravimetric analysis (TGA)

TGA was performed on a Mettler Toledo TGA 1 Stare thermal analyzer under nitrogen or air flow in a temperature interval of 30–800 °C with a heating rate of 5 °C per minute.

1.3 Physisorption measurements

Low pressure nitrogen physisorption isotherms were measured volumetrically at 77 K up to a pressure of 1 bar using a Quadrasorb SI (Quantachrome) equipped with CryoCooler (Quadrasorb) for temperature regulation. Measurement data was analyzed using "ASiQwin 4.01 Build 13" (Quantachrome). BET surface areas were calculated using isotherm values which were chosen by the three consistency criteria according to Rouquerol. The pore size distributions were calculated from the adsorption isotherms by Quenched Solid State Functional Theory (QSDFT) using N_2 sorption data collected at 77 K.

1.4 Scanning electron microscopy (SEM) with energy dispersive X-ray (EDX) analysis

Scanning electron microscopy (SEM) was performed at the "Zentraleinrichtung Elektronenmikroskopie" at the Technical University of Berlin. SEM images were taken on a GeminiSEM 500 NanoVP (ZEISS). Materials were dispersed on a silicon wafer and adhered to a flat aluminum platform sample holder. Energy Dispersive Spectroscopy (EDX) measurements were recorded with the Bruker EDX-System Quantax XFlash 6|60. Due to the low amount of some elements they were seen everywhere in the EDX image.

1.5 Nuclear magnetic resonance (NMR) spectroscopy

^{13}C Solid-state cross-polarization (CP) magic-angle spinning (MAS) nuclear magnetic resonance (NMR) and ^{19}F MAS NMR were recorded on a Bruker 700 MHz spectrometer using double resonance 3.2mm CP-MAS probe. Samples were packed in 3.2mm zirconia rotors inside an Ar-filled glovebox, and all

spectra were recorded at 298 K. In all cases, the downfield ^{13}C resonance of adamantane (38.5 ppm) was used as an external secondary reference to calibrate chemical shifts. T1(^1H) was measured using a saturation recovery experiment. For all experiments, N_2 was used to provide both bearing and drive pressure. The MAS frequency was set to 13kHz. Spinning sidebands are denoted with an asterisk.

Solution NMR measurements were carried out on Bruker Avance 4.7, 5.9, 9.4, 11.7 T and 11.7 T cryoprobe spectrometers at room temperature (unless indicated otherwise). Chemical shifts δ are given as dimensionless numbers and the absolute values of the coupling constants are given in Hertz (Hz). Multiplicities are abbreviated as singlet (s), doublet (d), triplet (t), quartet (q), and broad (br). NMR spectra were referenced to TMS (^1H and ^{13}C), $CFCl_3$ (^{19}F), $Rh(acac)_3$ (^{103}Rh) and CH_3NO_2 (^{15}N). Quaternary carbons are indicated as C_q, aromatic carbon and hydrogens as C_{ar} and CH_{ar}, benzylic carbons and hydrogens as C_{benz} and CH_{benz}, olefinic carbons and hydrogens as CH_{olef} and C_{olef}, and carbene carbons as $C_{carbene}$.

1.6 Fourier-transform infrared (FT-IR) spectroscopy

FT-IR spectra were recorded on a Perkin-Elmer-Spectrum 2000 FT-IR-Raman spectrometer with KBr beam splitter (range 500–4000 cm^{-1}). For solid compounds the ATR technique was used.

1.7 X-ray photoelectron spectroscopy (XPS)

The XPS spectra were measured on a K-Alpha™ + X-ray Photoelectron Spectrometer System (Thermo Scientific) with Hemispheric 180 ° dual-focus analyzer with 128-channel detector. The X-ray monochromator used micro focused Al-Kα radiation.sorption bands are described as very strong (vs), strong (s), middle (m), weak (w), or broad (br).

1.8 Single crystal X-ray diffraction (SC-XRD) measurements

Single crystals suitable for X-ray diffraction (SC-XRD) were coated with polyisobutylene oil in a glovebox, transferred to a nylon loop and then transferred to the goniometer of a Rigaku XtaLAB Synergy-S Dual Source diffractometer with a HyPix-6000HE Detector equipped with a molybdenum X-ray tube (λ = 0.71073 Å) and a copper X-ray tube (λ = 1.5406 Å). Preliminary data was collected to determine the crystal system. The space group was identified, and the data was processed using the software CrysAlis Pro. The structures were solved using direct methods (SHELXT) on OLEX2 completed by Fourier transformation and refined by full-matrix least-squares procedures. The label 'ct' refers to the centroid of the indicated bond.

1.9 Gas chromatography (GC) thermal conductivity detector (GC-TCD) analysis

The headspace of the reactions was analyzed by gas chromatography: CO_2 was measured on a Multi-Gas Analyzer #5, SRI 8610C Instrument, equipped with Haysep D and MolSieve 5 Å columns and thermal conductivity detector (TCD) and flame ionization detector (FID) coupled with a methanizer using argon or N_2 as carrier gas. The typical volume of injected gas was 100 µL from the headspace, sampled using a gastight syringe. N_2, N_2O and H_2 was measured on an Agilent Technology 7890A GC System on a HP-Molsieve (19091P) column (19091P,15 m x 0.32 mm,25 µm) using argon as carrier gas.

1.10. High-angle annular dark-field scanning TEM (HAADF-STEM) and energy-dispersive X-ray analysis

HAADF-STEM measurements were performed on a double Cs-corrected JEOL JEM-ARM300F Grand ARM "Vortex" scanning transmission electron microscope, operated at an accelerating voltage of 300 kV. The collection angles for HAADF-STEM imaging were set to 60–180 mrad. All the samples were prepared by dry casting onto lacey carbon–coated copper TEM grids (3 mm, 2 nm thick carbon film) inside an argon glovebox, and subsequently mounted on a double-tilt vacuum transfer holder (GATAN) to ensure air-free transfer. EDX maps were acquired using a dual SDD EDX detector system (each with a 100 mm^2 active area), providing a total solid angle of 1.6 sr and an energy resolution of ≤ 133 eV.

2. Supplementary notes of syntheses

2.1 Synthesis of complexes and ligands

The complex **[Rh_2(μ-Cl)$_2$(trop$_2$NH)$_2$]**[1] and ligands: trop$_2$NH **[bis(5H-dibenzo[a,d]cycloheptene-5-yl)amine] (Trop$_2$NH)**[2], **[1,3-dimethyl-1H-benzimidazolium iodide] (BzIMeHI)**[3], and **[1,3,4,5-Tetramethyl-1,3-dihydro-2H-imidazol-2-ylidene] (MeIMe)**[4] were synthesized according to literature procedures. The N-heterocyclic imidazolium carbene salt **BzIMeHI** (0.15 mmol) was deprotonated with KHMDS (30.0 mg, 0.16 mmol) in THF (5.0 mL) for 2 hours, filtered and added directly to the metal precursor [Rh(trop$_2$NH)(THF)]OTf] (**3**).

*[Rh(trop$_2$NH)(THF)]OTf (**3**):*

Synthesized according to literature procedures.[5] In a 20 mL Schlenk flask, [Rh_2(μ-Cl)$_2$(trop$_2$NH)$_2$] (1.0 g, 0.9 mmol, 1.0 equiv) and AgOTf (482 mg, 2.19 mmol, 2.0 equiv) were dissolved in dichloromethane (DCM) or toluene (10.0 mL) and stirred at room temperature for 3 hours in the dark. The reaction mixture was filtered through a Celite plug, and the solvent was removed under reduced pressure. The resulting orange powder was washed sequentially with hexane (10.0 mL) and diethyl ether (10.0 mL). Complex **3** was recrystallized from a mixture of THF and hexane at −30 °C, which were isolated in 91% yield (1.70 mmol, 1.23 g).

^{1}H NMR (300 MHz, THF-d_8, 298 K): δ = 7.55 (d, $^3J_{HH}$ = 7.5 Hz, 2H, CH_{ar}), 7.25-7.15 (m, 6H, CH_{ar}), 7.07 (t, $^3J_{HH}$ = 7.4 Hz, 2H, CH_{ar}), 6.9-6.78 (m, 6H, CH_{ar}), 6.64 (dd, 2H, $^3J_{HH}$ = 9.4, $^2J_{RhH}$ = 2.5 Hz, CH_{olef}), 5.79 (d, $^3J_{HH}$ = 9.5 Hz, 2H, CH_{olef}), 4.58 (s, 2H, CH_{benz}), 3.97 (s, 1H, NH) ppm; ^{13}C NMR (126 MHz, THF-d_8, 298 K): δ = 140.0 (s, C_q), 137.1 (s, C_q), 135.7 (s, C_q),134.6 (s, C_q), 129.9 (s, C_{ar}), 129.3 (s, C_{ar}), 129.1 (s, C_{ar}), 129.0 (s, C_{ar}), 127.8 (s, C_{ar}), 126.6 (s, C_{ar}), 126.2 (s, C_{ar}), 125.9 (s, C_{ar}), 75.9 (d, $^1J_{RhC}$ = 13.9 Hz C_{olef}), 74.2 (d, $^1J_{RhC}$ = 8.2 Hz C_{olef}), 73.0 (s, C_{benz}) ppm; ^{19}F NMR (471 MHz, THF-d_8, 298 K): δ = - 79.5 (s, SO_3CF_3) ppm; ^{103}Rh (15.8 MHz, THF-d_8, 298 K) δ = - 6748.1 (s) ppm. Elemental analysis found for $C_{35}H_{31}F_3NO_3RhS$: C 59.1, H 4.5, (Calcd.: 58.3 , H 4.3). M.p. >150 (dec).

*[Rh(trop$_2$NH)(BzIMe)]OTf (**A**):*

Synthesized according to literature procedures.[5] The freshly formed free carbene 1,3-dimethylbenzimidazole-2-ylidene (**BzIMe**) (0.14 mmol, 1.0 equiv) was dissolved in hexane, filtered through a celite filter plug (to avoid KI contamination) and added dropwise to a red solution of complex [Rh(OTf)(Trop$_2$NH)(THF)] (**3**) (100.0 mg, 0.14 mmol) in THF (4 mL). The solution quickly turned

darker and after 16 hours the dark orange/brown precipitate was collected by decanting of the yellow solution. The precipitate was washed three times with hexane (15 mL) and dried by reduced pressure. Dark orange crystals suitable for SC-XRD were grown at room temperature from a THF/hexane layered solution. Yield = 83% (92.5 mg, 0.12 mmol).

^{1}H NMR (500 MHz, THF-d_8, 298K) δ = 7.75 (dd, $^{3}J_{HH}$ = 7.7, 1.4 Hz, 2H, CH_{ar}), 7.74 – 7.70 (m, 1H, CH_{ar}), 7.67 – 7.63 (m, 1H, CH_{ar}), 7.44 (dd, $^{3}J_{HH}$ = 7.7, 1.3 Hz, 2H, CH_{ar}), 7.42 – 7.37 (m, 2H, CH_{ar}), 7.32 (dd, $^{3}J_{HH}$ = 7.7, 1.2 Hz, 2H, CH_{ar}), 7.28 (dd, $^{3}J_{HH}$ = 7.6, 1.4 Hz, 2H, CH_{ar}), 7.24 (td, $^{3}J_{HH}$ = 7.5, 1.4 Hz, 2H, CH_{ar}), 7.17 (td, $^{3}J_{HH}$ = 7.4, 1.4 Hz, 2H, CH_{ar}), 7.02 (td, $^{3}J_{HH}$ = 7.5, 1.3 Hz, 2H, CH_{ar}), 6.89 (dd, $^{3}J_{HH}$ = 7.5, 1.3 Hz, 2H, CH_{ar}), 6.85 (dd, $^{3}J_{HH}$ = 8.9, $^{2}J_{RhH}$ = 3.3 Hz, 2H, CH_{olef}), 5.98 (s, 2H, CH_{benz}), 5.20 (d, $^{3}J_{HH}$ = 8.9 Hz, 2H, CH_{olef}), 4.94 (s, 1H, NH), 4.49 (s, 3H, NCH_3), 4.10 (s, 3H, NCH_3) ppm; ^{13}C NMR (126 MHz, THF-d_8, 298K) δ = 183.9 (d, $^{1}J_{RhC}$ = 47.1 Hz, $C_{carbene}$), 138.1 (s, C_{quart}), 138.0 (s, C_{quart}), 137.7 (s, C_{quart}), 137.4 (s, C_{quart}), 136.9 (s, C_{quart}), 136.4 (s, C_{quart}), 132.6 (s, 2C, BzIMe CH$_{ar}$), 130.1 (s, 2C, BzIMe CH$_{ar}$), 129.1 (s, CH$_{ar}$), 129.0 (s, CH$_{ar}$), 128.8 (s, CH$_{ar}$), 126.9 (s, CH$_{ar}$), 124.3 (s, CH$_{ar}$), 124.07 (s, CH$_{ar}$), 111.3 (s, CH$_{ar}$), 110.7 (s, CH$_{ar}$), 87.7 (d, $^{1}J_{RhC}$ =14.2 Hz, CH$_{olef}$), 81.1 (d, $^{1}J_{RhC}$ = 6.6 Hz, CH$_{olef}$), 71.0 (s, CH$_{benz}$), 35.3 (s, NCH$_3$), 34.7 (s, NCH$_3$) ppm; ^{1}H,^{15}N NMR (500 MHz, THF-d_8, 298K) δ = 95.0 (s, NH), 161.4 (s, N-CH$_3$) ppm; ^{103}Rh,^{1}H NMR (500 MHz, THF-d_8, 298K) δ = -6900 (s) ppm. Elemental analysis found for $C_{40}H_{33}F_3N_3O_3RhS$: C 60.9, H 4.8 (Calcd.: C 60.4, H 4.2).

*[Rh(trop$_2$NH)(MeIMe)]OTf (**B**):*

Synthesized according to literature procedures.[2] Additional NMR data in THF-d_8 is reported herein:

^{1}H NMR (500 MHz, THF-d_8, 298 K) δ = 7.61 (dd, $^{3}J_{HH}$ = 7.3, 1.7 Hz, 2H, CH_{ar}), 7.35 (dd, $^{3}J_{HH}$ = 7.2, 1.7 Hz, 2H, CH_{ar}), 7.29 (dd, $^{3}J_{HH}$ = 7.6, 1.3 Hz, 2H, CH_{ar}), 7.25 (dd, $^{3}J_{HH}$ = 7.6, 1.3 Hz, 2H, CH_{ar}), 7.22 – 7.14 (m, 4H, CH_{ar}), 6.98 (td, $^{3}J_{HH}$ = 7.5, 1.3 Hz, 2H, CH_{ar}), 6.85 (td, $^{3}J_{HH}$ = 7.5, 1.3 Hz, 2H, CH_{ar}), 6.68 (dd, $^{3}J_{HH}$ = 8.9, $^{2}J_{RhH}$ = 3.3, 2H, CH_{olef}), 5.72 (s, 2H, CH_{benz}), 5.16 (d, $^{3}J_{HH}$ = 8.9 Hz, 2H, CH_{olef}), 4.77 (s, 1H, NH), 4.10 (s, 3H, N-CH_3), 3.72 (s, 3H, N-CH_3), 2.28 (s, 3H, CH$_3$), 2.22 (s, 3H, CH$_3$) ppm.; ^{15}N NMR (51 MHz, THF-d_8, 298 K) δ = 93.0 (s, NH), 175.8 (s, NCH$_3$) ppm.

*[Rh(trop$_2$N)(BzIMe)] (**A2**):*

Synthesized according to literature procedures.[5] To a vial containing complex **A** (30 mg, 0.038 mmol. 1.0 equiv) in THF (8.0 mL), *t*BuOK (4.6 mg, 0.042 mmol, 1.1 equiv) was added at room temperature. A dark green solution was formed immediately and after 15 minutes reacting, the mixture was filtered, and the solution was layered with hexane to yield green crystals of **A2**. Yield = 87%.

^{1}H NMR (300 MHz, THF-d_8, 298 K): δ = 7.52 (m, 2H, CH_{ar}), 7.31 (m, 2H, CH_{ar}), 7.05 (d, $^{3}J_{HH}$ = 7.3 Hz, 4H, CH_{ar}), 7.00 (d, $^{3}J_{HH}$ = 7.3 Hz, 4H, CH_{ar}), 6.81 (td, $^{3}J_{HH}$ = 7.3, 1.1 Hz, 4H, CH_{ar}), 6.75 (td, $^{3}J_{HH}$ = 7.3, 1.2 Hz, 4H, CH_{ar}), 5.49 (s 'br', 4H, CH_{olef}), 4.78 (s, 2H, CH_{benz}), 4.07 (m, 6H, NCH_3) ppm; ^{13}C NMR (126.1 MHz, THF-d_8, 298K): δ = 197.0 (d, $^{1}J_{RhC}$ = 43.0 Hz, $C_{carbene}$), 145.9 (s, C_{ar}), 147.5 (s, C_{ar}), 135.6 (s, C_{ar}), 127.0 (s, C_{ar}), 125.7 (s, C_{ar}), 124.6 (s, C_{ar}), 122.3 (s, C_{ar}), 122.2 (s, C_{ar}), 119.7 (s, C_{ar}), 109.3 (s, C_{ar}), 81.7 (s, C_{benz}), 79.0 (d,$^{1}J_{RhH}$ = 13.3 Hz, C_{olef}), 33.7 (s, NCH$_3$), 25.4 (s, NCH$_3$) ppm; ^{103}Rh NMR (22.1 MHz, THF-d_8, 298 K): δ = - 7026 (s) ppm. Elemental analysis found for $C_{39}H_{33}N_3Rh$: C 71.3, H 5.9 (Calcd.: C 72.4, H 5.1).

*[Rh(trop$_2$N)(MeIMe)] (**B2**):*

Synthesized according to literature procedures.[2] Additional NMR data in THF-d_8 is reported herein:

^{103}Rh NMR (22.1 MHz, THF-d_8, 298 K): δ = -6937 (s).

*[RhH(trop$_2$NH)(BzIMe)] (**A3**):*

Synthesized according to literature procedures.[5] The color changed immediately from dark green to a lighter green to yellow color and formation of the hydride **A3** was observed after 15 minutes. Low temperature NMR (238 K) was performed to observe well defined spectra.

^{1}H NMR (500 MHz, THF-d_8, 238 K) δ = 7.54 (dd, 2H, $^3J_{HH}$ = 17.8, 7.3 Hz, CH_{ar}),7.29 (m, 2H, CH_{ar}), 7.18 (d, 2H, $^3J_{HH}$ = 7.5 Hz, CH_{ar}), 6.97 (t, 2H, $^3J_{HH}$ = 7.3 Hz, CH_{ar}), 6.93 (d, 2H, $^3J_{HH}$ = 7.3 Hz, CH_{ar}), 6.81 (m, 2H, CH_{ar}),6.75-6.57 (m, 8H, CH_{ar}),4.66 (s, 1H, NH), 4.45 (s, 2H, CH_{benz}),4.43 (s, 3H, NCH_3), 4.30 (s, 3H, NCH_3), 3.93 (d, 2H, $^3J_{HH}$ = 9.1 Hz, CH_{olef}), 3.46 (d, 2H, $^3J_{HH}$ = 8.7 Hz, CH_{olef}), - 6.10 (d, 1H, $^1J_{RhH}$ = 34.7 Hz, RhH) ppm; ^{13}C{^{1}H} NMR (126 MHz, THF-d_8, 238K): δ = 190.3 (s 'br', $C_{carbene}$), 145.0 (s, C_{ar}), 138.1 (s, C_{ar}), 137.2 (s, C_{ar}), 135.2 (s, C_{ar}), 134.2 (s, C_{ar}), 130.5 (s, C_{ar}), 129.9 (s, C_{ar}), 128.0 (s, C_{ar}), 127.3 (s, C_{ar}), 126.7 (s, C_{ar}), 126.2 (s, C_{ar}), 126.1 (s, C_{ar}), 125.2 (s, C_{ar}),122.9 (s, C_{ar}),122.6 (s, C_{ar}), 122.2 (s, C_{ar}), 119.0 (s, C_{ar}), 110.1 (s, C_{ar}), 109.6 (s, C_{ar}), 71.9 (s, C_{benz}),54.3 (s 'br', C_{olef}), 52.0 (s 'br', C_{olef}), 36.9 (s, NCH_3), 36.5 (s, NCH_3) ppm.

*[RhH(trop$_2$NH)(MeIMe)] (**B3**):*

The spectroscopic NMR data of complex **B3** is in agreement with previously published results.[6] Additional characterization data is reported herein. Yellow single crystals suitable for SC-XRD analysis were grown from layering a saturated solution of **B3** and H_2 in THF with hexane.

^{15}N NMR (51 MHz, THF-d_8, 298 K) δ = 71.1 (NH), 177.7 (NCH$_3$). ATR IR (ν in cm^{-1}): 2917 s, 2849 s, 1565 m, 1458 m, 1369 m, 1156 w, 1085 w, 798 s, 747 s, 610 m, 529 m.

*[Rh(tropNHtrop')(MeIMe)] (**B-CH**):*

In a glovebox a J. Young NMR tube was loaded with [Rh(trop$_2$N)(MeIMe)] (**B2**) (20 mg, 0.014 mmol) and dissolved in THF-d_8 (0.5 mL), before it was transferred outside and degassed with three freeze-pump-thaw cycles. Under reduced pressure, the tube was charged with H_2 (1.0 bars) and mixed for 16 hours. The resulting dark red solution was dried by reduced pressure, and **B-CH** was extracted with hexane (3.0 mL). Dark red crystals suitable for SC-XRD were grown from slow evaporation of the hexane solution at room temperature, yield = 82 %. [Symmetry notes: Trop-moieties are oriented in an exo-exo conformation but with one trop twisted 90 degrees around the N-trop bond, which gives similar coupling as the exo-endo conformer of the free bis-trop-amine ligand].

^{1}H NMR (500 MHz, THF-d_8) δ = 7.50–7.41 (m, 2H, CH_{ar}), 7.38 (d, $^3J_{HH}$ = 7.6, 1H, CH_{ar}), 7.34–7.23 (m, 3H, CH_{ar}), 7.21–6.98 (m, 6H, CH_{ar}), 6.95 (d, $^3J_{HH}$ = 7.5, 1H, CH_{ar}), 6.37 (t, $^3J_{HH}$ = 7.1, 1H, CH_{ar}), 6.28 (d, $^3J_{HH}$ = 7.1, 1H, CH_{ar}), 6.21 (d, $^3J_{HH}$ = 6.8, 1H, CH_{ar}), 5.59 (d, $^3J_{HH}$ = 11.3, 1H, NH), 5.25 (d, $^3J_{HH}$ = 11.2, 1H, $CH_{benzylic}$), 4.94 (s, 1H, $CH_{benzylic}$), 4.15 (s, 3H, N-CH$_3$), 4.12 (d, $^3J_{HH}$ = 8.0, 1H, CH_{olef}), 3.69 (d, $^3J_{HH}$ = 8.6, 1H, CH_{olef}), 3.62 (s, 3H, N-CH$_3$), 3.26 (td, $^3J_{HH}$ = 13.7, 3.5, 1H, CH$_2$), 2.89 (d, $^3J_{HH}$ = 16.8, 1H, CH$_2$), 2.74 (t, $^3J_{HH}$ = 15.2, 1H, CH$_2$), 2.66 (dt, $^3J_{HH}$ = 13.3, 3.8, 1H, CH$_2$), 2.21 (s, 3H, CH$_3$), 2.14 (s, 3H, CH$_3$). ^{13}C NMR (126 MHz, THF-d_8) δ = 192.90 (d, $^1J_{RhC}$ = 63.5, $C_{NHC\text{-}carbene}$), 165.80 (d, $^1J_{RhC}$ = 45.0, $C_{anionic\text{-}carbene}$), 153.19 ($C_q$), 146.09 ($C_q$), 143.65 (d, $^1J_{RhC}$ = 2.2, C_q), 143.31 (C_q), 140.92 (C_q), 137.99 (d, $^1J_{RhC}$ = 3.5, CH_{ar}), 136.51 (C_q), 136.08 (d, $^1J_{RhC}$ = 1.9, C_q), 132.77 (C_q), 130.83 (CH_{ar}), 129.20 (CH_{ar}), 129.06 (CH_{ar}), 128.80 (CH_{ar}), 128.77 (CH_{ar}), 128.13 (CH_{ar}), 127.68 (CH_{ar}), 126.95 (CH_{ar}), 126.70 (CH_{ar}), 126.58 (CH_{ar}), 124.63 (CH^{ar}), 124.27 (CH_{ar}),

124.19 (C_q, NHC), 124.04 (C_q, NHC), 124.02 (CH_{ar}), 123.98 (CH_{ar}), 67.7 ($CH_{benzylic}$) 65.97 (d, $^1J_{RhC}$ = 11.5, CH_{olefin}), 65.33 (d, $^1J_{RhC}$ = 2.2, 2C, C), 58.73 (d, $^1J_{RhC}$ = 8.4, CH_{olefin}), 35.89 (CH_2), 35.71 (N-CH_3), 35.45 (N-CH_3), 32.59 (CH_2), 32.35 (CH_2), 9.23 (2C, CH_3). ^{1}H,^{15}N NMR (500 MHz, THF-d_8, 298k) δ = 66.5 (s, NH), 173.0 (s, N-CH_3). ^{103}Rh,^{1}H NMR (500 MHz, THF-d_8, 298k) δ = −7424 (s).

*[Rh(trop$_2$N)(MeIMe)] (**B-CO$_2$**):*

In the glovebox a J. Young NMR tube was loaded with [Rh(trop$_2$N)(MeIMe)] (**B2**) (20 mg, 0.014 mmol) and dissolved in THF-d_8 (0.5 mL), before it was transferred outside and degassed with three freeze-pump-thaw cycles. Under reduced pressure, the tube was charged with N_2O, $^{13}CO_2$, and H_2 (0.5 bars of each) and mixed for 48 hours at room temperature. The resulting light orange solution was cooled to −30 °C and crystals of **B-CO$_2$** were grown, suitable for SC-XRD, yield = 76 %. [Symmetry notes: Trop-moieties are oriented in an exo-exo conformation but with one trop twisted 90 degrees around the N-trop bond, which gives similar coupling as the exo-endo conformer of the free bis-trop-amine ligand].

^{1}H NMR (500 MHz, THF-d_8, 298 k) δ = 7.98 (d, $^3J_{HH}$ = 7.35, 1H, CH_{ar}), 7.59 (d, $^3J_{HH}$ = 6.65, 1H, CH_{ar}), 7.43–7.37 (m, 1H, CH_{HCOO}), 7.31–7.19 (m, 4H, CH_{ar}), 7.17–7.09 (m, 2H, CH_{ar}), 7.09–7.04 (m, 2H, CH_{ar}), 6.99 (t, $^3J_{HH}$ = 7.02, 1H, CH_{ar}), 6.95–6.85 (m, 3H, CH_{ar}), 6.85–6.76 (m, 2H, CH_{ar}), 6.29 (d, $^3J_{HH}$ = 11.25, 1H, NH), 4.45 (s, 1H, $CH_{benzylic}$), 4.31 (d, $^3J_{HH}$ = 11.5, 1H, $CH_{benzylic}$), 4.20 (s, 3H, N-CH_3), 4.04 (td, $^3J_{HH}$ = 14.05, 3.90, 1H, CH_2), 3.82 (s, 3H, N-CH_3), 3.37 (dd, $^3J_{HH}$ = 8.15, $^1J_{RhH}$ = 1.85, 1H, CH_{olefin}), 3.22 (dt, $^3J_{HH}$ = 17.25, 3.80, 1H, CH_2), 2.87 (d, $^3J_{HH}$ = 8.00, 1H, CH_{olefin}), 2.70 (td, $^3J_{HH}$ = 14.25, 4.05, 1H, CH_2), 2.62 (dt, $^3J_{HH}$ = 14.55, 3.95, 1H, CH_2), 4.20 (s, 1H, N-CH_3), 2.11 (s, 3H, CH_3), 2.05 (s, 3H, CH_3) ppm; ^{13}C NMR (126 MHz, THF-d_8, 298 k) δ = 176.35 (d, $^1J_{RhC}$ = 55.7, $C_{NHC\text{-}carbene}$), 166.08 (C_{HCOO}), 143.74 (C_q),142.70 (C_q), 141.60 (C_q),140.50 (C_q), 137.95 (C_q), 136.75 (C_q), 134.61 (C_q), 133.23 (C_q), 131.47 (C_q), 131.43 (C_{ar}), 131.02 (C_{ar}), 130.52 (C_q), 129.23 (C_{ar}), 128.48 (C_{ar}), 128.40 (C_{ar}), 128.18 (C_{ar}), 127.90 (C_{ar}), 127.65 (C_{ar}), 126.98 (C_{ar}), 126.57 (C_{ar}), 124.03 (C_{ar}), 123.00 (C_{ar}), 68.81 ($C_{benzylic}$), 65.36 ($C_{benzylic}$), 51.41 (d, $^1J_{RhC}$ = 19.8, C_{olefin}), 39.95 (d, $^1J_{RhC}$ = 16.8, C_{olefin}), 34.37 ($C_{N\text{-}CH3}$), 33.97 (2C, C_{CH2}), 33.82 ($C_{N\text{-}CH3}$), 30.79 (2C, C_{CH2}), 7.88 ($C_{NHC\text{-}CH3}$), 7.85 ($C_{NHC\text{-}CH3}$) ppm; ^{1}H,^{15}N NMR (500 MHz, THF-d_8, 298k) δ = 59.0 (s, NH), 175.7 (s, N-CH_3) ppm; ^{103}Rh,^{1}H NMR (500 MHz, THF-d_8, 298k) δ = −6550 (s) ppm.

2.2 Synthesis of monomers

4,7-dibromo-1H-benzo[d]imidazole:

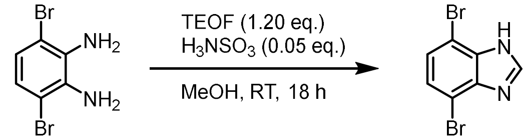

The benzimidazole was synthesized according to literature procedure[7] 3,6-dibromo-1,2-benzenediamine (2.00 g, 7.52 mmol, 1.00 eq.) was dissolved in methanol (35 mL) before triethyl orthoformate (TEOF, 1.34 g, 9.02 mmol, 1.20 eq.) and sulfamic acid (36.5 mg, 0.376 mmol, 0.05 eq.) were added and stirred for 18 h at room temperature. The volatile organic compounds were removed under reduced pressure and the off-white solid was washed with diethyl ether. After drying under reduced pressure, the product was obtained as an off-white powder (1.90 g, 6.89 mmol, 92%).

^{1}H-NMR (200 MHz, DMSO-d_6, 25 °C): δ = 13.24 (br s, 1H), 8.40 (s, 1H), 7.38 (s, 2H) ppm.

*4,7-dibromo-1-methyl-1H-benzo[d]imidazole (**1**)*:

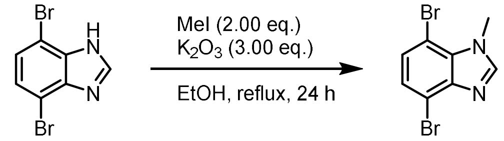

The compound was prepared according to literature.[7] 4,7-dibromo-1*H*-benzo[d]imidazole (1.60 g, 5.80 mmol, 1.00 eq) was dissolved in ethanol (40 mL). After the addition of potassium carbonate (2.40 g, 17.4 mmol, 3.00 eq) methyl iodide (1.65 g, 11.6 mmol, 0.72 mL, 2.00 eq) was added dropwise to the solution. Upon complete addition the reaction was stirred for 20 h under reflux. After cooling the reaction mixture to room temperature 20 ml of water was added. Volatiles were removed under reduced pressure before the aqueous phase was extracted three times with dichloromethane. The organic phase was dried over $MgSO_4$ and volatiles removed under reduced pressure. The product was obtained as off-white product (1.18 g, 4.07 mmol, 70%).

^{1}H NMR (200 MHz, DMSO-d_6, 25 °C): δ = 8.35 (s, 1H), 7.36 (s, 2H), 4.09 (s, 3H) ppm.

2.3 Synthesis of copolymer

Im-POP-3000:

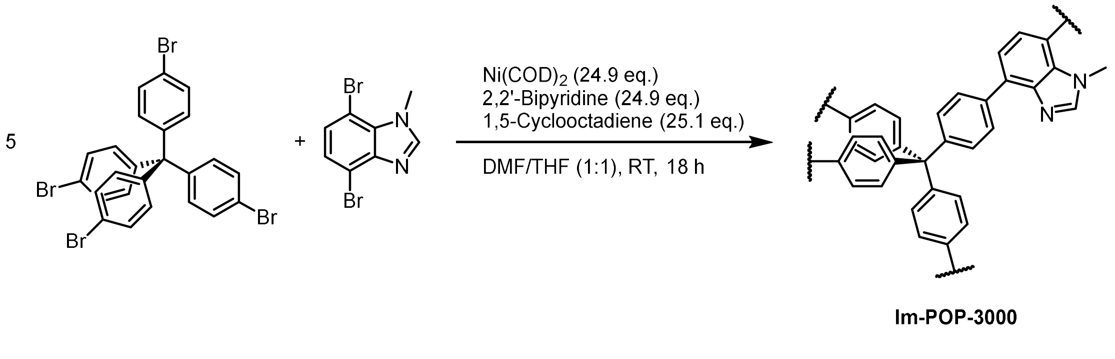

Im-POP-3000

Under argon atmosphere, an oven-dried flask was charged with bis(cyclooctadiene)nickel (1.287 g, 4.68 mmol, 24.9 eq.), 2,2'-bipyridine (731 mg, 4.68 mmol, 24.9 eq.) and 1,5-cyclooctadien (511 mg, 0.58 ml, 4.72 mmol, 25.1 eq.) followed by the addition of dimethylformamide (70 ml) and tetrahydrofurane (70 ml). The solution was stirred for 10 min before a homogenized mixture of tetrakis(4-bromphenyl)methane (600 mg, 0.94 mmol, 5.00 eq.) and 4,7-dibromo-1-methyl-1H-benzo[d]imidazole (55 mg, 0.19 mmol, 1.00 eq.) was added under stirring. The reaction was stirred for 18 h at room temperature. The reaction was cooled to 0 °C and stopped by the dropwise addition of HCl (3 M, 160 ml) over 2 h. The resulting suspension was filtered and washed with dest. water and methanol. The obtained solid was washed with hot methanol for 48 h through Soxhlet extraction. After drying under reduced pressure (10^{-3} mbar, 80 °C) for 12 h **Im-POP-3000** was obtained as an off-white powder (314 mg, 1.10 mmol, 97%).

^{13}C CP/MAS NMR (100 MHz, 7 kHz, 298 k): δ = 145, 139, 130, 125, 64, 31 ppm. SA_{BET}: 3608 m^2 g^{-1}.

preNHC-POP-3000:

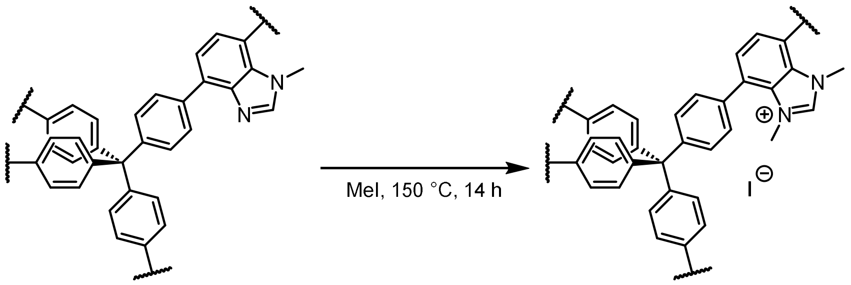

Im-POP-3000 **preNHC-POP-3000**

Im-POP-3000 (300 mg, 1.05 mmol) was suspended in methyl iodide (15 ml) and the reaction mixture heated to 150 °C for 14 h. After cooling down to room temperature the suspension was filtered and the solid washed with hot methanol through Soxhlet extraction for 24 h. The solid was dried under reduced pressure (10^{-3} mbar, 80 °C) to give **preNHC-POP-3000** as light-yellow solid (320 mg, 1.04 mmol, 99%).

^{13}C CP/MAS NMR (100 MHz, 7 kHz, 298 k): δ = 145, 139, 130, 125, 64, 35 ppm. SA_{BET}: 3110 m^2 g^{-1}.

2.4 Immobilization of complex on polymer

NHC-POP-[Rh-NH]:

In an argon filled glovebox, to a vial with **preNHC-POP-3000** (100.0 mg, ca. 0.053 mmol) suspended in THF (8.0 mL) LiHMDS (13.5 mg, 0.080 mmol) in THF (2.0 mL) was added dropwise under constant stirring. After reacting for 3 hours the mixture was ultrasonicated (30 minutes) and reacted for an additional 3 hours. The solid was filtrated off and washed with THF (40.0 mL) and hexane (10.0 mL) before being dried under reduced pressure, affording the pinkish powder **NHC-POP-3000**. The freshly formed **NHC-POP-3000** was suspended in THF (5.0 mL) and added dropwise to a solution containing the complex [Rh(trop$_2$NH)(THF)]OTf (**3**) (50.0 mg, 0.069 mmol, ca. 1.3 equiv) in THF (15.0 mL) in a Schlenk flask (Normac). The reaction was mixed for 16 hours and ultrasonication (15 minutes) was performed after 1h and again after and 15 h of reaction time. The solid was filtrated off and washed three times with THF (20.0 mL) and one time with hexane (10.0 mL) before being dried under reduced

pressure, affording the orange silver-free powder **NHC-POP-[Rh-NH]** (108.0 mg, ca. 0.46 mmol, 85%) (Figure S28). This material is air- and moisture stable, but kept stored under argon.

^{13}C CP/MAS NMR (100 MHz, 13 kHz, 298 k): δ = 145, 137, 130, 126, 74, 64, 37 ppm. SA_{BET}: 1519 m^2 g^{-1}. ICP-OES: 2.4–3.4 wt% [Rh].

2.5 Activation of catalyst

NHC-POP-[Rh-N]:

In an argon filled glovebox, a J-Young flask with **NHC-POP-[Rh-NH]** (100.0 mg, 0.028 mmol [Rh]) was suspended in cold THF (15.0 mL, ca. −30 °C). A separate cold solution of LiHMDS (13.5 mg, 0.080 mmol) in THF (5.0 mL, ca. −30 °C) was added dropwise over ca. 3 minutes under constant stirring, causing the suspension to turn green. The flask was removed from the glovebox, ultrasonicated for 5 minutes, and stirred for another 5 minutes while kept cold (< 0 °C). It was then returned to the glovebox for filtration. The solid was washed with THF (40.0 mL) and hexane (10.0 mL) before being dried by reduced pressure, affording the green powder **NHC-POP-[Rh-N]** (90.0 mg, ca. 0.040 mmol, 96% yield). This material is air- and moisture sensitive and is stored under argon in a freezer.

^{13}C CP/MAS NMR (100 MHz, 13 kHz, 298 k): δ = 145, 138, 131, 126, 82, 74, 64, 37 ppm. ICP-OES: 2.4–3.4 wt% [Rh].

3. Supplementary figures of liquid NMR spectra

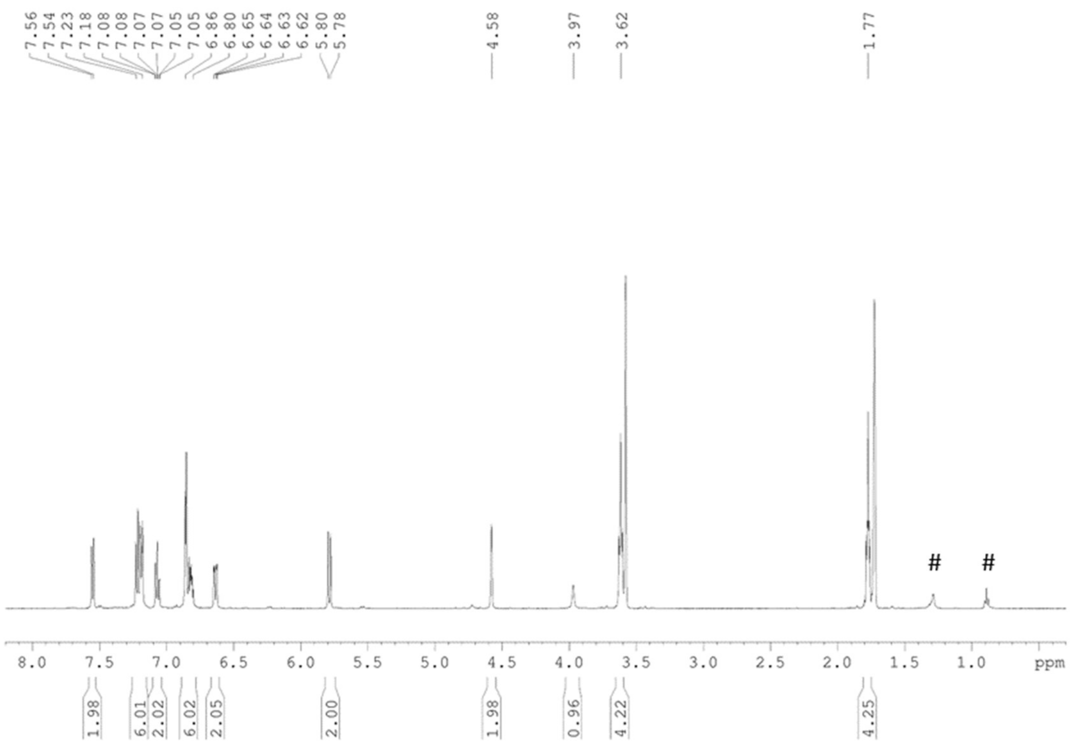

Figure S 1. ^{1}H NMR (500MHz, THF-d_8, 298 K) spectrum of complex **3**. Residual hexane (#).

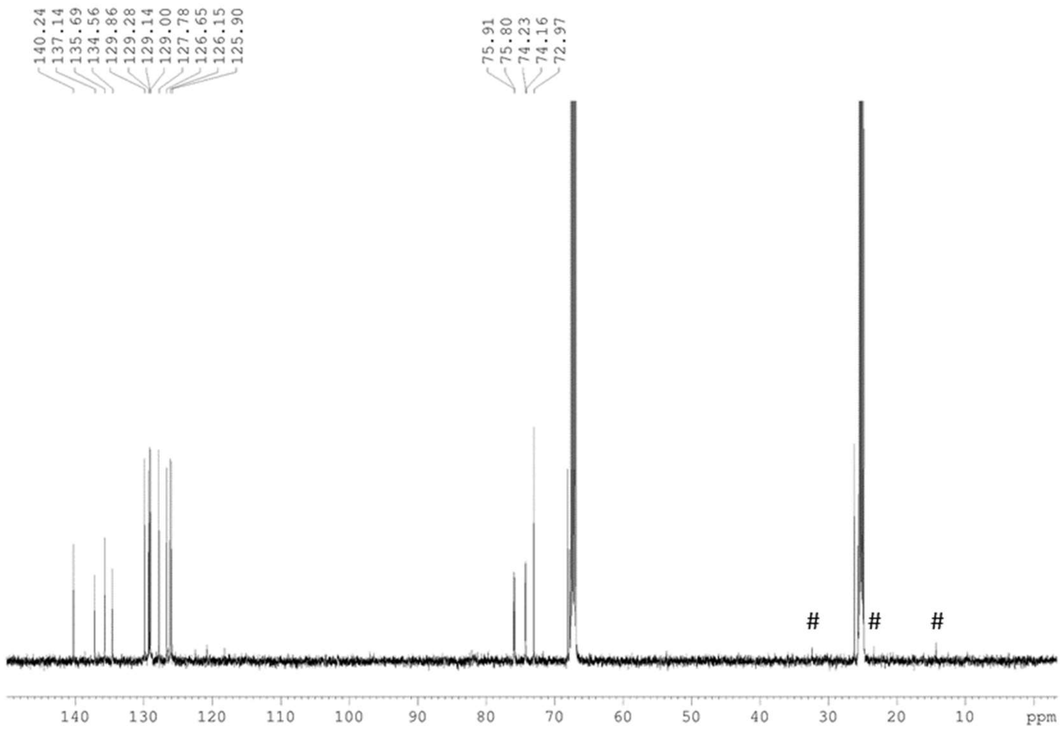

Figure S 2. ^{13}C NMR (126MHz, THF-d_8, 298 K) spectrum of complex **3**. Residual hexane (#).

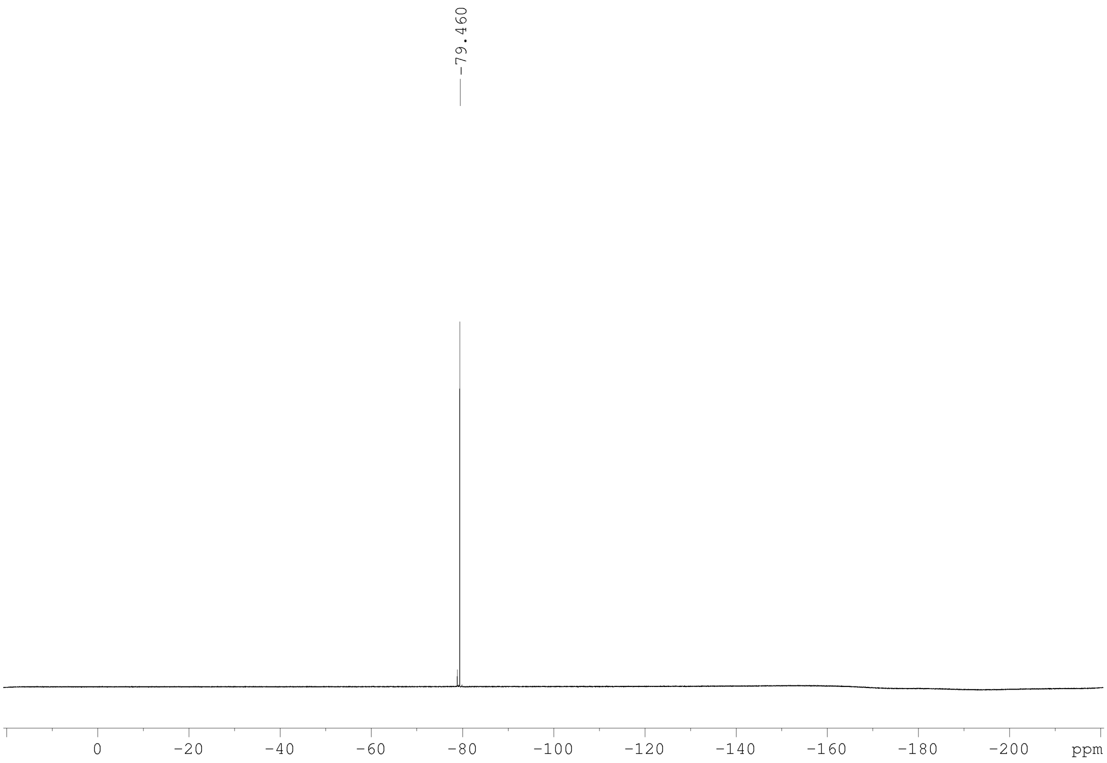

Figure S 3. ^{19}F NMR (471MHz, THF-d_8, 298 K) spectrum of complex **3**.

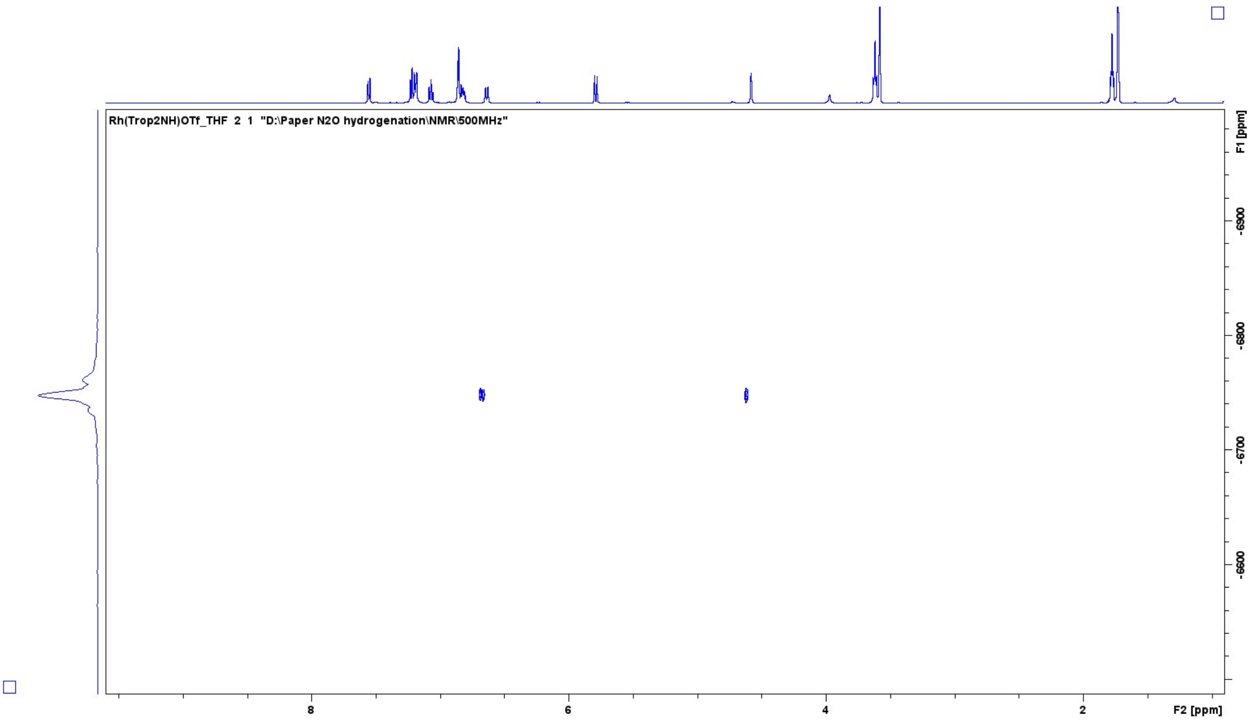

Figure S 4. ^{1}H-^{103}Rh HMBC NMR (15.8MHz, THF-d_8, 298 K) spectrum of complex **3**.

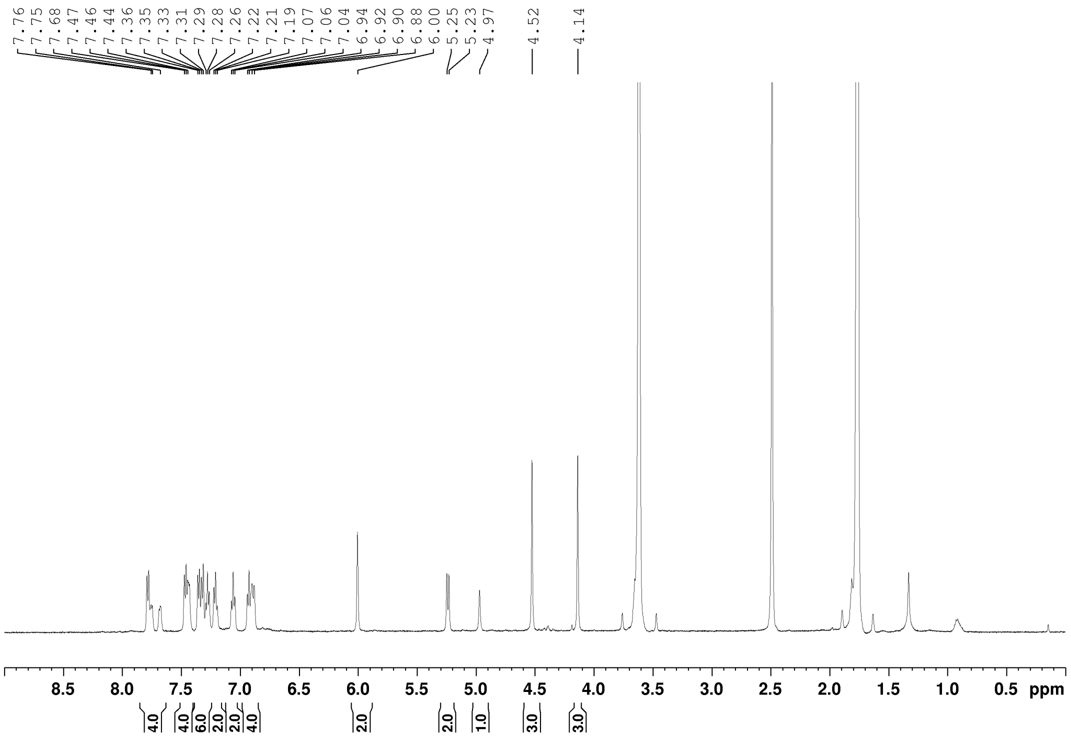

Figure S 5. ^{1}H NMR (500MHz, THF-d_8, 298 K) spectrum of complex **A**.

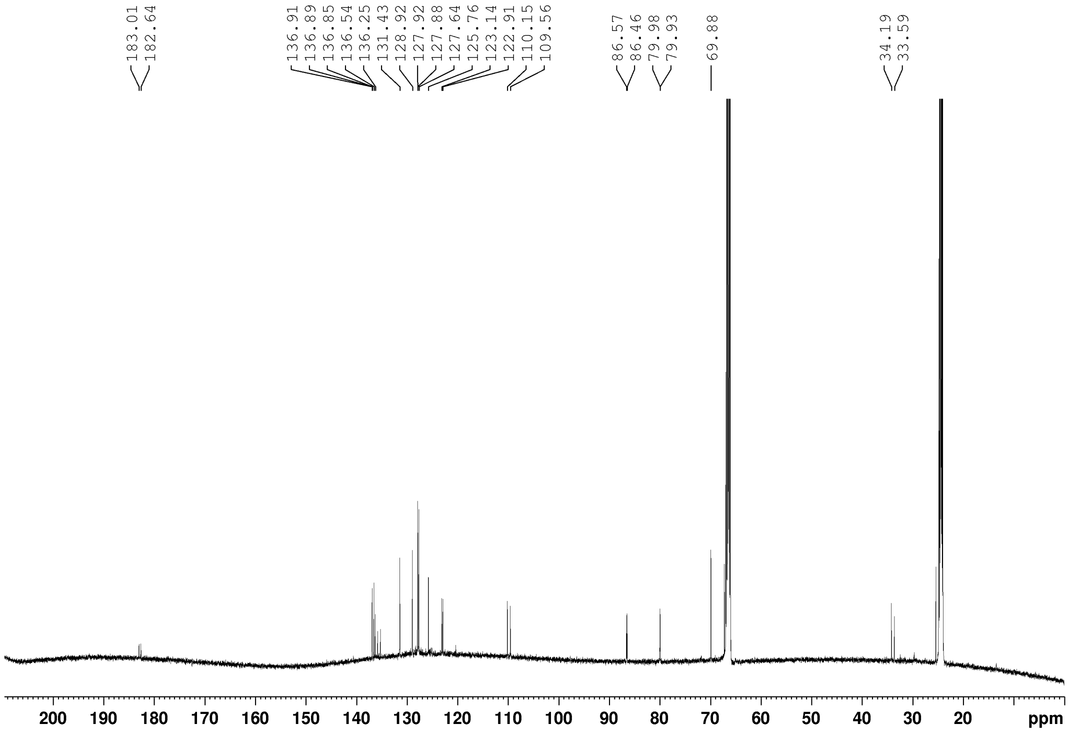

Figure S 6 ^{13}C NMR (126MHz, THF-d_8, 298 K) spectrum of complex **A**.

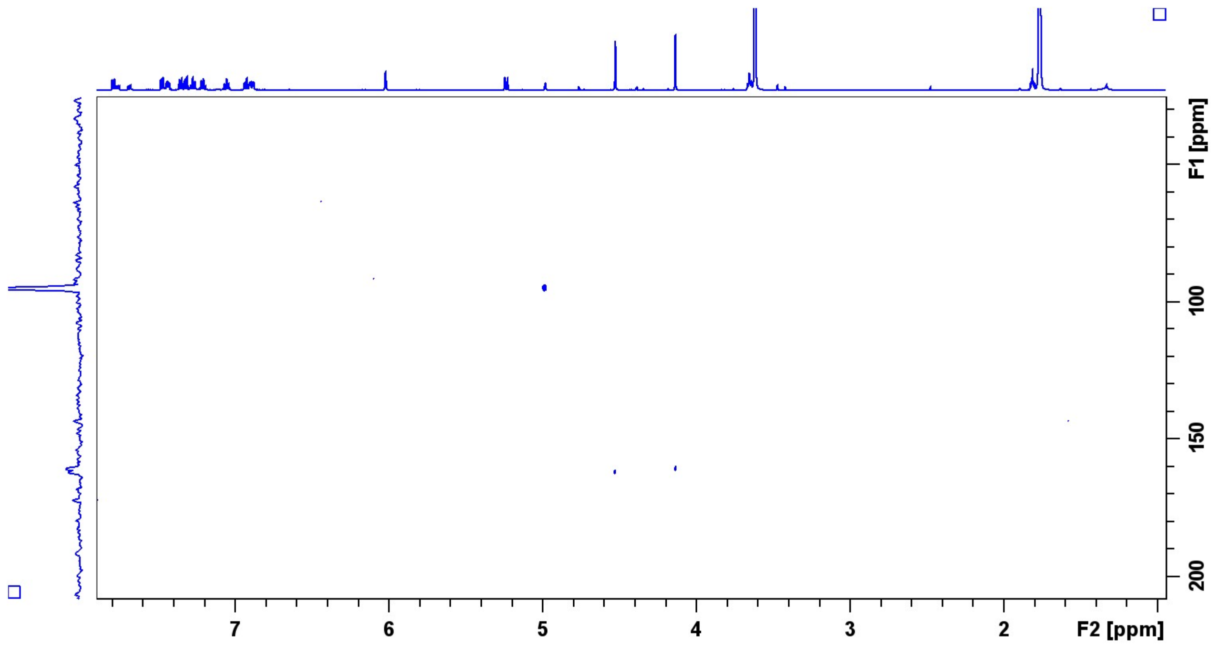

Figure S 7. ^{1}H-^{15}N HSQC NMR (51MHz, THF-d_8, 298 K) spectrum of complex **A**.

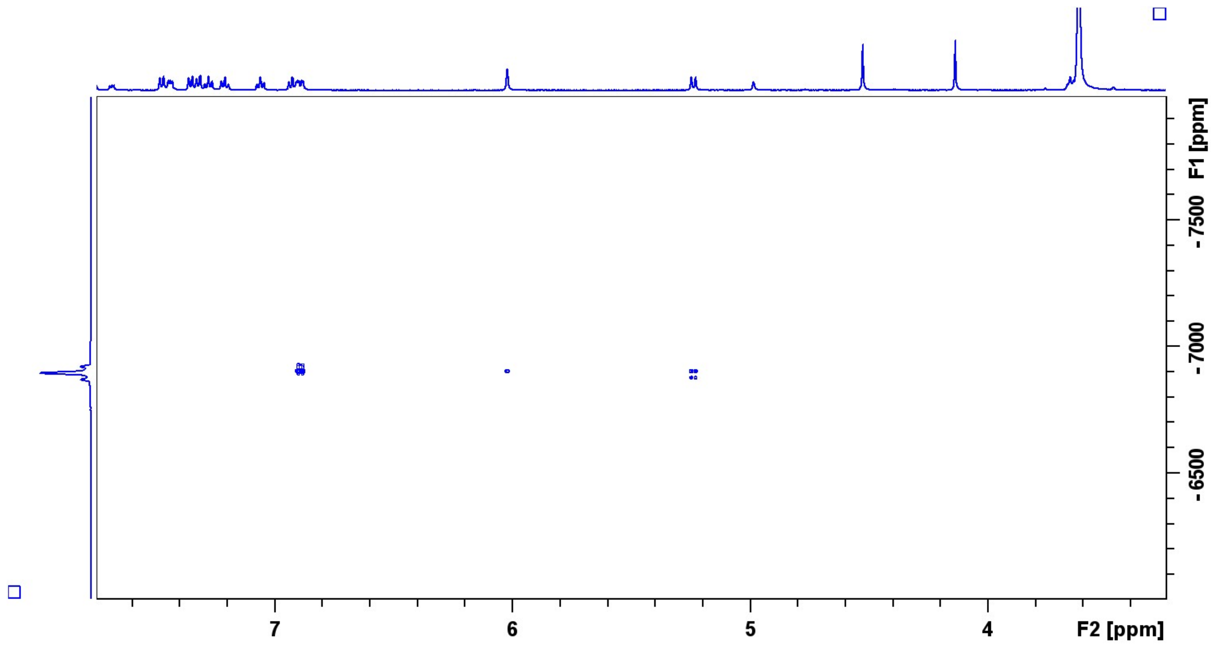

Figure S 8. ^{1}H-^{103}Rh HMBC NMR (15.8MHz, THF-d_8, 298 K) spectrum of complex **A**.

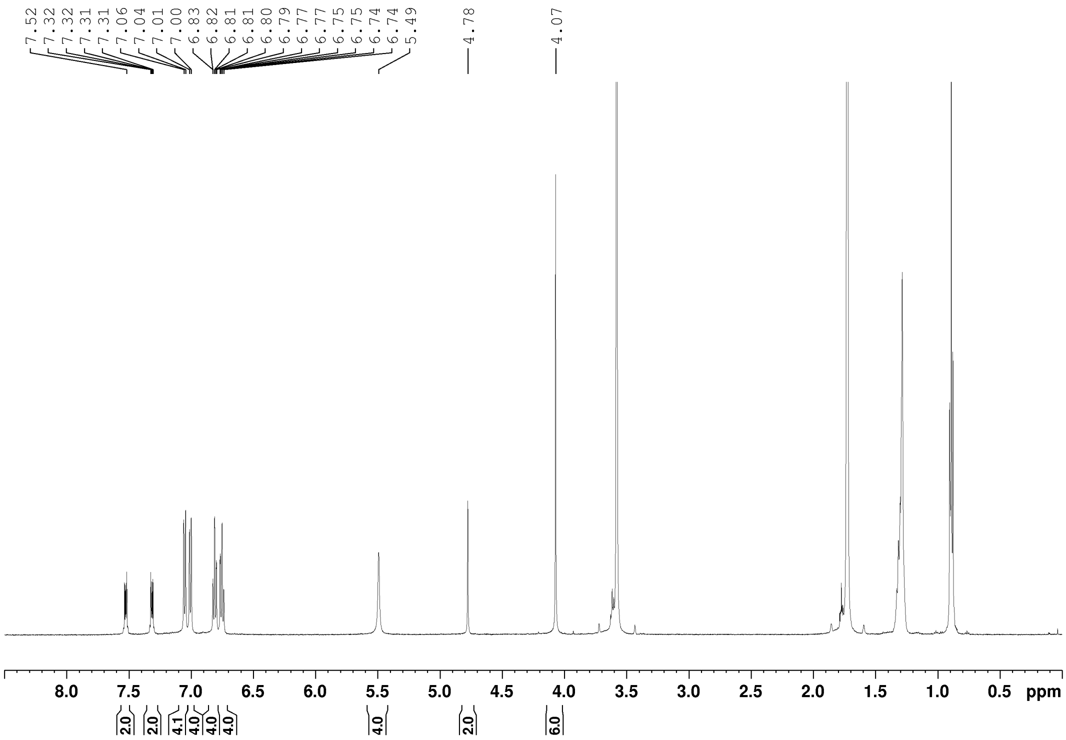

Figure S 9. ^{1}H NMR (500MHz, THF-d_8, 298 K) spectrum of complex **A2**.

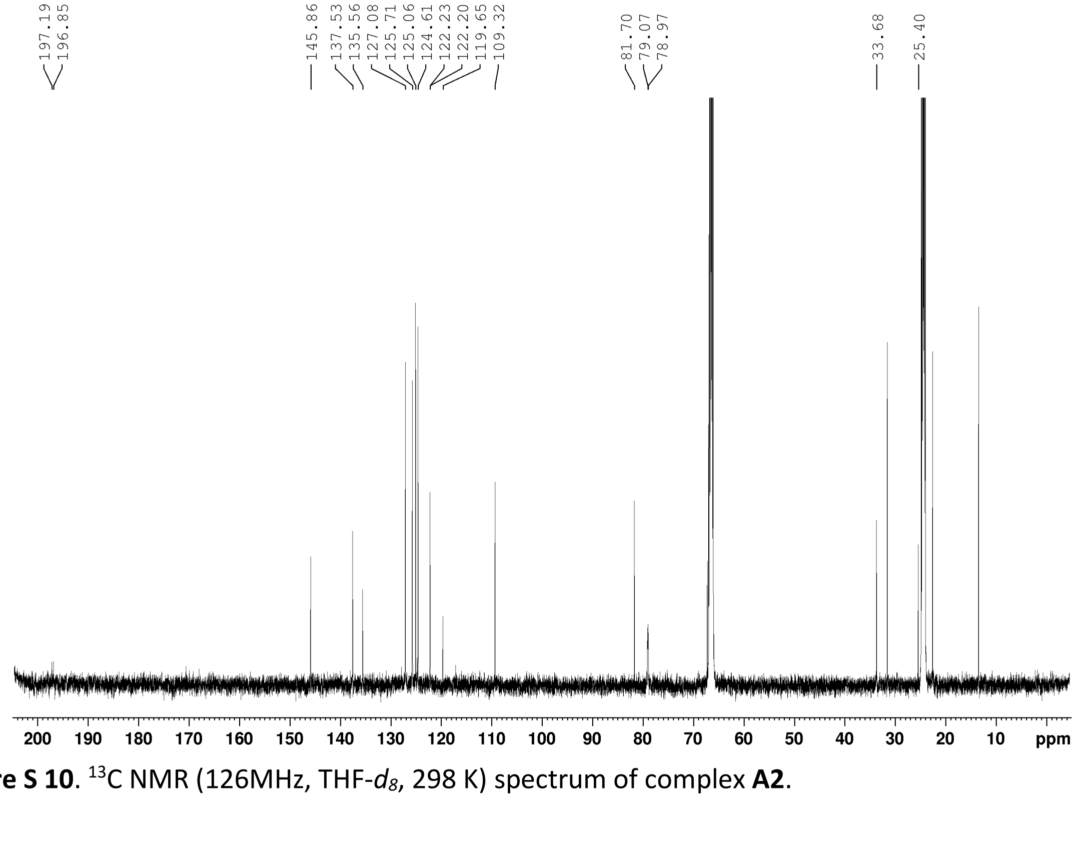

Figure S 10. ^{13}C NMR (126MHz, THF-d_8, 298 K) spectrum of complex **A2**.

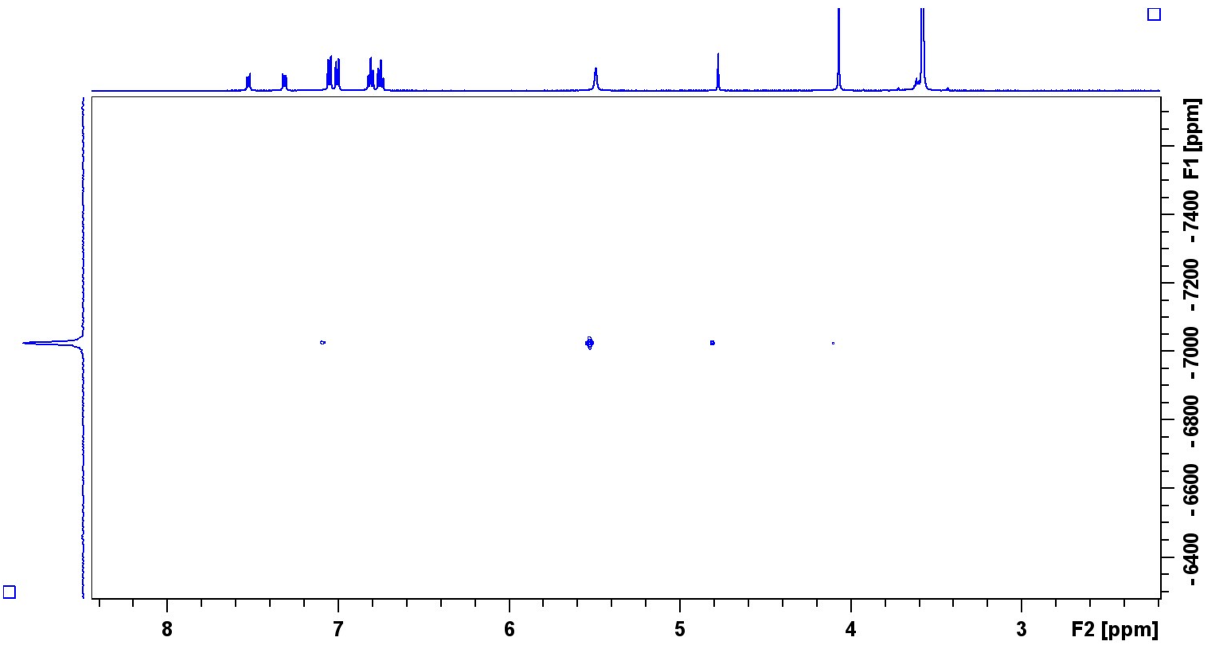

Figure S 11. ^{1}H-^{103}Rh HMBC NMR (15.8MHz, THF-d_8, 298 K) spectrum of complex **A2**.

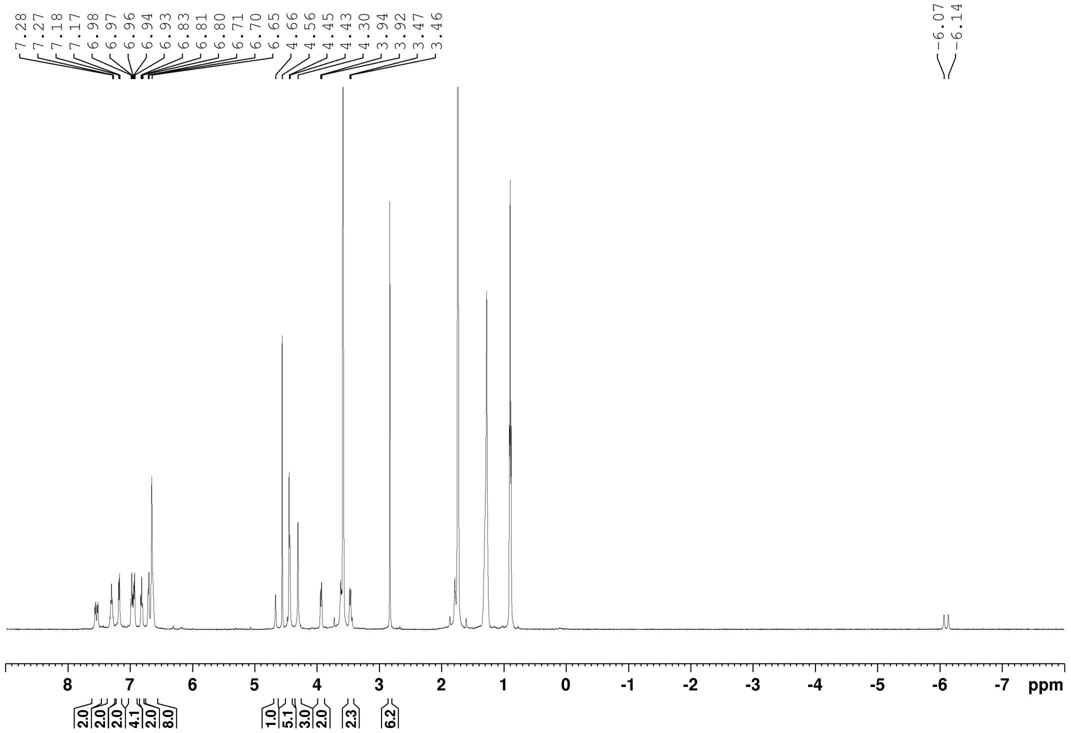

Figure S 12. ^{1}H NMR (500MHz, THF-d_8, 238 K) spectrum of complex **A3**.

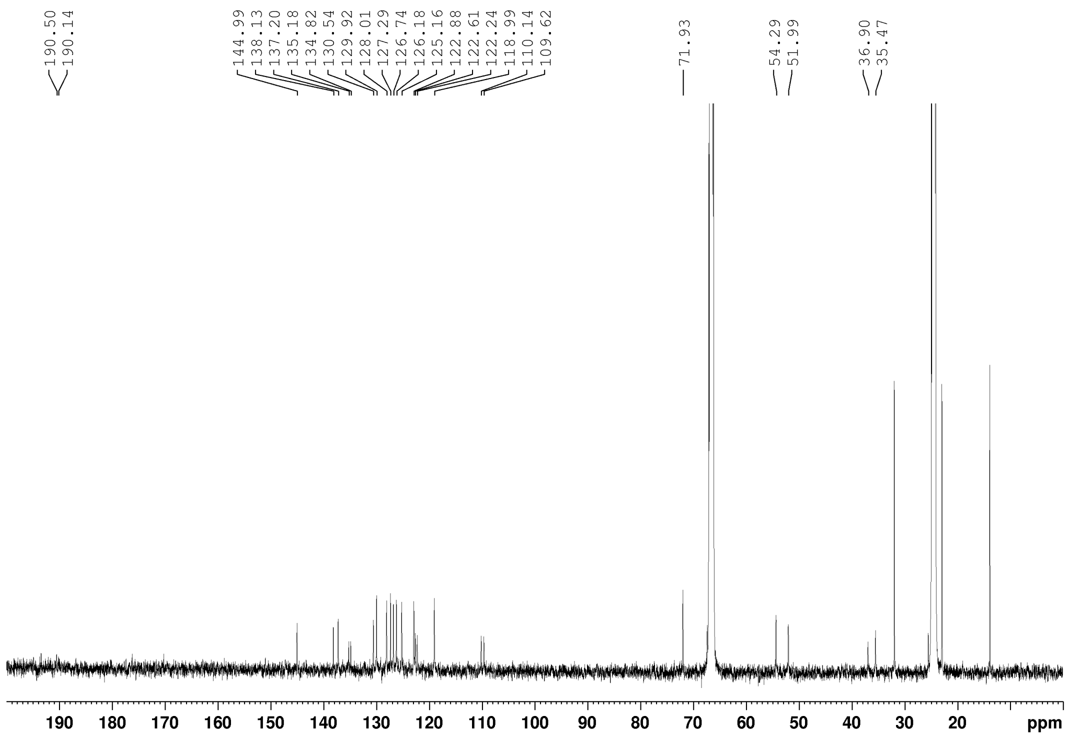

Figure S 13. ^{13}C NMR (126MHz, THF-d_8, 238 K) spectrum of complex **A3**.

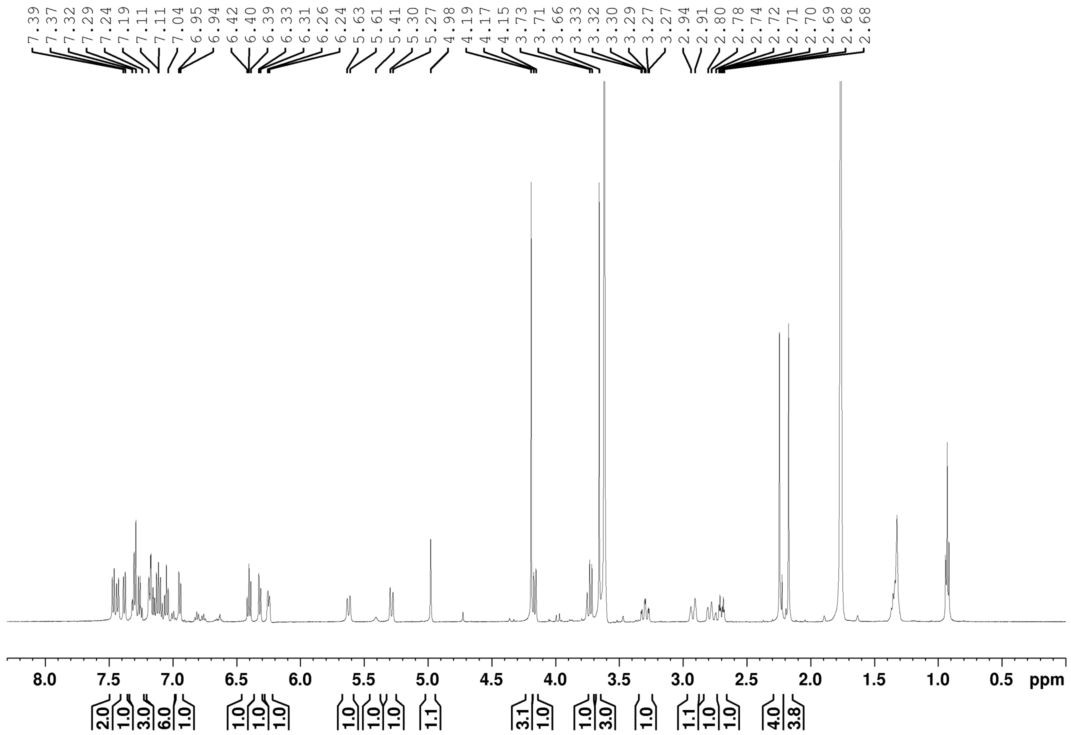

Figure S14. ^{1}H NMR (500MHz, THF-d_8, 298 K) spectrum of complex **B-CH**.

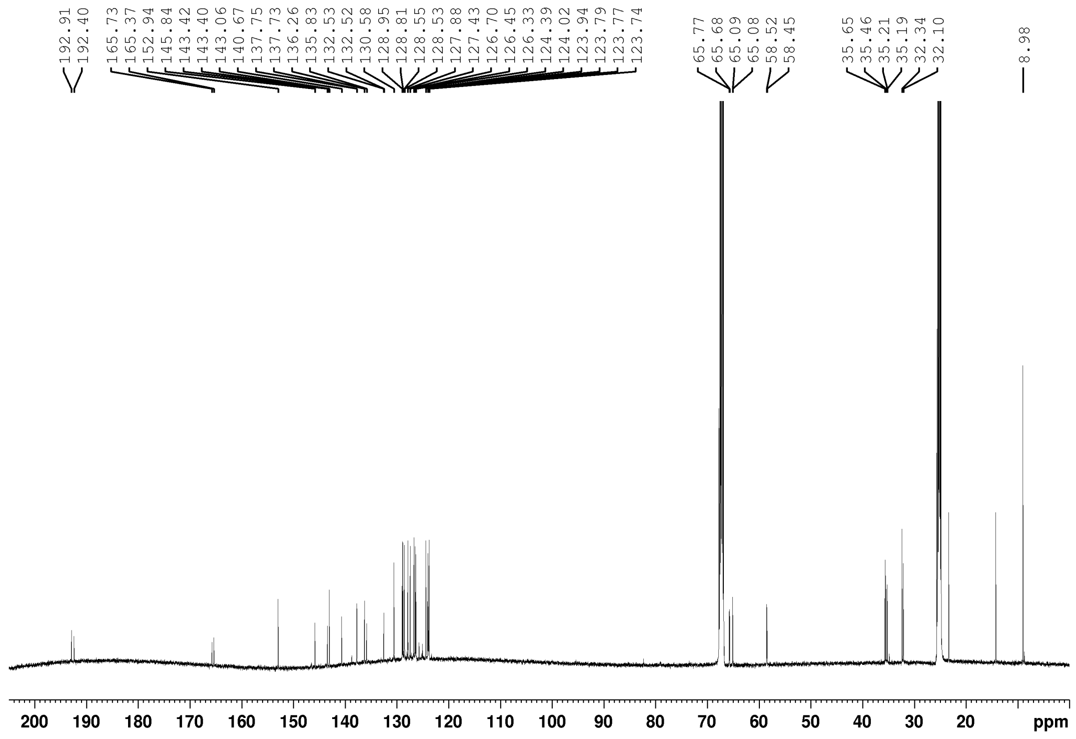

Figure S15. ^{13}C NMR (126MHz, THF-d_8, 298 K) spectrum of **B-CH**.

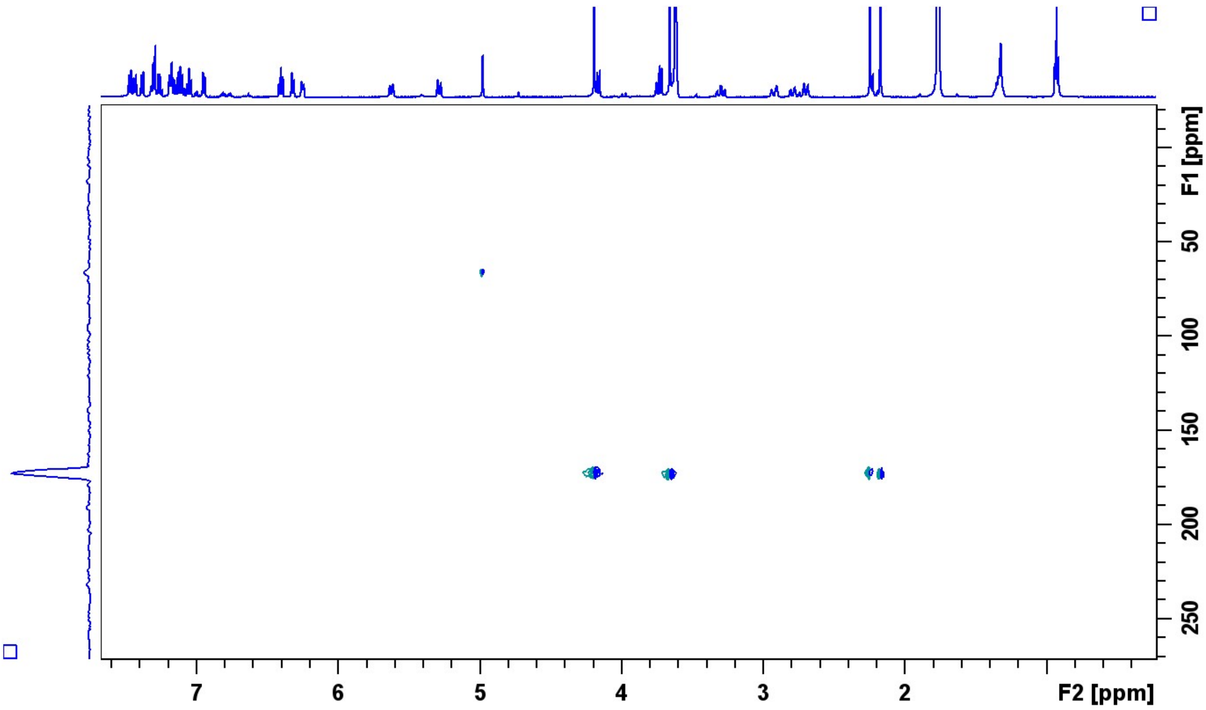

Figure S16. ^{1}H-^{15}N HSQC NMR (51MHz, THF-d_8, 298 K) spectrum of **B-CH**.

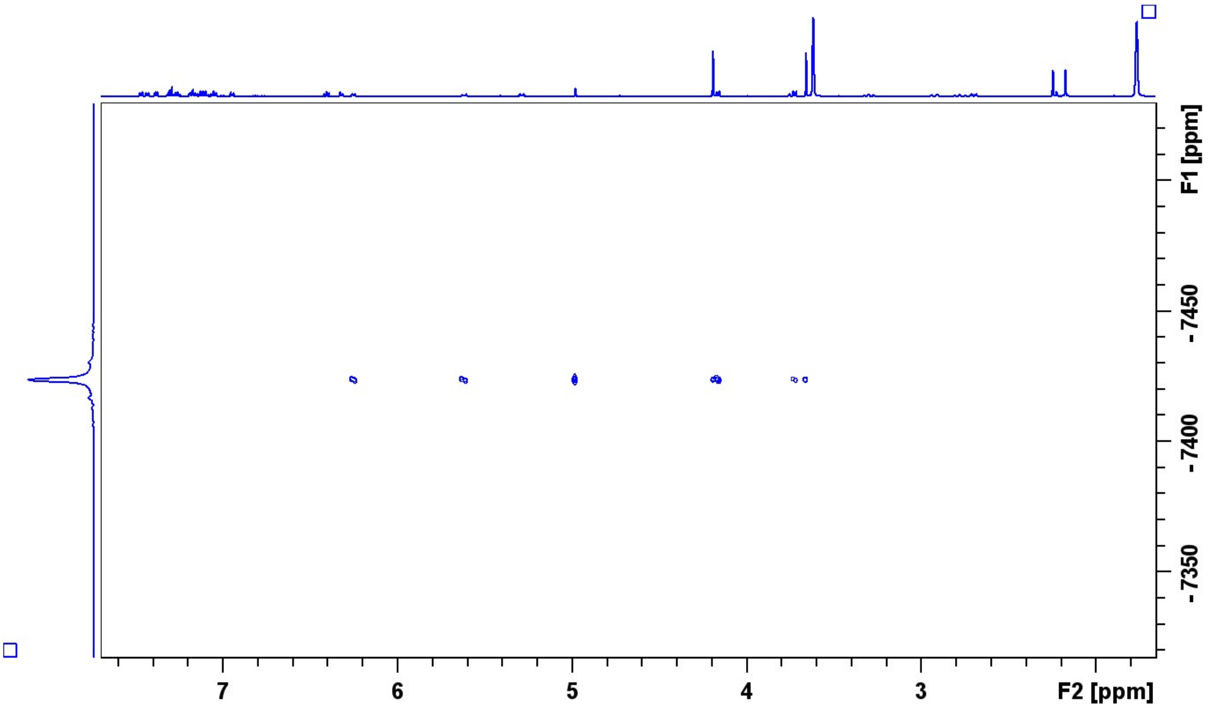

Figure S17. ^{1}H-^{103}Rh HMBC NMR (15.8MHz, THF-d_8, 298 K) spectrum of **B-CH**.

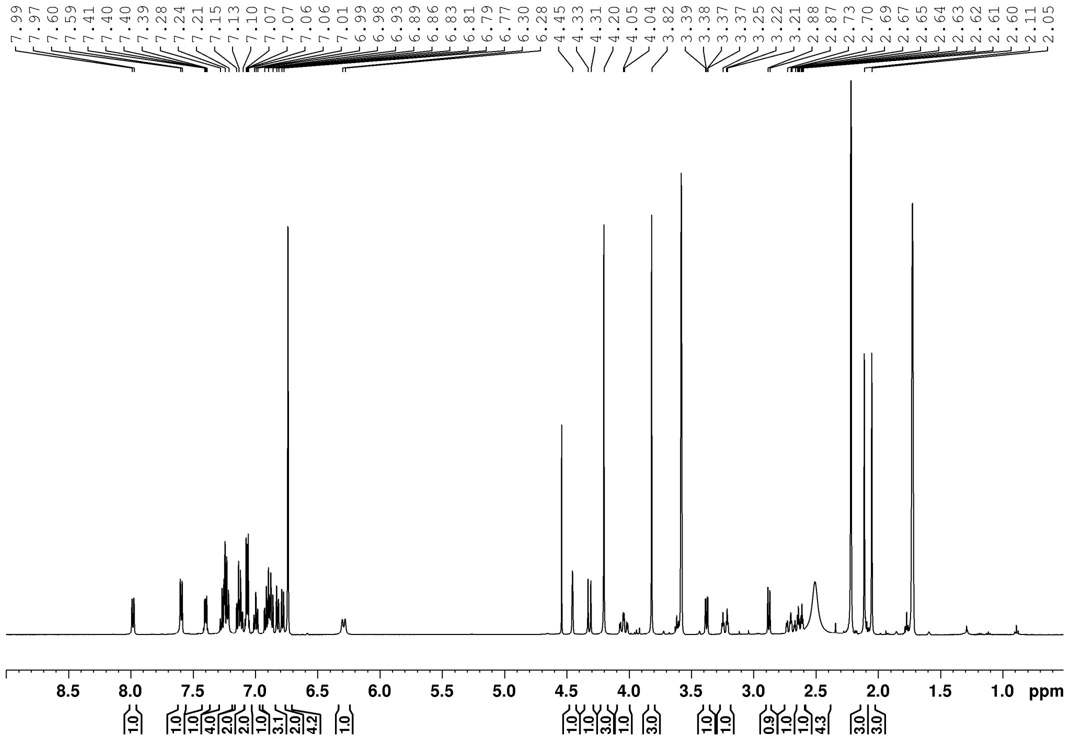

Figure S18. ^{1}H NMR (500MHz, THF-d_8, 298 K) spectrum of complex **B-CO_2**.

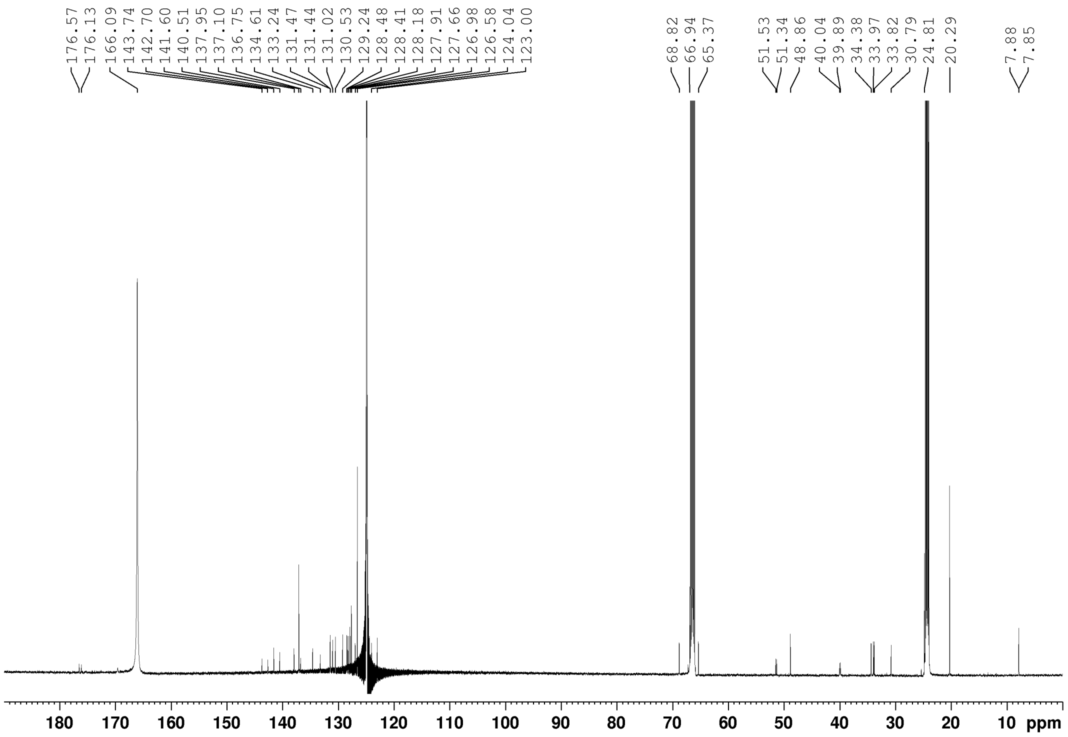

Figure S19. ^{13}C NMR (126MHz, THF-d_8, 298 K) spectrum of **B-CO₂**.

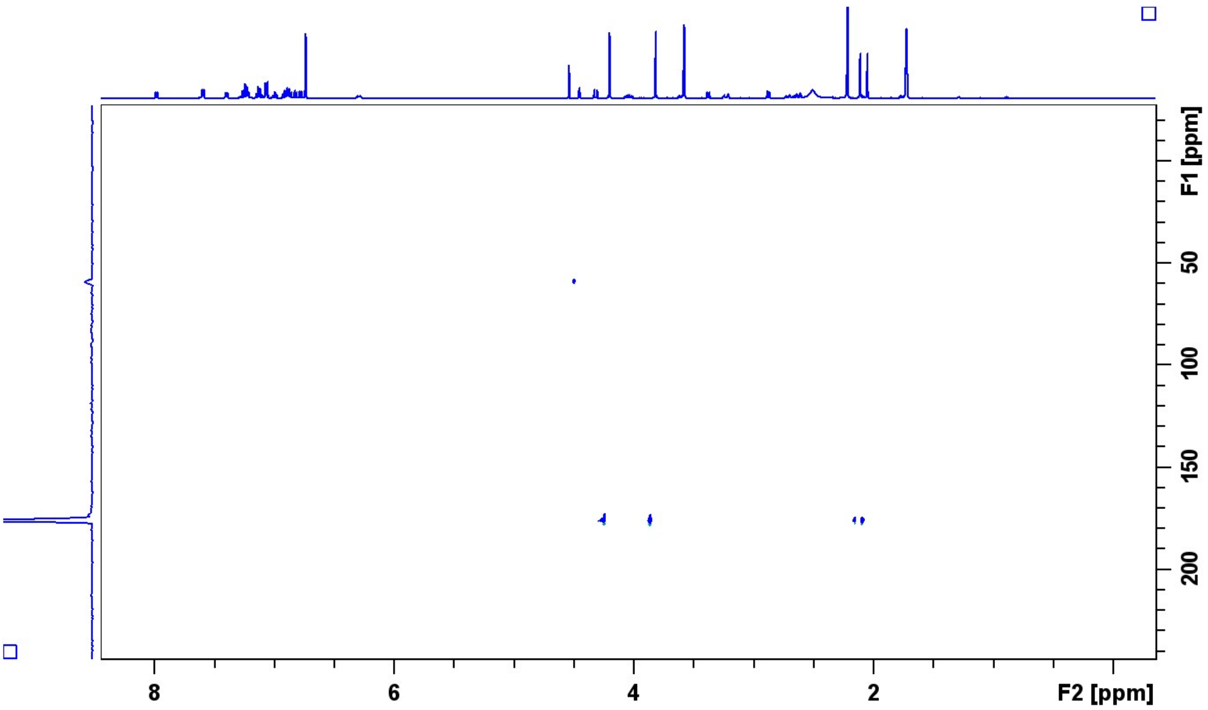

Figure S20. ^{1}H-^{15}N HSQC NMR (51MHz, THF-d_8, 298 K) spectrum of **B-CO₂**.

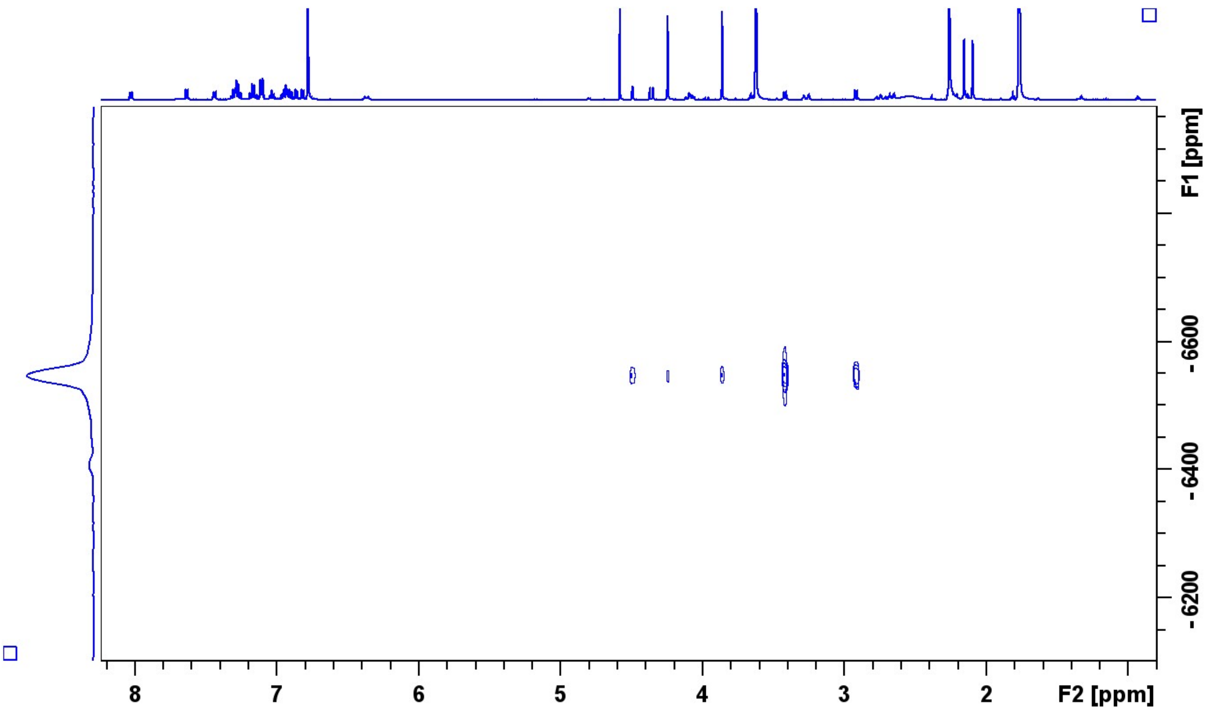

Figure S21. ^{1}H-^{103}Rh HMBC NMR (15.8MHz, THF-d_8, 298 K) spectrum of **B-CO₂**.

4. Supplementary tables of liquid NMR data

Table S1. ^{1}H, ^{15}N, ^{103}Rh and ^{13}C NMR of the bis(trop)amine ligand and complexes

Compound	δ(^{1}H) N–*H*	δ(^{15}N) *N*–H	δ(^{103}Rh)	δ(^{13}C) N–*C*–N ($J_{Rh,C}$)
**Trop$_2$NH ligand**	3.09 (exo-endo)	44.1 (exo-endo)	-	-
**(two conformers)**	4.00 (exo-exo)	-	-	-
**Complex 3**	3.97	-	−6748	-
**Complex A**	4.99	95.0	−6900	183.9 (47.1 Hz)
**Complex B**	4.77	93.0	−6868	164.2 (48.4 Hz)*
**Complex B-CH**	5.59	66.5	−7424	192.9 (63.5 Hz)
**Complex B-CO$_2$**	6.29	59.0	−6550	176.3 (55.7 Hz)

All data were measured in THF-d_8 unless marked with (*) which was measured in CD_2Cl_2. Chemical shifts (δ) expressed in ppm and (*J*) coupling constants expressed in Hz.

5. Supplementary figures of physisorption measurements

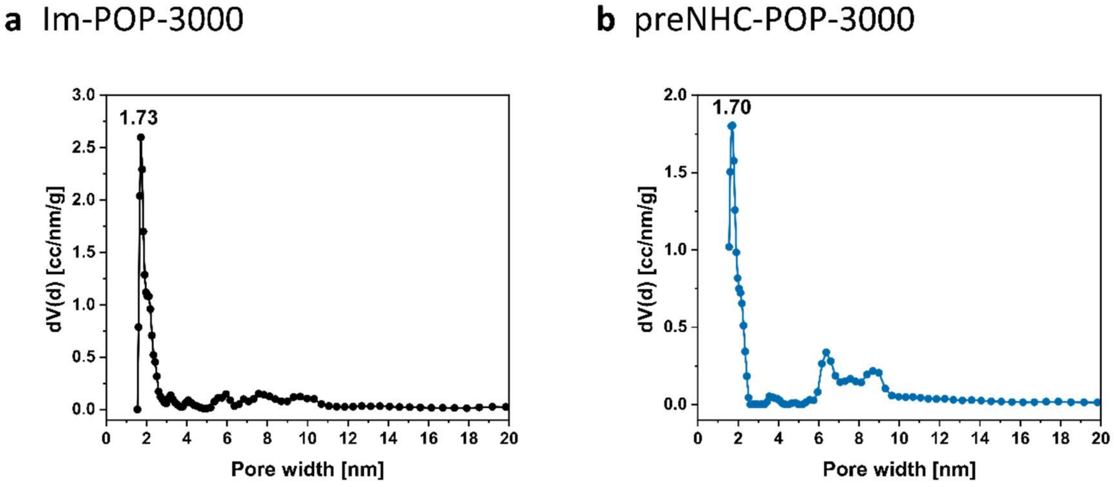

Figure S 22. Pore size distribution of (a) Im-POP-3000 and (b) preNHC-POP-3000 using the QSDFT method for N_2 at 77 K on carbon with cylindr./ sphere pores (fitting error for both: 0.14%).

6. Supplementary figures of solid-state NMR spectra

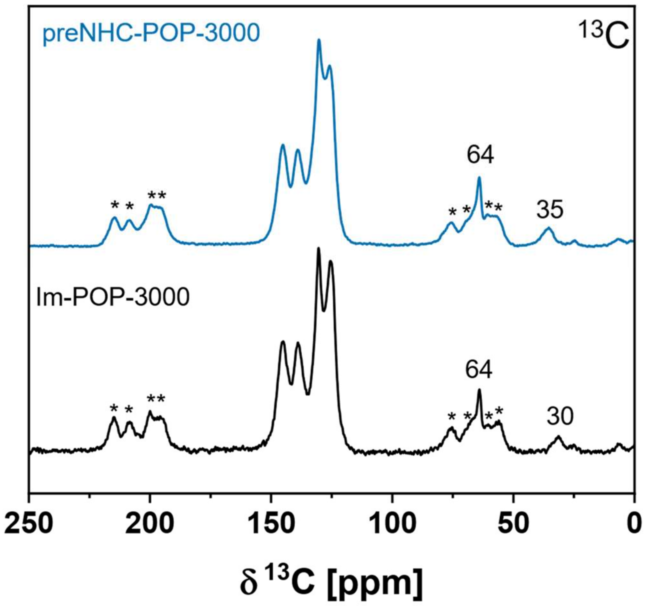

Figure S23: Stacked ^{13}C CP/MAS NMR spectra comparing **Im-POP-3000** and **preNHC-POP-3000**. Signals denoted with (*) are attributed to rotational sidebands.

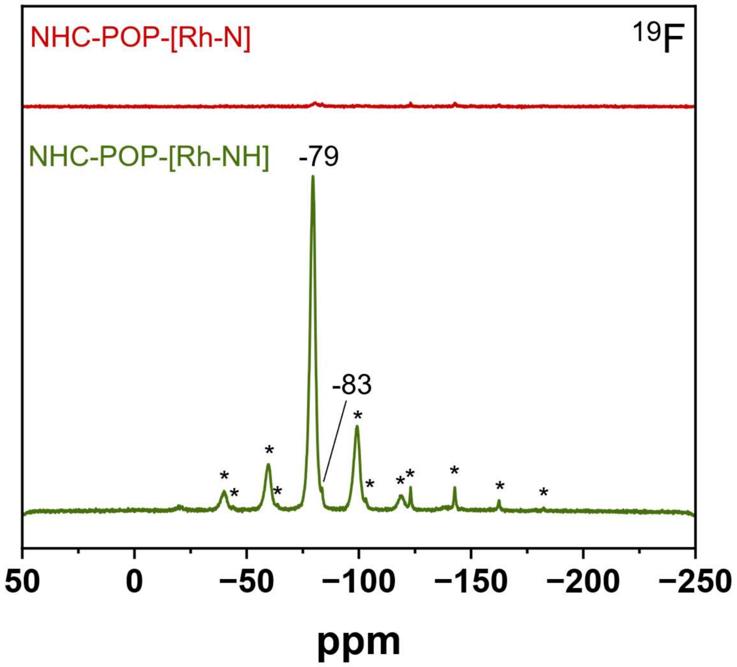

Figure S24. Stacked ^{19}F MAS NMR spectra comparing **NHC-POP-[Rh-NH]** and base activated **NHC-POP-[Rh-N]**. Signals denoted with (*) are attributed to rotational sidebands.

7. Supplementary figures of XPS spectra

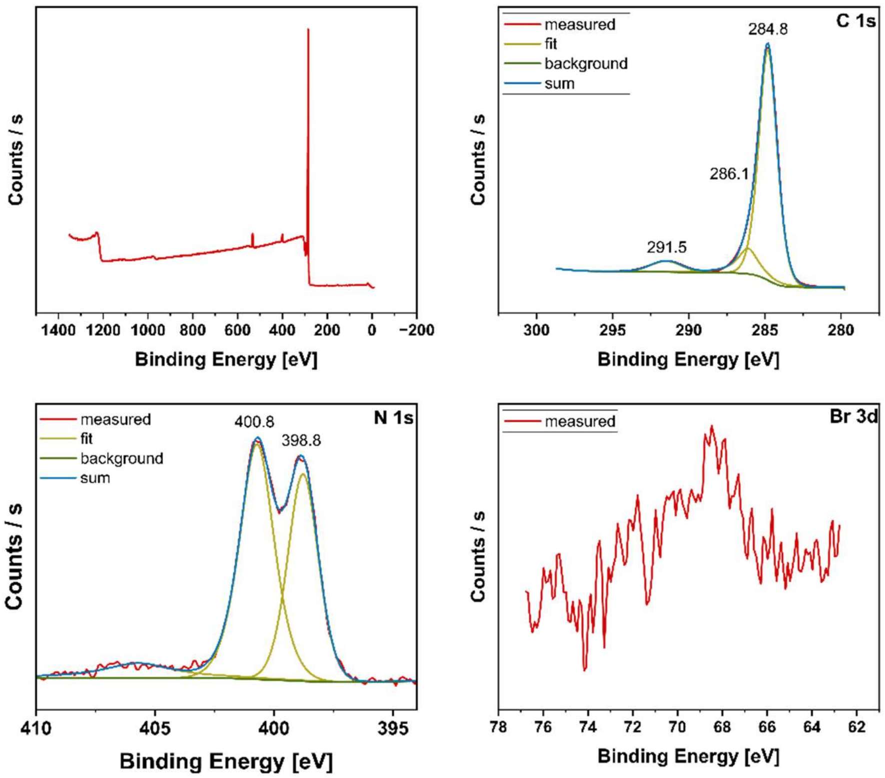

Figure S25. Survey, C 1s, N 1s and I 3d XPS spectra of **Im-POP-3000**.

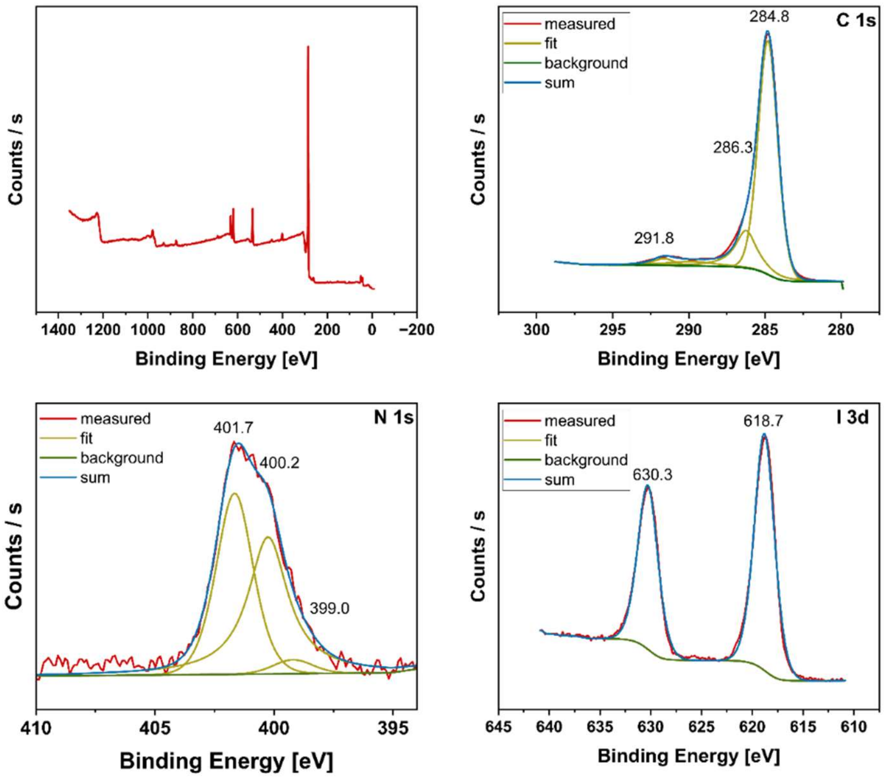

Figure S26. Survey, C 1s, N 1s and I 3d XPS spectra of **preNHC-POP-3000**.

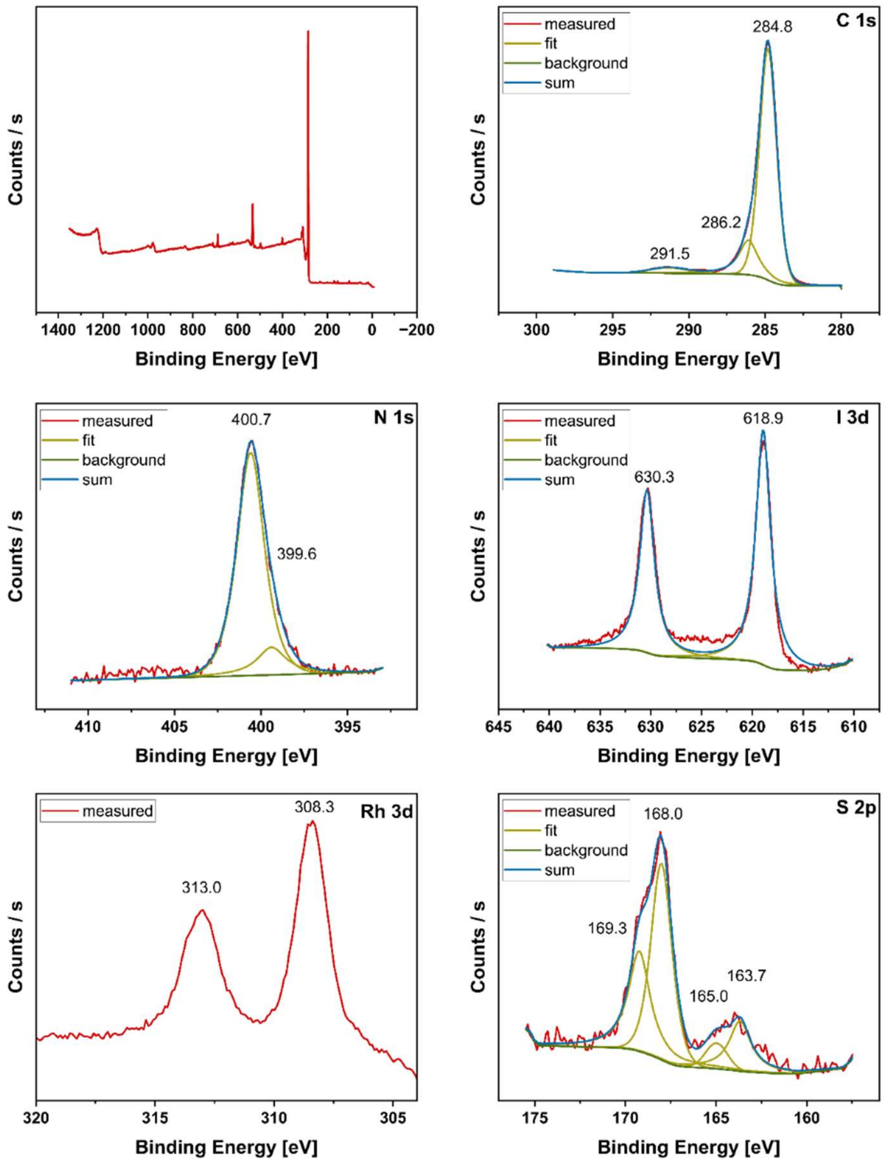

Figure S27. Survey, C 1s, N 1s and Rh 3d XPS spectra of **NHC-POP-[Rh-NH]**.

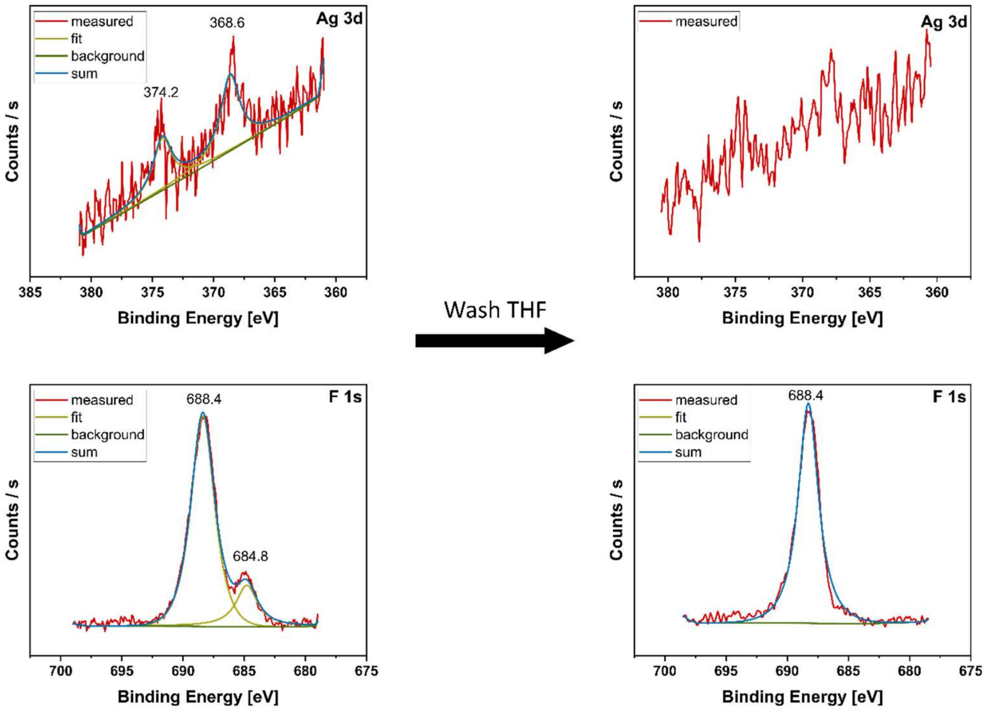

Figure S28. Ag 3d and F 1s XPS analysis of **NHC-POP-[Rh-NH]** before and after washing extensively with THF.

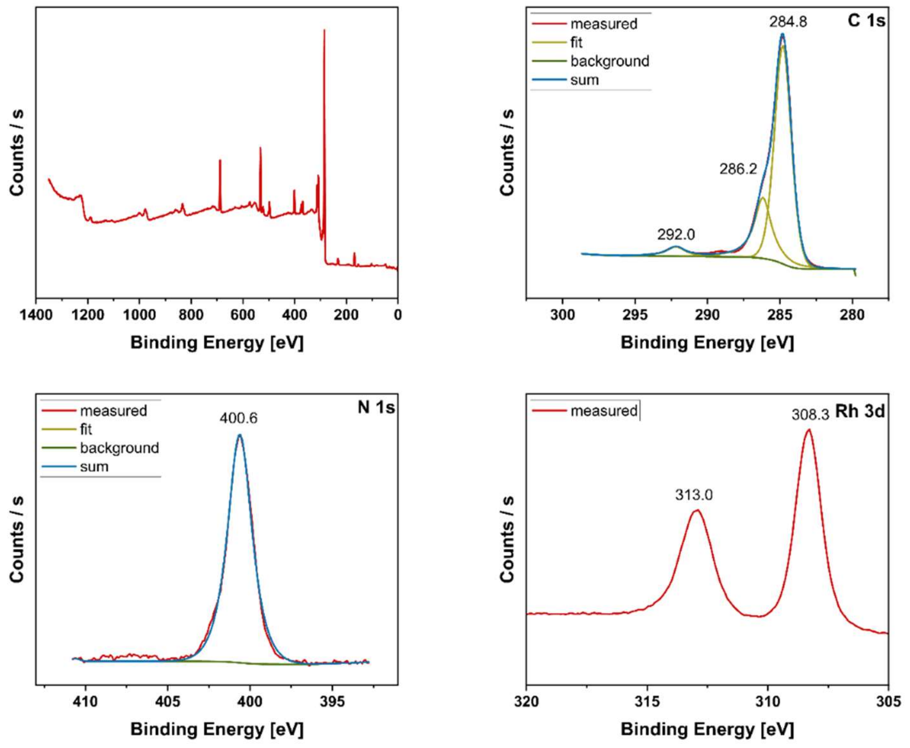

Figure S29. Survey, C 1s, N 1s and Rh 3d XPS spectra of the molecular complex [Rh(trop$_2$NH)(BzIMe)]OTf (**A**).

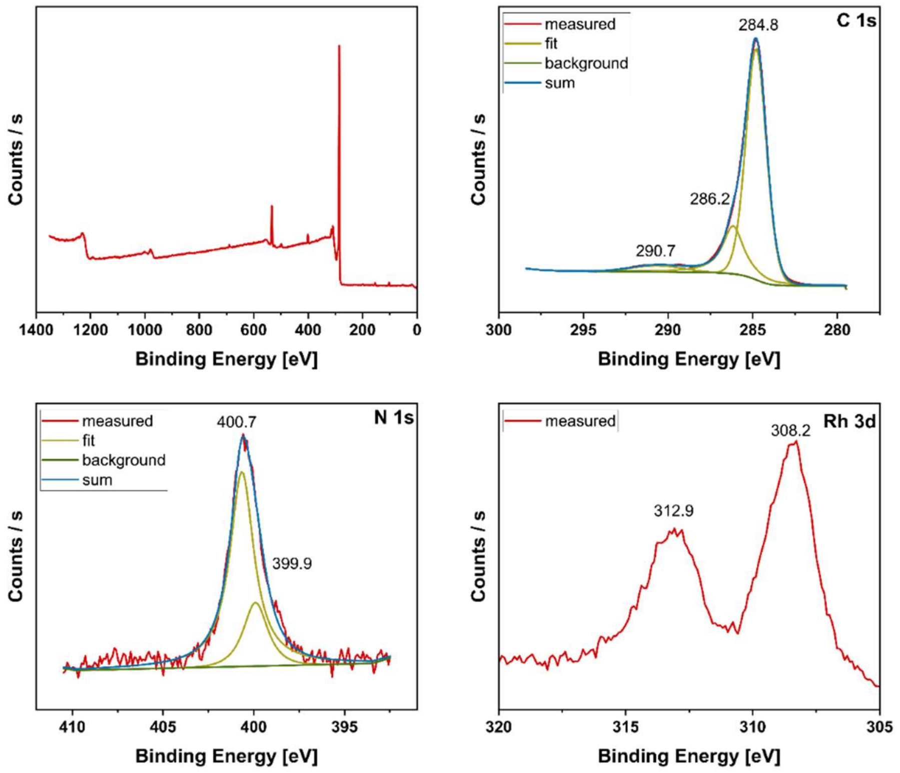

Figure S30. Survey, C 1s, N 1s and Rh 3d XPS spectra of base activated **NHC-POP-[Rh-N]**.

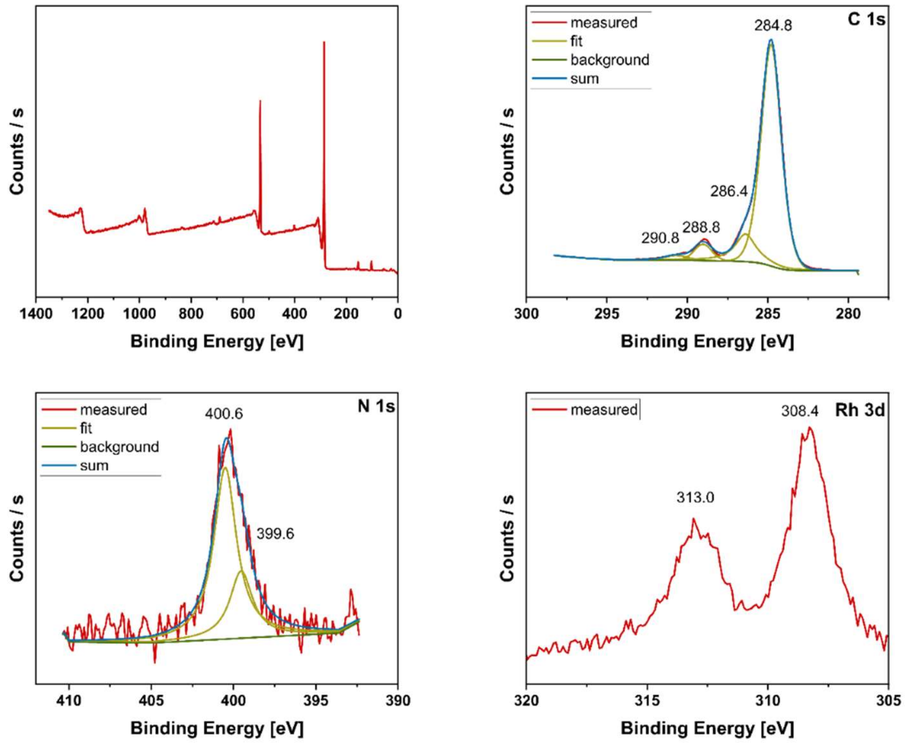

Figure S31. Survey, C 1s, N 1s and Rh 3d XPS spectra of activated **NHC-POP-[Rh(H)-N]** before catalysis in suspension.

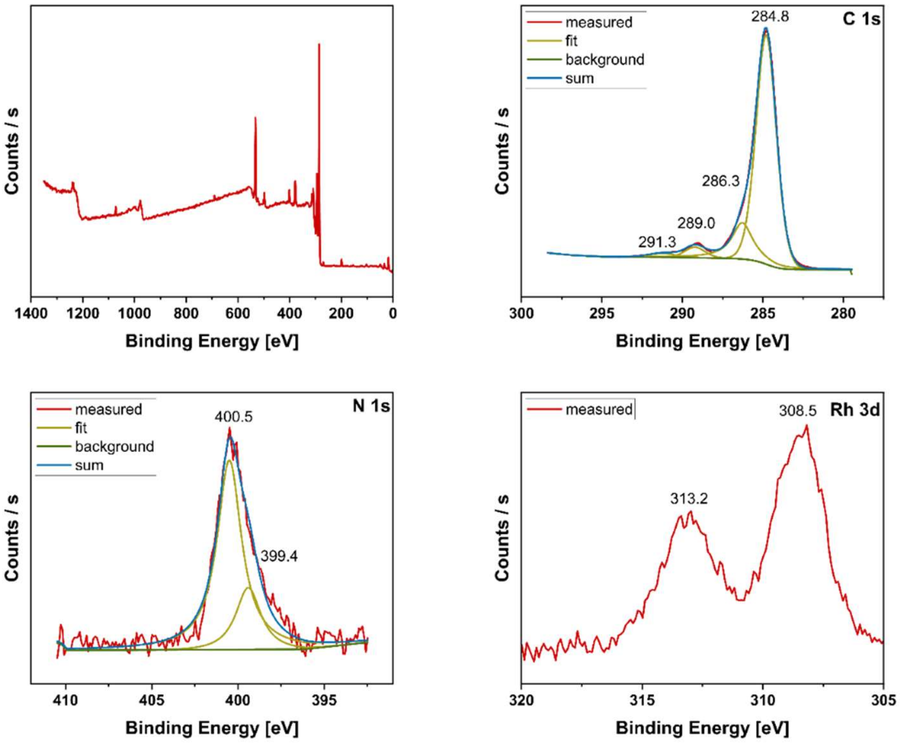

Figure S32. Survey, C 1s, N 1s and Rh 3d XPS spectra of **NHC-POP-[Rh-N]** after catalysis in suspension.

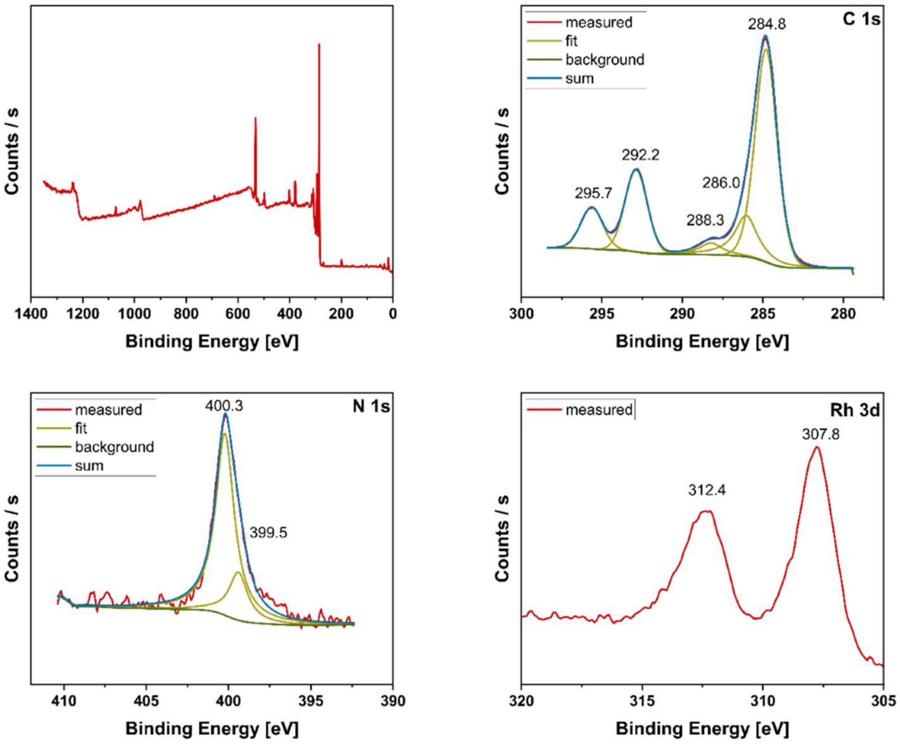

Figure S33. Survey, C 1s, N 1s and Rh 3d XPS spectra of the molecular inactivated complex (**B-CH**).

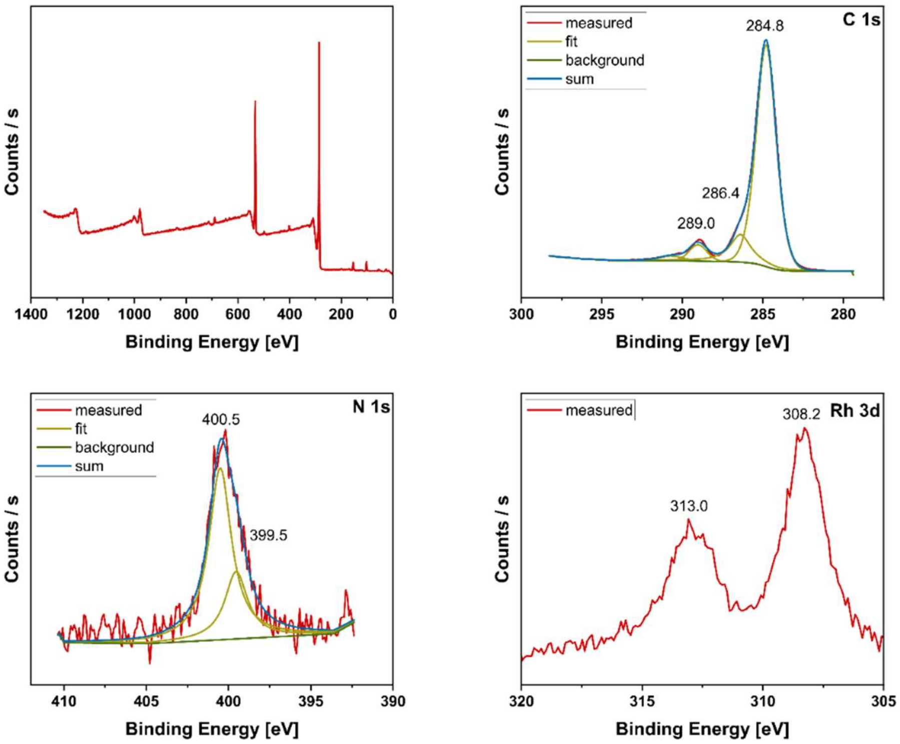

Figure S34. Survey, C 1s, N 1s and Rh 3d XPS spectra of activated **NHC-POP-[Rh(H)-NH]** before heterogeneous solid-gas catalysis.

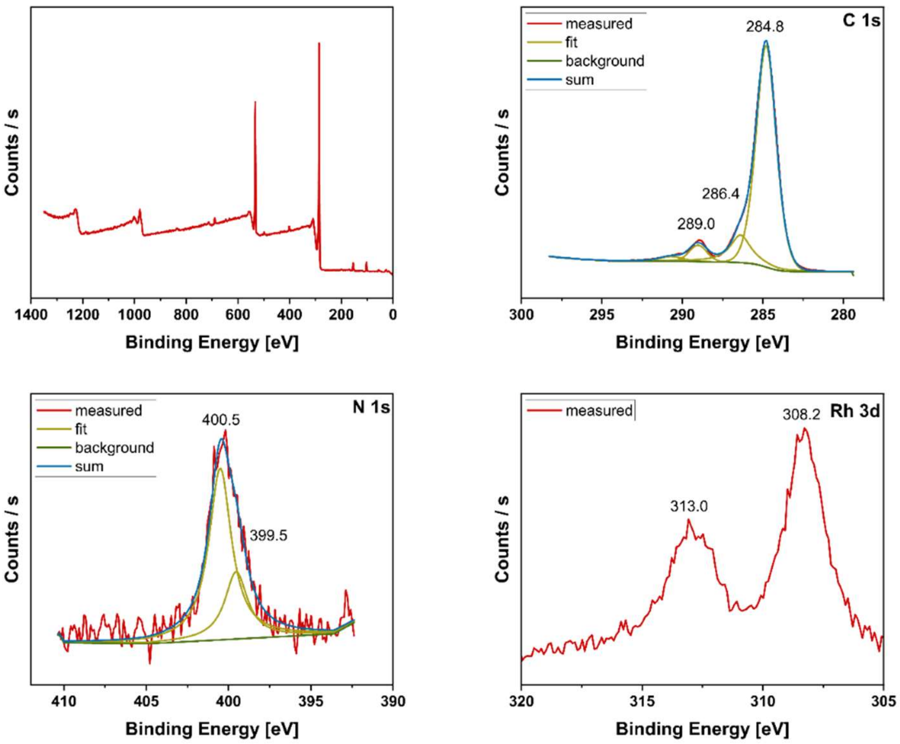

Figure S35. Survey, C 1s, N 1s and Rh 3d XPS spectra of **NHC-POP-[Rh-N]** after heterogeneous solid-gas catalysis.

8. Supplementary figures of TGA spectra

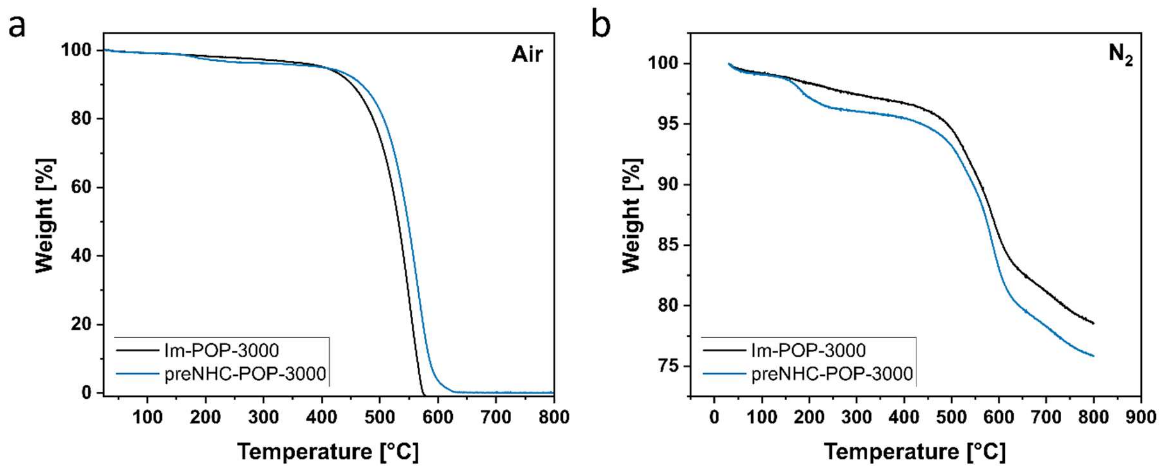

Figure S36. TGA analysis of **Im-POP-3000** and **preNHC-POP-3000** in air (a) and N_2 atmosphere (b).

9. Supplementary figures of FT-IR spectra

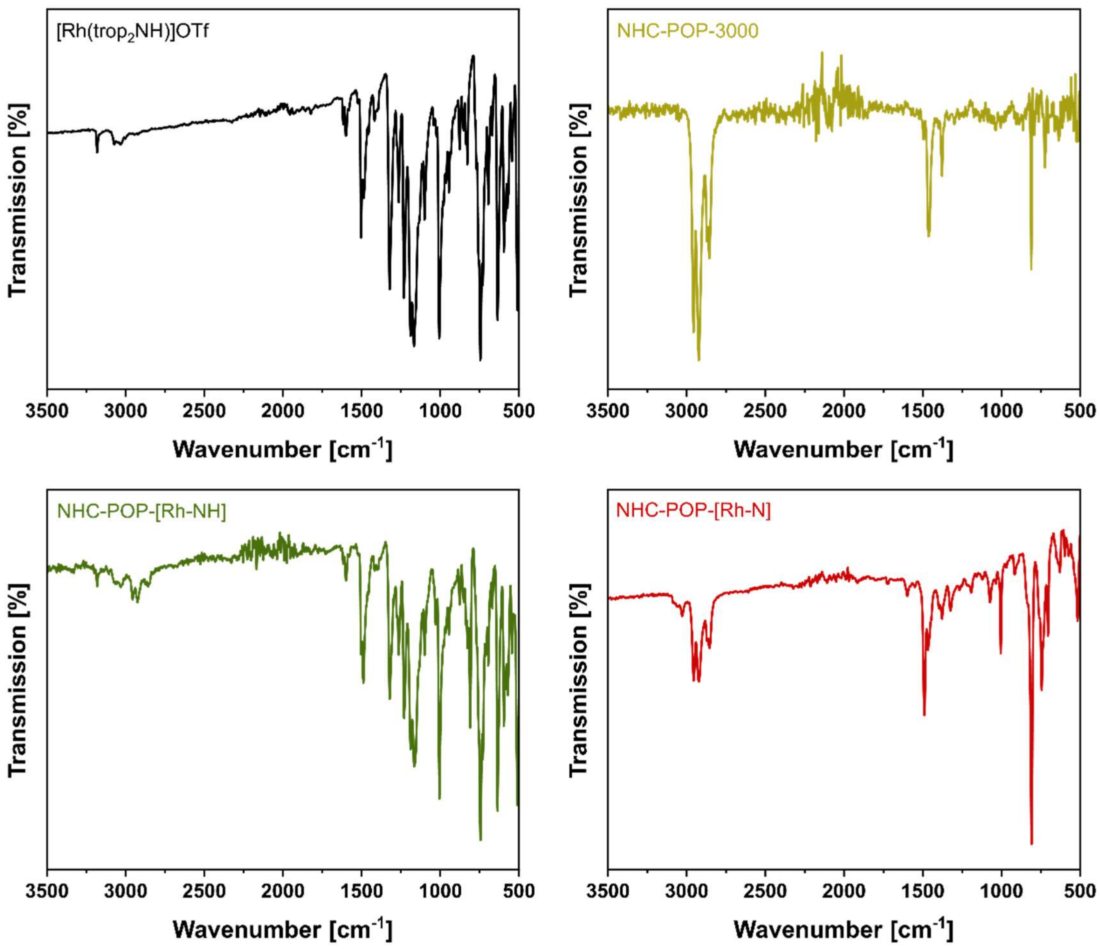

Figure S37. FT-IR spectra of molecular complex [Rh(trop$_2$NH)(THF)]OTF (**3**) (black), **NHC-POP-3000** (blue), **NHC-POP-[Rh-NH]** (green) and **NHC-POP-[Rh-N]** (red).

10. SEM-EDX Analysis

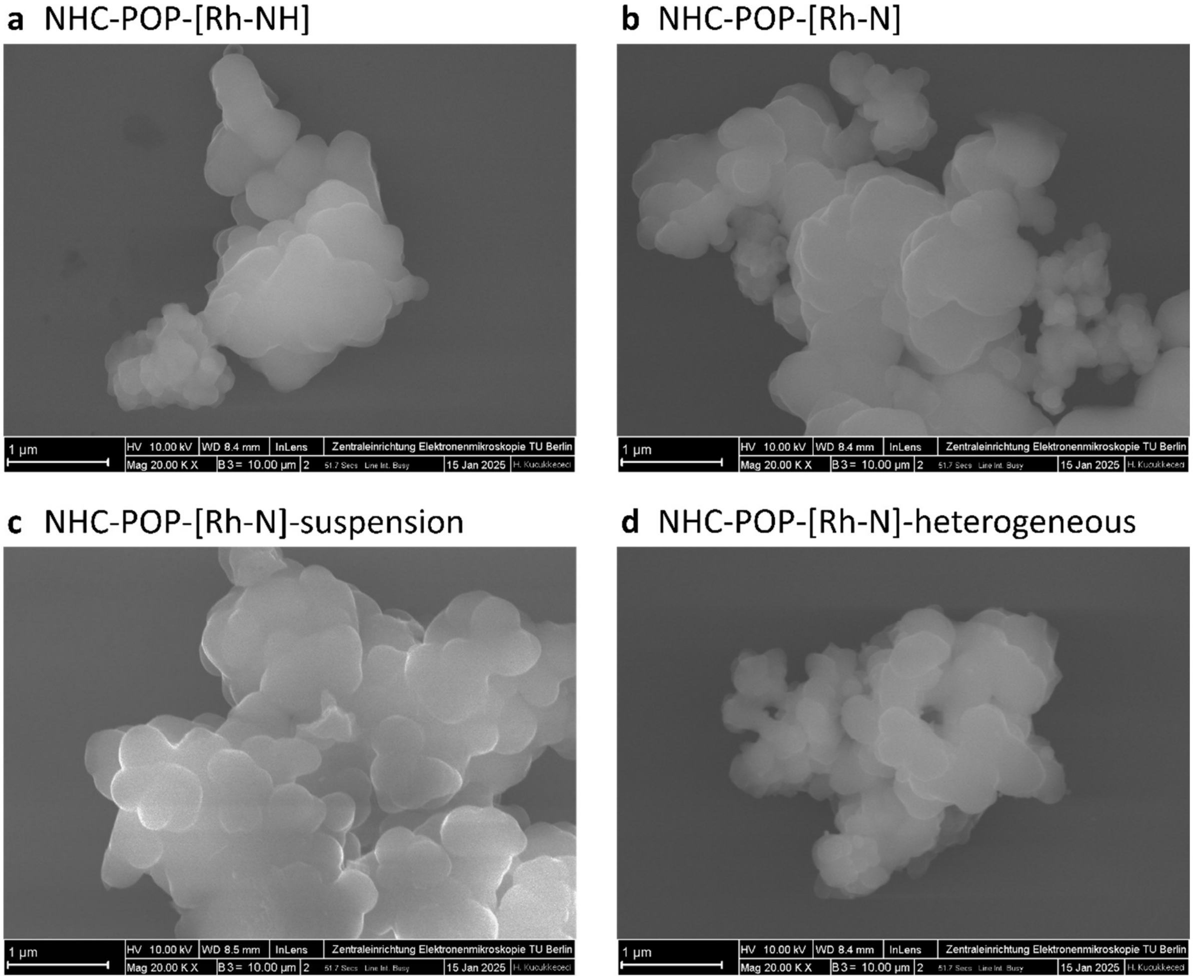

Figure S38. Morphology comparison via SEM of NHC-POP-[Rh-NH] (a), NHC-POP-[Rh-N] (b), NHC-POP-[Rh-N]- suspension after catalysis (c), NHC-POP-[Rh-N]-heterogeneous after catalysis (d).

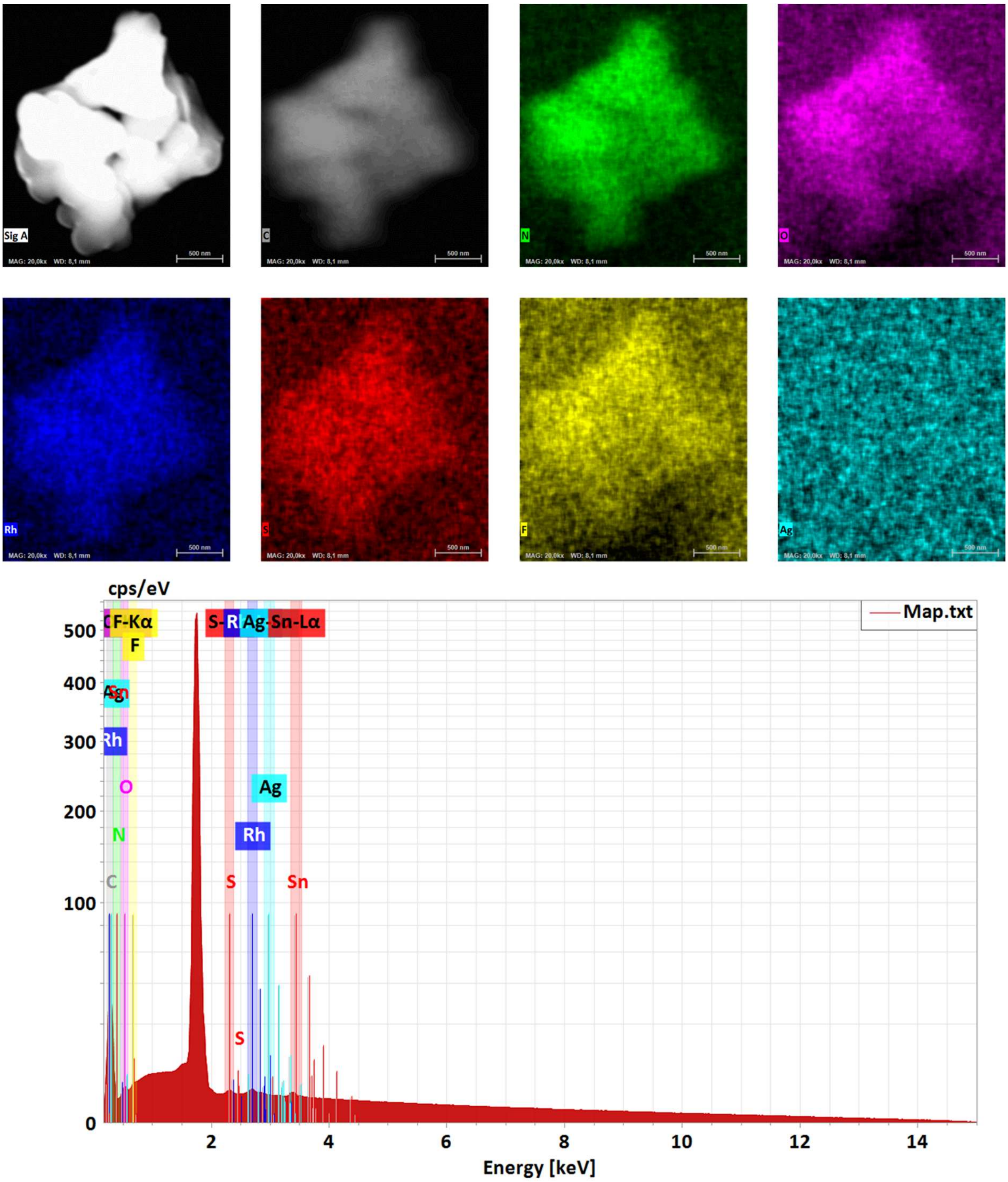

Figure S39. SEM-EDX analysis of **NHC-POP-[Rh-NH].** SEM image (grey), carbon (grey), nitrogen (green), oxygen (pink), rhodium (blue), sulfur (red), fluorine (yellow), and silver (teal, not detected).

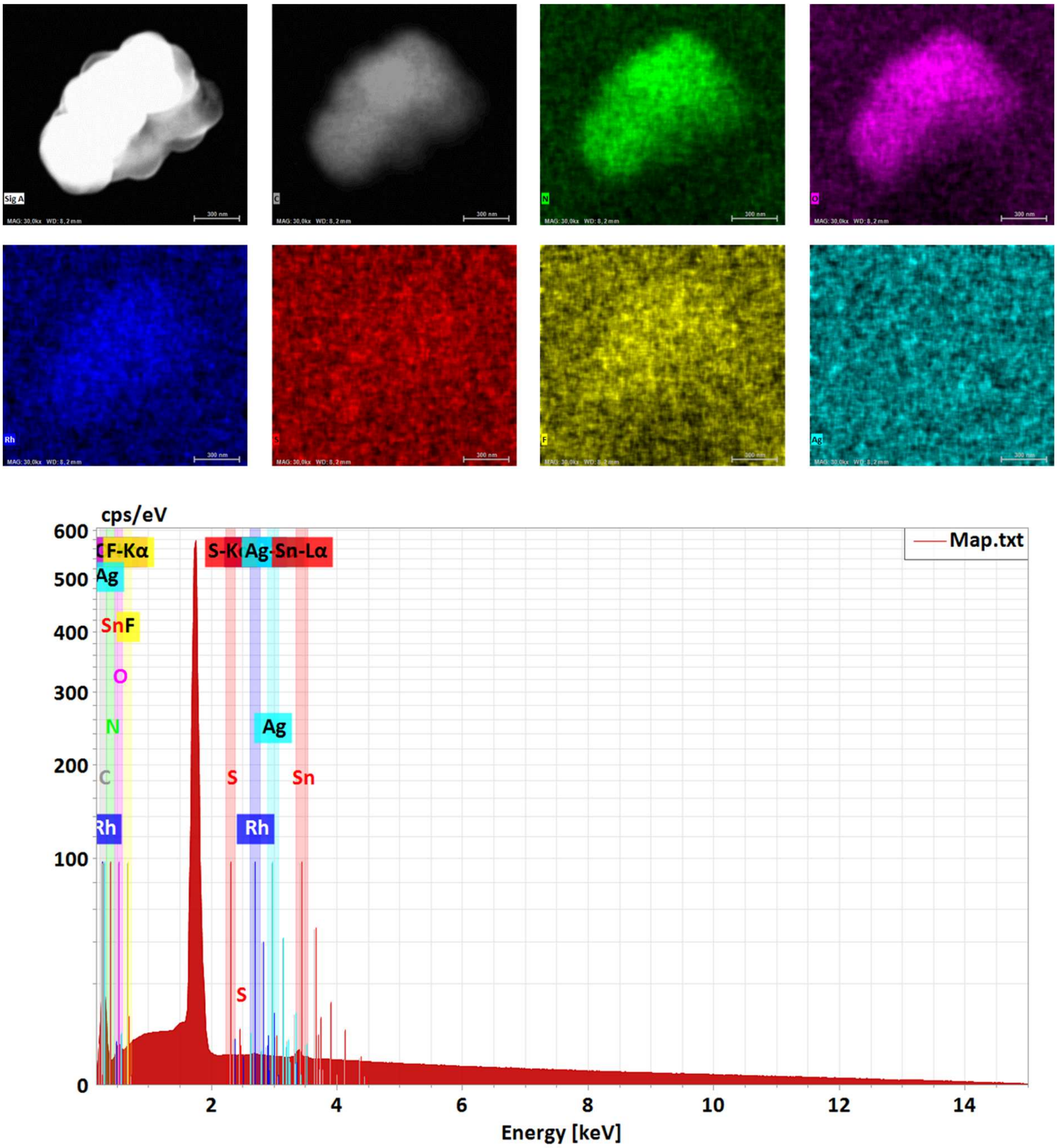

Figure S40. SEM-EDX analysis of **NHC-POP-[Rh-N]**. SEM image (grey), carbon (grey), nitrogen (green), oxygen (pink), rhodium (blue), sulfur (red, not detected), fluorine (yellow, not detected), and silver (teal, not detected).

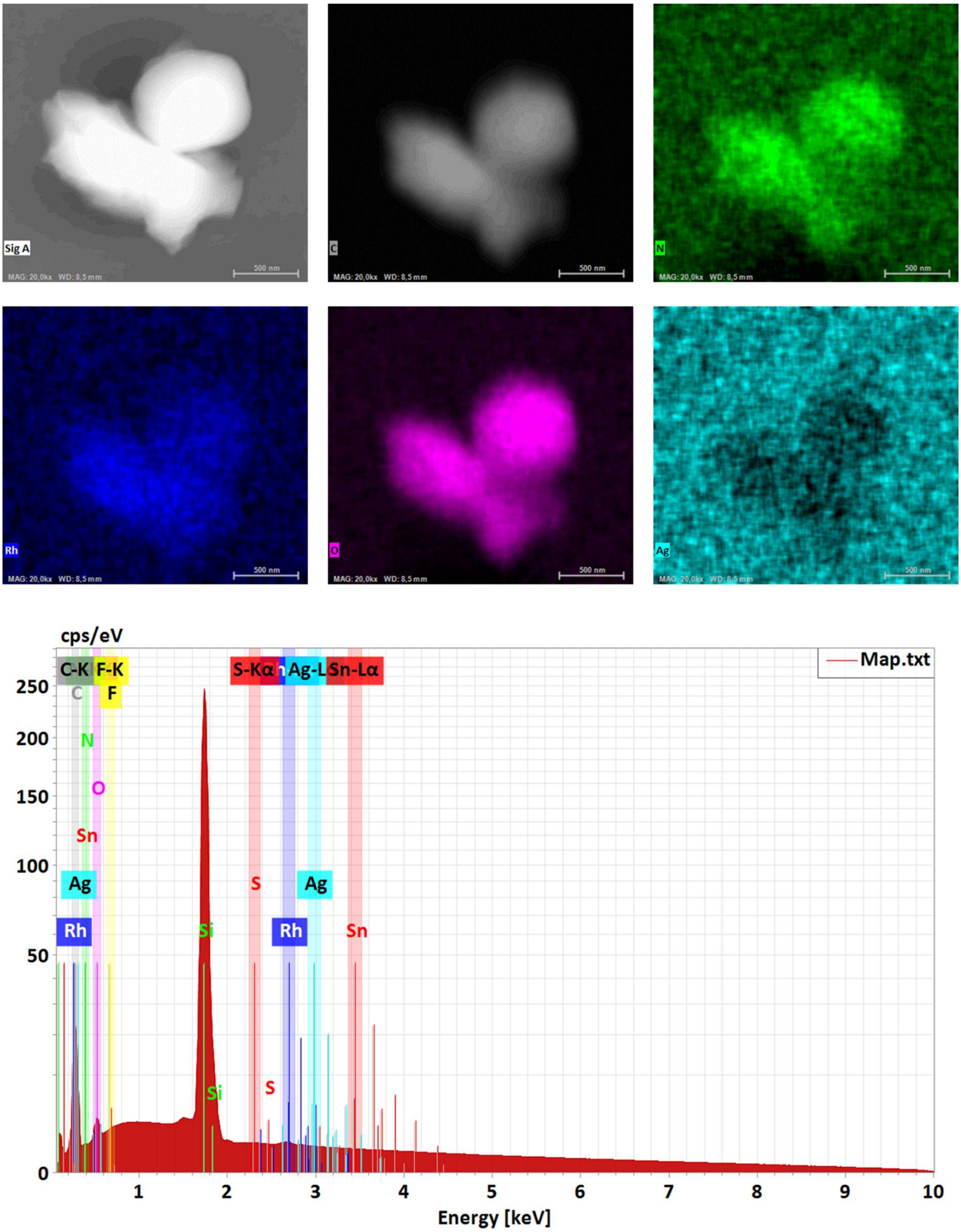

Figure S41. SEM-EDX analysis of **NHC-POP-[Rh-N]** after catalysis in suspension. SEM image (grey), carbon (grey), nitrogen (green), oxygen (pink), rhodium (blue) and silver (teal, not detected).

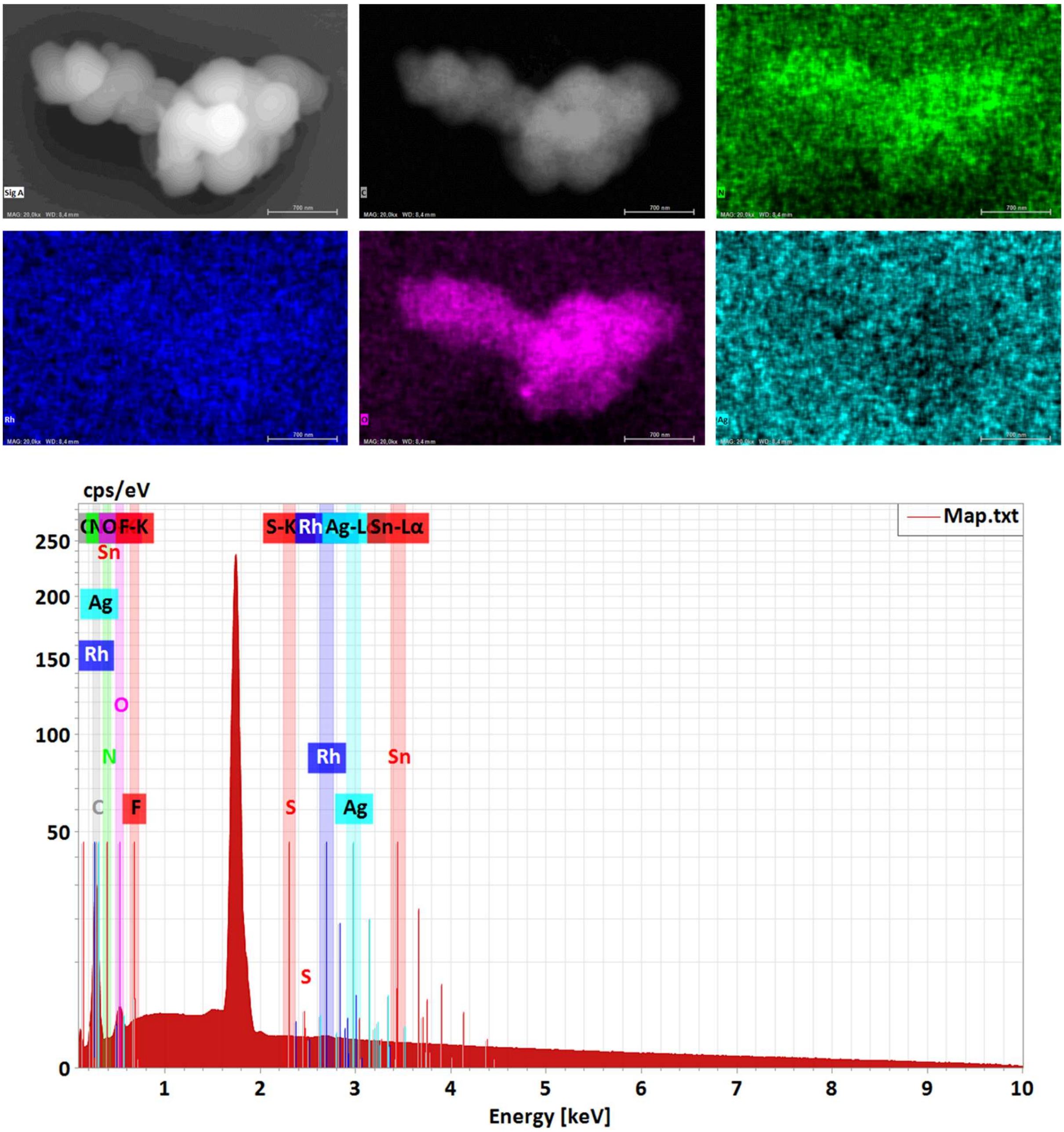

Figure S42. SEM-EDX analysis of **NHC-POP-[Rh-N]** after catalysis under heterogeneous solid-gas conditions. SEM image (grey), carbon (grey), nitrogen (green), oxygen (pink), rhodium (blue) and silver (teal, not detected).

11. Supplementary figures of ICP-OES measurements

ICP-Spectroscopy: "ULTIMA-2" Nr.: 0.0.0.0.0.

Analysis report from: 28.09.2023 **Run: Veit**

Measurement parameters
DateTime: 28.09.2023 10:07
Method: Rh-30 ppm; Power: 1000
Plasma Flow: PL1; Sheath Flow: G1; Auxiliary Flow: 0.0
Pump Speed: 20; Argon Humidificator: No
Nebulizer Flow: 0.89; Nebulizer Pressure: 2.81

Sample: SP-1 *Type:* Analysis *#:* 1 *Rack:* *Tube:* *Weight:* 0.01240 *Volume:* 50.000 *Dilution:* 1.000

Element	Line	Raw Intensity	Bkg. left	Bkg. right	Net Intensity	Solution[C]		Sample[C]	
Rh	343.489	103 907.92	1 909.51	2 099.34	101,902.36	8.42		33971.01	
		104 183.30	1 909.51	2 099.34	102,177.75	8.45		34063.86	
		107 007.26	1 909.51	2 099.34	105,001.71	8.68		35015.99	
Time	Average:	105 032.83	1 909.51	2 099.34	103 027.27	8.52	mg/l	34 350.29	mg/l
10:07	SD:	1 715.45		0.00	1 715.45	0.14	mg/l	578.38	mg/l
	RSD,%:	1.63	0.00	0.00	1.67	1.68		1.68	

Measurement parameters
DateTime: 28.09.2023 10:07
Method: Rh-30 ppm; Power: 1000
Plasma Flow: PL1; Sheath Flow: G1; Auxiliary Flow: 0.0
Pump Speed: 20; Argon Humidificator: No
Nebulizer Flow: 0.89; Nebulizer Pressure: 2.81

Review:
Analyst: Admin Date: Visa:

Figure S43. ICP-OES measurement for **NHC-POP-[Rh-NH]** (Batch 1).

ICP-Spectroscopy: "ULTIMA-2" Nr.: 0.0.0.0.0.

Analysis report from: 25.11.2024 **Run: Veit**

Measurement parameters
DateTime: 25.11.2024 10:54
Method: Rh-30 ppm; Power: 1000
Plasma Flow: PL1; Sheath Flow: G1; Auxiliary Flow: 0.0
Pump Speed: 20; Argon Humidificator: No
Nebulizer Flow: 0.87; Nebulizer Pressure: 2.82

Sample: SP-1-03 *Type:* Analysis *#:* 1 *Rack:* *Tube:* *Weight:* 0.01100 *Volume:* 50.000 *Dilution:* 1.000

Element	Line	Raw intensity	Bkg. left	Bkg. right	Net intensity	Solution[C]		Sample[C]	
Rh	343.489	62 513.70	1 632.20	1 618.61	60,888.37	5.35		24309.89	
		62 543.11	1 632.20	1 618.61	60,917.79	5.35		24321.78	
		63 395.22	1 632.20	1 618.61	61,769.89	5.43		24666.23	
Time	Average:	62 817.34	1 632.20	1 618.61	61 192.02	5.38	mg/l	24 432.63	mg/l
10:54	SD:	500.67		0.00	500.67	0.04	mg/l	202.39	mg/l
	RSD,%:	0.80	0.00	0.00	0.82	0.83		0.83	

Sample: SP-2-03 *Type:* Analysis *#:* 3 *Rack:* *Tube:* *Weight:* 0.01780 *Volume:* 50.000 *Dilution:* 1.000

Element	Line	Raw intensity	Bkg. left	Bkg. right	Net intensity	Solution[C]		Sample[C]	
Rh	343.489	97 048.07	1 856.44	1 842.04	95,198.92	8.40		23594.02	
		98 279.17	1 856.44	1 842.04	96,430.02	8.51		23901.56	
		97 279.74	1 856.44	1 842.04	95,430.58	8.42		23651.89	
Time	Average:	97 535.66	1 856.44	1 842.04	95 686.51	8.44	mg/l	23 715.82	mg/l
10:57	SD:	654.24		0.00	654.24	0.06	mg/l	163.43	mg/l
	RSD,%:	0.67	0.00	0.00	0.68	0.69		0.69	

Measurement parameters
DateTime: 25.11.2024 10:57
Method: Rh-30 ppm; Power: 1000
Plasma Flow: PL1; Sheath Flow: G1; Auxiliary Flow: 0.0
Pump Speed: 20; Argon Humidificator: No
Nebulizer Flow: 0.88; Nebulizer Pressure: 2.82

Review:
Analyst: Admin Date: Visa:

Figure S44. ICP-OES measurement for **NHC-POP-[Rh-NH]** (Batch 2) and **NHC-POP-[Rh-N]** (Batch 2).

ICP-Spectroscopy: "ULTIMA-2" Nr.: 0.0.0.0.0.

Analysis report from: 25.11.2024 **Run: Veit**

Measurement parameters

DateTime: 25.11.2024 11:32	Method:	Ag - 25 ppm	Plasma Flow:	PL1	Pump Speed:	20	Nebulizer Flow: 0.88
	Power:	1000	Sheath Flow:	G1	Argon Humidificator:	No	Nebulizer Pressure: 2.82
			Auxiliary Flow:	0.0			

Sample: SP-1-03 *Type:* Analysis *#:* 1 *Rack:* *Tube:* *Weight:* 0.01100 *Volume:* 50.000 *Dilution:* 1.000

Element	Line	Raw intensity	Bkg. left	Bkg. right	Net intensity	Solution[C]		Sample[C]	
Ag	328.068	7 420.95	18 383.48	4 308.12	- 3,674.56	0.00 X		-22.25 X	
		7 174.21	18 383.48	4 308.12	- 3,921.30	-0.01 X		-30.81 X	
		7 278.72	18 383.48	4 308.12	- 3,816.78	-0.01 X		-27.19 X	
Time	*Average:*	7 291.29	18 383.48	4 308.12	-3 804.21		mg/l		mg/l
11:32	*SD:*	123.85		0.00	123.85	0.00	mg/l	4.29	mg/l
	RSD,%:	1.70	0.00	0.00					

Sample: SP-2-03 *Type:* Analysis *#:* 3 *Rack:* *Tube:* *Weight:* 0.01780 *Volume:* 50.000 *Dilution:* 1.000

Element	Line	Raw intensity	Bkg. left	Bkg. right	Net intensity	Solution[C]		Sample[C]	
Ag	328.068	8 248.55	26 941.82	4 415.42	- 7,029.50	-0.03 X		-85.63 X	
		8 492.33	26 941.82	4 415.42	- 6,785.72	-0.03 X		-80.41 X	
		8 330.95	26 941.82	4 415.42	- 6,947.09	-0.03 X		-83.86 X	
Time	*Average:*	8 357.28	26 941.82	4 415.42	-6 920.77		mg/l		mg/l
11:34	*SD:*	124.00			124.00	0.00	mg/l	2.66	mg/l
	RSD,%:	1.48	0.00	0.00					

Measurement parameters

DateTime: 25.11.2024 11:34	Method:	Ag - 25 ppm	Plasma Flow:	PL1	Pump Speed:	20	Nebulizer Flow: 0.87
	Power:	1000	Sheath Flow:	G1	Argon Humidificator:	No	Nebulizer Pressure: 2.82
			Auxiliary Flow:	0.0			

Review:

Analyst: Admin Date: Visa:

Figure S45. ICP-OES measurement of Ag for **NHC-POP-[Rh-NH]** (Batch 2) and **NHC-POP-[Rh-N]** (Batch 2).

ICP-Spectroscopy: "ULTIMA-2" Nr.: 0.0.0.0.0.

Analysis report from: 22.01.2025 **Run: Veit**

Measurement parameters

DateTime: 22.01.2025 11:00	Method: Rh-30 ppm	Plasma Flow:	PL1	Pump Speed:	20	Nebulizer Flow:	0.91
	Power: 1000	Sheath Flow:	G1	Argon Humidificator:	No	Nebulizer Pressure:	2.83
		Auxiliary Flow:	0.0				

Sample: 4.1-SC156 *Type:* Analysis *#:* 1 *Rack:* *Tube:* *Weight:* 0.02110 *Volume:* 50.000 *Dilution:* 1.000

Element	Line	Raw intensity	Bkg. left	Bkg. right	Net intensity	Solution[C]		Sample[C]	
Rh	343.489	105 401.14	2 009.51	1 457.03	103,671.16	9.57		22675.73	
		107 375.15	2 009.51	1 457.03	105,645.17	9.75		23115.74	
		106 714.98	2 009.51	1 457.03	104,985.00	9.69		22968.59	
Time	*Average:*	106 497.09	2 009.51	1 457.03	104 767.11	9.67	mg/l	22 920.02	mg/l
11:00	*SD:*	1 004.88		0.00	1 004.88	0.09	mg/l	223.99	mg/l
	RSD,%:	0.94	0.00	0.00	0.96	0.98		0.98	

Sample: 5.1-SC160 *Type:* Analysis *#:* 3 *Rack:* *Tube:* *Weight:* 0.01150 *Volume:* 50.000 *Dilution:* 1.000

Element	Line	Raw intensity	Bkg. left	Bkg. right	Net intensity	Solution[C]		Sample[C]	
Rh	343.489	56 996.37	1 490.27	1 312.76	55,595.91	5.05		21943.66	
		57 428.63	1 490.27	1 312.76	56,028.18	5.09		22120.44	
		57 308.74	1 490.27	1 312.76	55,908.28	5.08		22071.41	
Time	*Average:*	57 244.58	1 490.27	1 312.76	55 844.12	5.07	mg/l	22 045.17	mg/l
11:02	*SD:*	223.16	0.00		223.16	0.02	mg/l	91.27	mg/l
	RSD,%:	0.39	0.00	0.00	0.40	0.41		0.41	

Measurement parameters

DateTime: 22.01.2025 11:02	Method: Rh-30 ppm	Plasma Flow:	PL1	Pump Speed:	20	Nebulizer Flow:	0.90
	Power: 1000	Sheath Flow:	G1	Argon Humidificator:	No	Nebulizer Pressure:	2.83
		Auxiliary Flow:	0.0				

Review:

Analyst: Admin Date: Visa:

Figure S46. ICP-OES measurement of Rh after solid-liquid-gas catalysis (top) and after solid-gas catalysis (bottom).

12. Supplementary figures of HAADF-STEM EDX Analysis

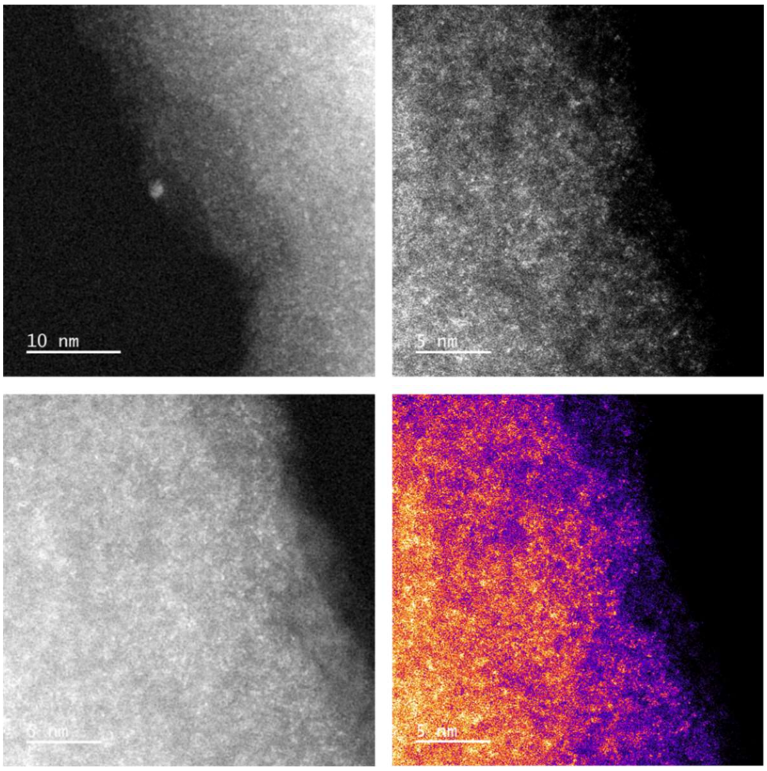

Figure S47. HAADF –STEM images of NHC-POP-[Rh-NH]. Images were denoised using non-linear filter: H. Du, A nonlinear filtering algorithm for denoising HR(S)TEM micrographs, Ultramicroscopy 151 (2015) 62–67. doi: 10.1016/j.ultramic.2014.11.012.

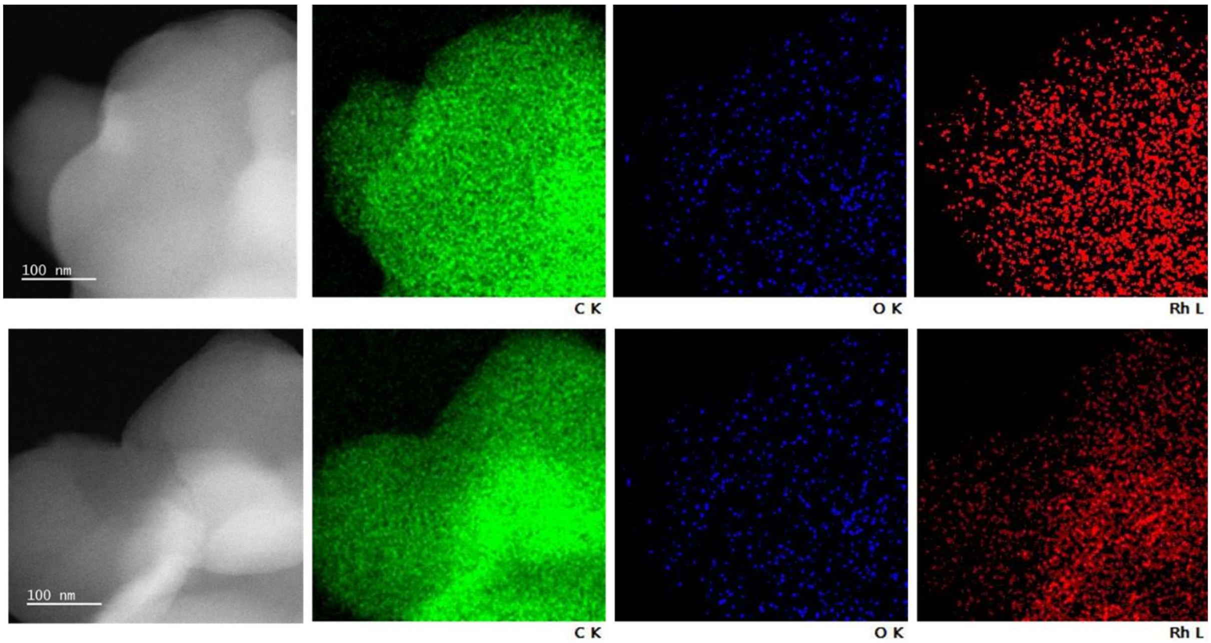

Figure S48. EDX elemental mapping of **NHC-POP-[Rh-NH],** carbon (green), oxygen (blue), rhodium (red).

13. Supplementary notes of crystallographic data

13.1 Supplementary notes of crystal structure information

The X-ray crystallographic coordinates for structures reported in this article have been deposited at the Cambridge Crystallographic Data Centre (CCDC), under deposition number CCDC- 2418688 **(B-CH)** and 2418689 (**B-CO_2**). These data can be obtained free of charge from the 'Cambridge Crystallographic Data Centre' via www.ccdc.cam.ac.uk/data_request/cif.

13.2 Supplementary tables and figures of structural data

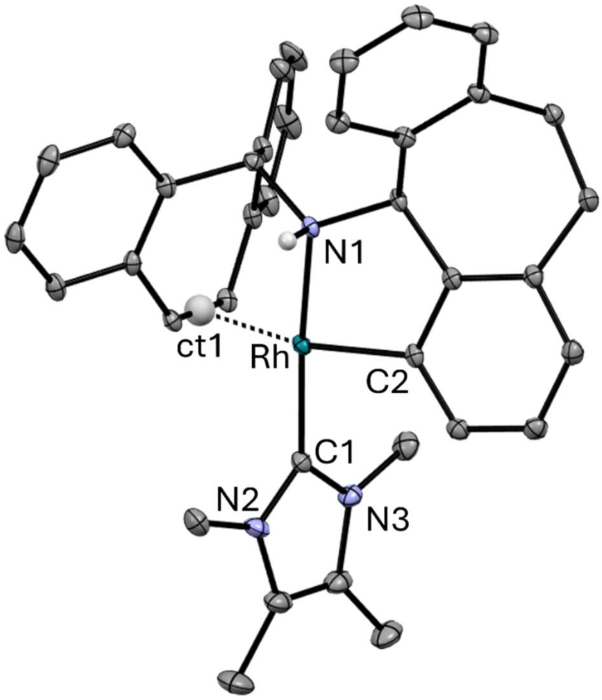

Figure S49. Single-crystal X-ray diffraction structures of Rh complex **B-CH**. The unit cell contains two independent molecules and only one is shown here for clarity. Thermal ellipsoids are drawn at 50% probability; nonbonding solvents, and hydrogen atoms apart from N–*H* are omitted for clarity. Selected bond lengths (Å) and angles (°). Rh–N1 2.102(2), Rh-C1 1.987(3), Rh-C2 2.045(2), Rh–ct1 2.026, ct1–Rh–C2 167.87, N1-Rh–C1 172.37(9), N1–Rh–C2 79.47(8), N2–C1–N3 103.7(2).

Short structural comment/discussion:

Single crystals of complex **B-CH** were grown from a hexane solution, allowing its molecular structure to be determined by single-crystal X-ray diffraction. The complex exhibits a distorted square planar geometry, with an N1–Rh–C1 angle of 172.4°, and an angle between the olefin and the anionic bonded carbon on the trop-arene (C2, ct1) of 167.9°. Notably, one of the trop units is rotated by 90° and bonded to rhodium *via* the trop-arene axial carbon C2 [Rh–C2 = 2.045(2) Å], which is significantly longer than the axial Rh–C1 bond to the NHC ligand [1.987(3) Å]. The olefin bond of the C–H inserted trop unit has been hydrogenated to an alkane group, as evidenced by the longer bond length of C7–C8 [1.526(4) Å] compared to the coordinated olefin bond C=C of ct1 on the trop unit *trans* to C2 [1.418(3) Å]. The formation of an alkane is also reflected in the large C6–C7–C8–C9 torsional angle of 64°. The Rh–N bond length is 2.102(2) Å, which is slightly shorter than in the cationic complex **B** and previously reported complexes [2.13–2.15 Å].

Table S2. Crystal data and structure refinement for **B-CH**

Identification code	SK1_RhH_dec_1_auto
Empirical formula	$C_{40}H_{43}N_3Rh$
Formula weight	668.68
Temperature/K	100.15
Crystal system	triclinic
Space group	P-1
a/Å	13.2554(2)
b/Å	14.24300(10)
c/Å	18.3871(2)
α/°	94.5850(10)
β/°	95.6840(10)
γ/°	106.8150(10)
Volume/Å^3	3285.10(7)
Z	4
ρ_{calc}g/cm^3	1.352
μ/mm^{-1}	4.437
F(000)	1396.0
Crystal size/mm^3	0.118 × 0.089 × 0.0415
Radiation	CuKα (λ = 1.54184)
2Θ range for data collection/°	4.862 to 160.792
Index ranges	-16 ≤ h ≤ 16, -18 ≤ k ≤ 17, -23 ≤ l ≤ 23
Reflections collected	82603
Independent reflections	14266 [R_{int} = 0.0574, R_{sigma} = 0.0377]
Data/restraints/parameters	14266/0/811
Goodness-of-fit on F^2	1.139
Final R indexes [I>=2σ (I)]	R_1 = 0.0368, wR_2 = 0.1022
Final R indexes [all data]	R_1 = 0.0384, wR_2 = 0.1034
Largest diff. peak/hole / e Å^{-3}	1.00/-1.18

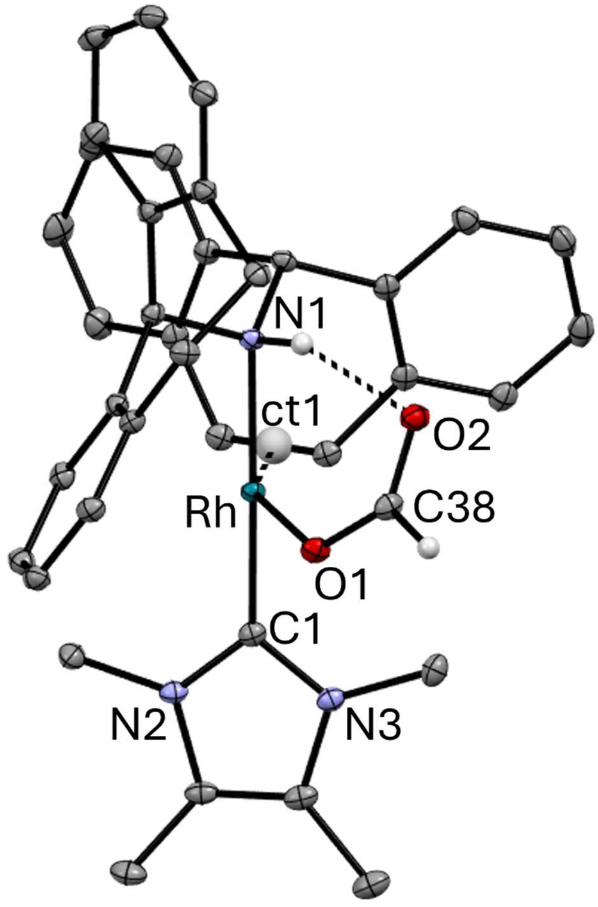

Figure S50. Single-crystal X-ray diffraction structures of Rh complex **B-CO2**. Thermal ellipsoids are drawn at 50% probability; nonbonding solvents, triflate molecules, and hydrogen atoms apart from N–*H* and O–*H* are omitted for clarity. Selected bond lengths (Å) and angles (°). Rh–N1 2.158(2), Rh–O1 2.175(1) , Rh-C1 1.983(2), Rh–ct1 1.944, C38-O1 1.265(2), C38-O2 1.237(2), NH1-O2 2.06(3), ct1–Rh–O1 149.1, N1–Rh–O1 92.44(5), N1-Rh–C1 176.84(7), N2–C1–N3 104.1(1).

Short structural comment/discussion:

Single crystals of complex **B-CO_2** were grown from hexane, allowing its molecular structure determination by single-crystal X-ray diffraction. The complex adopts a 'saw-horse' structure (trigonal bipyramidal geometry missing one corner of the trigonal plane), with an N1–Rh–C1 angle of 176.8°, and an angle between the olefin and the formate-bound oxygen (ct1, O1) of 149.1°. The formate is activated at rhodium and coordinated by a relatively weak bond [Rh–O1 = 2.175(1) Å], but is stabilized by an intramolecular hydrogen bond with the trop N-H proton [N–H···O2 = 2.06(3) Å], forming a pseudo-6-membered metallacycle that likely contributes to the complex's stability. Rotational freedom of the formate [Rh–O1–C38–O2 = 24.5(3)°] relieves steric strain and enlarges the N1–Rh–O1 angle of 92.44(5)°, which relaxes the structure. Notably, one of the trop units is rotated by 90° and the olefin bond has been hydrogenated to an alkane group, as evidenced by the longer bond length of C7–C8 [1.522(3) Å] compared to the coordinated olefin bond C=C of ct1 on the trop unit *trans* to O1 [1.451(2) Å]. The formation of an alkane is also reflected in the large C6–C7–C8–C9 torsional angle of 70.5(2)°. The Rh–N bond length is 2.158(2) Å, which is slightly longer than in the cationic complex **B** and previously reported complexes [2.13–2.15 Å].

Table S3. Crystal data and structure refinement for **B-CO_2**

Identification code	S114_1_auto
Empirical formula	C38H38N3O2Rh
Formula weight	671.62
Temperature/K	99.98(10)
Crystal system	monoclinic
Space group	P21/c
a/Å	12.59057(7)
b/Å	12.65567(7)
c/Å	19.13229(11)
α/°	90
β/°	90.5955(5)
γ/°	90
Volume/Å3	3048.42(3)
Z	4
ρcalcg/cm3	1.463
μ/mm-1	4.838
F(000)	1392.0
Crystal size/mm3	0.5 × 0.2 × 0.2
Radiation	Cu Kα (λ = 1.54184)
2Θ range for data collection/°	7.022 to 156.89
Index ranges	-16 ≤ h ≤ 14, -15 ≤ k ≤ 16, -24 ≤ l ≤ 24
Reflections collected	44974
Independent reflections	6528 [Rint = 0.0335, Rsigma = 0.0222]
Data/restraints/parameters	6528/0/405
Goodness-of-fit on F2	1.041
Final R indexes [I>=2σ (I)]	R1 = 0.0262, wR2 = 0.0646
Final R indexes [all data]	R1 = 0.0271, wR2 = 0.0651
Largest diff. peak/hole / e Å-3	1.61/-0.71

14. Supplementary notes of catalytic investigations

14.1 Supplementary notes of the reaction setup

***Attention The reaction mixture of N_2O and H_2 is explosive. Rigorous safety measurements have to be applied*.**

All the equipment was bought from Swagelok and Ace Glass. The kinetic experiments were conducted in Ace pressure tubes (35mL, #15) equipped with an adapter (Adapter, #15 Ace-Thred™ to 1/4in FNPT,3/8in drill-thru,PTFE) and a CAPFE O-ring (size 110). The reactor was fitted to stainless steel tubing (1/4 in.) with a Swagelok Tube Fitting (Male Connector, 1/4 in. Tube OD x 1/4 in. Male NPT) equipped with an Ultra-torr GC-septa connection, valve (valve a), pressure gauge (range 0-6 bar, Part number: PGI-50M-BG6-LAOX) and a second dosing valve (valve b), further to a branch tee connected to a N_2O (purity N5.0) and H_2-gas supply and a vacuum pump.

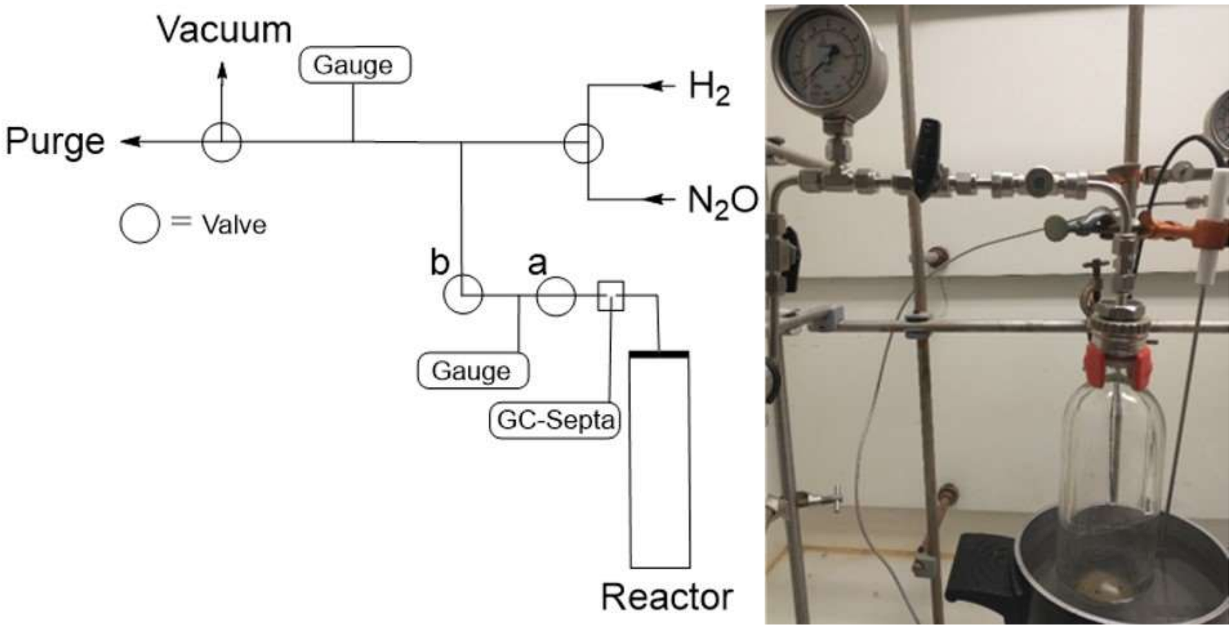

Figure S51. A Scheme of the reactor setup on the left and the actual setup on the right, this setup was used in all catalysis reactions; in the kinetic investigations and for dosing of gases in the catalytic investigations.

14.2 Supplementary notes of the of the general procedure of the N_2O hydrogenation reaction in suspension

Inside a glovebox, a screw-cap Schlenk flask (Normag, 130 mL) was charged with catalyst **NHC-POP-[Rh-N]** (1.0 mg, 2.4–3.4 wt% [Rh], 0.00023-0.00033 mmol [Rh], 20–30 ppm [Rh]), THF or water (5.0 mL, 0.05–0.07 mM [Rh]), mesitylene (20.0 µL) as an internal standard, and a stir bar. The flask was then transferred outside and connected to a Swagelok system equipped with a vacuum pump and gas valves. The argon atmosphere in the flask and argon dissolved in the solvent was removed by three freeze-pump-thaw cycles. The suspension was ultrasonicated (15 minutes). Subsequently, N_2O (2.0 bar, 11.8 mmol w/solvent) was introduced at room temperature. This was followed by the introduction of dihydrogen (2.0 bar, 10.9 mmol) via overpressure using a dosing valve, resulting in a final gauge pressure of 3.0 bar (4.0 bar absolute). The reaction mixture was stirred at 80 °C for 23 hours (THF) and 38 hours (water). For gas sampling, the Schlenk flask was connected to the Swagelok system, which was equipped with a GC-septum and a pressure gauge. While the Schlenk flask was closed, the inner volume of the tubing was evacuated. Valve B was then closed, and the Schlenk flask was opened. Using a pressure-tight syringe, three aliquots were taken and analyzed using GC-TCD to determine the N_2 content. Restarting a catalytic run was done by following the protocol from and including the freeze-pump-thaw step. Finally, in the case of THF, the Schlenk flask was opened under an argon atmosphere, and 0.5 mL of the solution was quickly transferred into a dry NMR tube for ^{1}H NMR analysis of water content.

14.3 Supplementary notes of the general procedure of the N_2O hydrogenation reaction in heterogeneous solid-gas phase

Following the previously described protocol (11.2), with slight modifications, catalyst **NHC-POP-[Rh-N]** (20.0 mg, 2.4–3.4 wt% [Rh], 0.0000.00466–0.00661 mmol [Rh], 430–610 ppm [Rh]) was placed in the bottom of the reactor and water (25.0 µL) was added to the powder under inert conditions. The argon atmosphere in the flask was removed by three pump-thaw cycles, and the reaction was initiated as before pressurized with N_2O and H_2 (1.25 bar, 6.8 mmol each), and allowed to react for 96 hours at 80 °C.

14.4 Supplementary notes of the general procedure for alcohol dehydrogenation reaction using N_2O as hydrogen acceptor

In an argon filled glovebox, an ace pressure tube (35mL, #15) equipped with an adapter (#15 Ace-Thred™ to 1/4in FNPT, 3/8in drill-thru, PTFE) and a CAPFE O-ring (size 110), was charged with **NHC-POP-[Rh-NH]** [1.7 mg, 2.4–3.4 wt%, 0.48 (± 17%)[a] µmol, 300 (± 17%)[a] ppm], *t*BuOM [1.6 mmol, M = K (BzOH), Na (MeOH, EtOH)], alcohol RCH_2OH [1.0 mL, 9.65 mmol (R = Ph), 17.15 mmol (R = Me), 24.7 (R = H)], THF (5.0 mL), and a stir bar. The argon in the atmosphere of the flask and argon dissolved in the solvent was removed by three freeze-pump-thaw cycles. The suspension was ultrasonicated (15 minutes). Subsequently, N_2O (3.0 bar, 19.1 mmol w/solvent) was introduced at room temperature, resulting in a final gauge pressure of 2.0 bar (3.0 bar absolute). The reaction mixture was stirred at 100 °C for 115 hours. For gas sampling, the flask was connected to the Swagelok system, which was equipped with a GC-septum and a pressure gauge. While the flask was closed, the inner volume of the tubing was evacuated. Valve B was then closed, and the flask was opened. The gas was sampled using a pressure-tight syringe, three aliquots were taken and analyzed with GC-TCD to determine the N_2, H_2, content and detection of N_2O and CO_2. Finally, the flask was opened under an argon atmosphere and the liquid evaporated by reduced pressure, then the solid content of the flask was dissolved in D_2O (1.0 mL). Internal standard (Na_3citrate) was added to the solution and 0.5 mL of the solution was quickly transferred into a dry NMR tube for ^1H NMR analysis of carboxylate content.

[a]: The standard deviation of 17% is based of the amount of Rh content on the polymer calculated over multiple synthetic batches.

14.5 Supplementary tables and figures of catalysis screening

Table S4. Screening of catalysts and control experiments for N_2O Hydrogenation

Catalyst	TON_{N2}[a] (TON_{H2O})[b]	$TTON_{N2}$[a] ($TTON_{H2O}$)[b]	TOF_{N2} (TOF_{H2O}) [h^{-1}]
**NHC-POP-[Rh-NH]** [c]	-	-	-
**NHC-POP-[Rh-N]** [c]	40’900 (-)	199’300 (405’600)	1’780 (3’527)
**Complex 3** [d]	-	-	-
**Complex 3** [e]	-	-	-
**Complex (B-CH)** [d]	-	-	-
**Complex (B-CH)** [e]	-	-	-
**Complex (B-CO2)** [d]	-	-	-
**Complex B** [e]	16’420 (15’250)	-	171 (171)
**Complex B2** [d]	19’200 (18’520)	-	200 (193)
**Complex A** [e]	24’900 (24’050) [g]	-	260 (250)
**Complex A** [f]	187’390 (235’000) [g]	-	1’115 (1’370)

[a] TON based on N_2 quantified by GC-TCD. [b] TON based on H_2O quantified by ^1H-NMR using mesitylene as internal standard. Experimental conditions: [c] catalyst (1.0 mg, 2.4–3.4 wt% [Rh], 0.23-0.33 µmol [Rh], 20–30 ppm [Rh]), N_2O (2.0 bar, 11.8 mmol w/solvent), H_2 (2.0 bar, 10.9 mmol), THF (5.0 mL), mesitylene (20.0 µL), reactor volume of 135 mL, temperature of 80 °C, and reaction time of 23 hours. [d]: catalyst (0.33 µmol, 0.13 mM, 0.003 mol%, 30 ppm), temperature of 65 °C, reaction time of 96 hours. [e]: same as in [d] but with base *t*BuOK (3.3 µmol, 1.3 mM, 0.030 mol%). [f]: catalyst (0.033 µmol, 0.013 mM, 0.0003 mol%), base (0.33 µmol, 0.13 mM, 0.003 mol%). [g]: reaction was completed as no H_2 was detected by GC-TCD.

Table S5. Control experiments of N_2O hydrogenation using a high catalyst loading

Catalyst	Solvent	N_2 [mmol]	Yield N_2 [%] [a]	TON [a]	TOF [h^{-1}]
**NHC-POP-[Rh-N]** [b]	THF	2.56	90.8	187 (± 32)	9
**NHC-POP-[Rh-N]** [c]	THF	0.81	28.0	59 (± 10)	3
**NHC-POP-[Rh-N]** [d]	THF	-	-	-	-
**NHC-POP-[Rh-N]** [e]	THF	1.67	94.8	305 (± 52)	5
**NHC-POP-[Rh-N]** [e]	H_2O	1.33	75.4	242 (± 42)	4
**NHC-POP-[Rh-N]** [f]	-	6.54	96.6	1’195 (± 206)	18

[a]: From N_2 quantification by GC-TCD. Reaction conditions [b]: catalyst (50.0 mg, 4135–5858 ppm [Rh]), N_2O (2.0 bar, 3.68 mmol [w/solvent]), H_2 (2.0 bar, 2.82 mmol), THF (5.0 mL), reactor volume of 35 mL, temperature of 80 °C, and reaction time of 20 hours. [c]: using filtered catalyst from entry 1. [d]: using the filtered solution from filtered catalyst of entry 1. [e]: catalyst (20.0 mg, 2650–3755 ppm [Rh]), N_2O (1.25 bar, 2.30 mmol [w/solvent]), H_2 (1.25 bar, 1.76 mmol), THF (5.0 mL), reactor volume of 35 mL, temperature of 80 °C, and reaction time of 20 hours. [f]: same as in [e] but using a reactor with volume of 135 mL and no solvent.

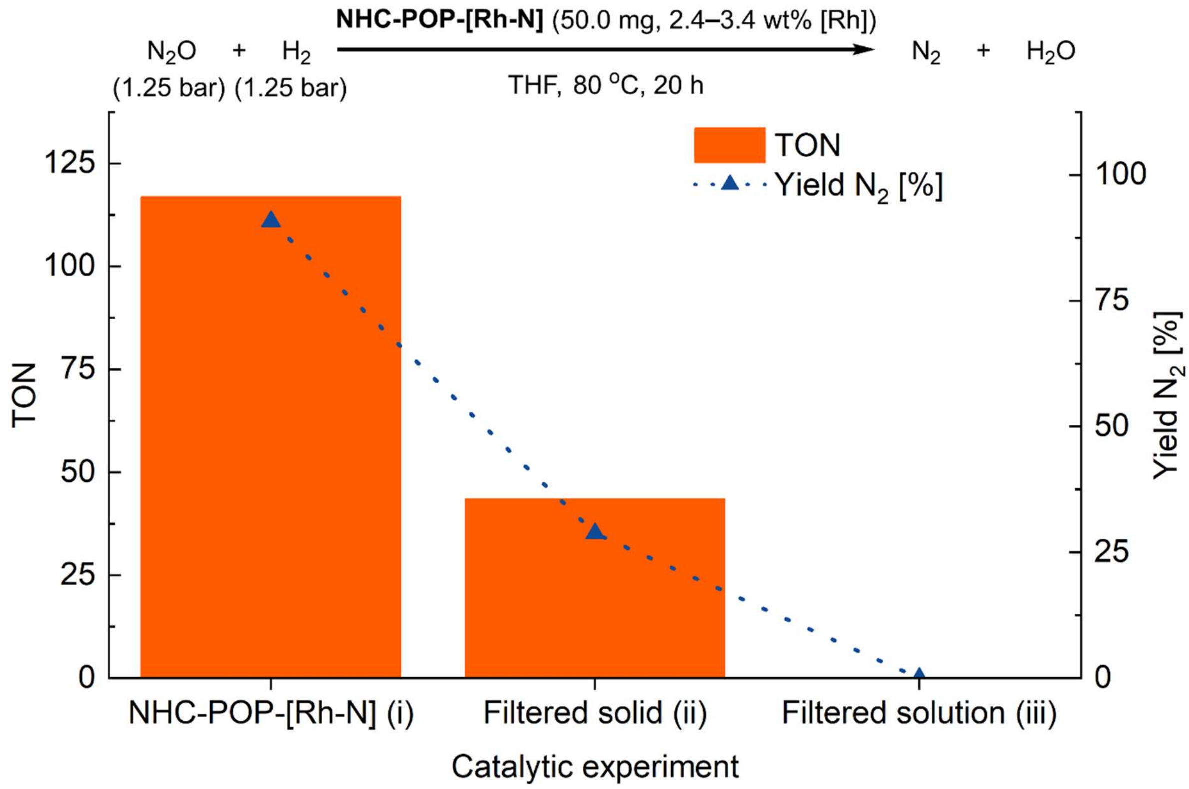

Figure S52. Catalytic control experiments of **NHC-POP-[Rh-N]** at high loading in THF suspension. **i**, First catalytic run stopped after reaching 90% yield N_2. **ii**, Second catalytic run using the filtered solid catalyst. **iii**, Second catalytic run using the filtered solution. Each run was conducted for 20 hours at 80 °C. These experiments demonstrate that approximately two-thirds of **NHC-POP-[Rh-N]** activity is lost under high loading and concentration conditions, while any leached species do not contribute to catalysis.

Table S6. Screening of temperature in N_2O hydrogenation using catalyst **B** [a]

**Entry**	**Temperature (°C)**	**Time** [h]	**TON**[b]	**TOF** [h^{-1}]
**1**	r.t.	48	285	6
**2**	50	48	1’915	40
**3**	80	22	2’809	128

[a] Reaction conditions: N_2O (17.0 mmol), H_2 (10.9 mmol), catalyst **B** (7.0 µmol) and *t*BuOK (70.0 µmol) THF (5.0 mL). [b] Based on H_2O quantified by ^{1}H-NMR using mesitylene as internal standard. The outcome of three catalytic experiments using complex **B** with *t*BuOK at different temperatures. The catalyst performance was positively influenced by the increase in temperature (r.t. to 80°C).

Table S7. Catalyst screening in the dehydrogenative coupling of EtOH using N_2O as hydrogen acceptor. (conditions adapted from reference [8])

Entry	Catalyst	Yield [%][d]	TON	TOF [h^{-1}]
1	[$Ru_2H(\mu\text{-}H)(Me_2dad)(dbcot)_2$]	70	191	11
2[b]	[$RuCl_2(Me_2dae)(dbcot)$]	17	46	3
3[c]	K[$Ru_4(\mu\text{-}H)(Me_2dad)_2(dbcot)_4$]	64	173	10
4	[$Ru_2(NCCH_3)(\mu\text{-}H)(Me_2dad)$ $(dbcot)_2$]PF_6	34	96	5
5[b]	$K(dme)_2$[$RuH(trop_2dad)$]	36	98	5
6[b]	[$Ru(OAc)_2(CO)_2(phen)$]	1	3	<1
7[b]	[$RuCl_2(PPh_3)_3$]	2	5	<1
8[b]	[$Rh(trop_2NH)(PPh_3)$]OTf	16	44	2
9[b]	[$Rh(trop_2NH)(MeIMe)$]OTf (**B**)	41	110	6
10[b]	[$Co(trop_2NH)(PPh_3)$]	–	–	–
11[b]	[$Co(trop_2NH)(MeIMe)$]OTf	–	–	–
12	No catalyst	–	–	–

[a] Conditions: Catalyst (11.5 µmol, 0.4 mol %), KO^{t}Bu (3.1 mmol), THF (0.5 mL), EtOH (0.5 mL), N_2O (11.1 mmol, 1.4 bar) at 65 °C for 18 h. [b] 0.8 mol % catalyst. [c] 0.2 mol % catalyst. [d] Yield determined by NMR analysis of crude reaction mixture with Na_3cit as standard.

Table S8. Dehydrogenative coupling of primary alcohols with N_2O as hydrogen acceptor.

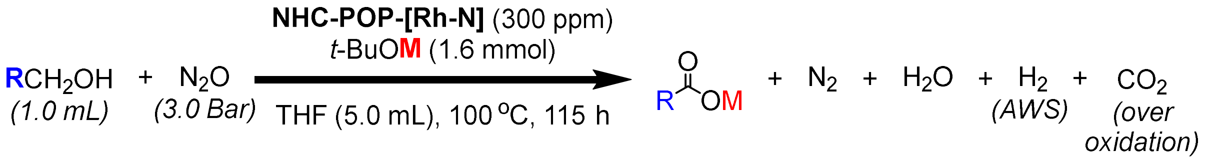

Entry	R	M	[RC(O)O]M [P] (mmol)	N_2 (mmol)	H_2 (mmol)	CO_2 (detected)	Yield[P] (%)	TON[P] {TON[N2]}	TOF[P] {TOF[N2]}
1[a]	Ph	-	-	-	-	-	-	-	-
2	Ph	K	1.51	2.14	0.84	-	94	3140 {4450}	27 {39}
3	H	Na	1.04	3.16	0.47	[b]	65	2160 {6580}	19 {57}
4	Me	Na	0.52	0.72	0.27	-	33	1080 {1500}	9 {13}
5[c]	Ph	K	0.36	0.85	0	-	23	750 {1770}	7 {15}
6[d]	H	-	0.16 [e]	0.62	0.25	[f]	10	23 {89}	0.5 {2}

[a]: Control experiment using **NHC-POP-[Rh-NH]** (non-activated) without base. [b]: CO_2 product detected (ca. 8 times more concentrated than in air). [c]: THF/H_2O (2.5/2.5 mL) was used as solvent mixture. [d]: Catalyst **B** (5.0 mg, 0.007 mmol, 140 ppm), no base, no solvent, MeOH (0.45 mL, neat), 48 hours, H_2O product detected (0.42 mmol), [e] Product is formic acid. [f] CO_2 product detected (ca. 10 times more concentrated than in air).

14.6 Supplementary notes of H_2O quantification using ^1H NMR-spectroscopy

1) Protocol and method:

The amount of H_2O was quantified by ^1H-NMR using mesitylene as internal standard, with NS = 16 and d1 = 10. Aliquots were taken from the reactions (0.5 mL) and transferred into oven dried NMR tubes using standard Schlenk/vacuum line techniques.

2) H_2O calibration/verification of method:

Calibration was done by preparing a stock solution of mesitylene (20 µL, 0.144 mmol) in THF (5.0 mL) in a glovebox. A total of 8 NMR samples with 0.5 mL of the stock solution was prepared in which different amounts of water (degassed) were added using Schlenk techniques. A good fit with the theoretical data was found. The proportion of the variance (R-squared) in the regression was found to be 0.9985, with an estimated overall error of ± 2.0% deviation from theoretical to experimental values. Note that the chemical shift of H_2O varies greatly given the concentrations.

Table S9. Raw data from the water calibration

**Compound**	**Mesitylene**					**H_2O**				
**Entry**		**0**	**1**	**2**	**3**	**4**	**5**	**6**	**7**	**Average**
**Volume in NMR (µL)**	2	1.8	4.5	9.0	18.1	27.1	36.1	72.3	108.4	-
**Chemical Shift (ppm)**	2.59[a]	2.91	3.05	3.17	3.44	3.64	3.79	4.18	4.37	-
**Integral (NMR)**	9[b]	15.4	42	71.1	138.8	211.3	282.4	502.7	814.2	-
**#Molecules (relative)**	1	7.7	21	35.6	69.4	105.7	141.2	251.4	407.1	-
**Amount (mmols) [Exp]**	-	0.11	0.30	0.51	1.00	1.52	2.03	3.61	5.85	-
**Amount (mmols) [Theory]**	0.014	0.10	0.25	0.50	1.00	1.50	2.00	4.00	6.00	-
**Concentration (mM) [Exp]**	-	221	604	1022	1996	3038	4060	7227	11706	-
**Concentration (mM) [Theory]**	28.8	200	500	1000	2000	3000	4000	8000	12000	-
**Error (Theory/Exp)**	-	0.90	0.83	0.98	1.00	0.99	0.99	1.11	1.03	**0.98**

[a] Mesitylene –CH_3 [b] Mesitylene –CH_3 integral was always normalized to 9 protons (one molecule).

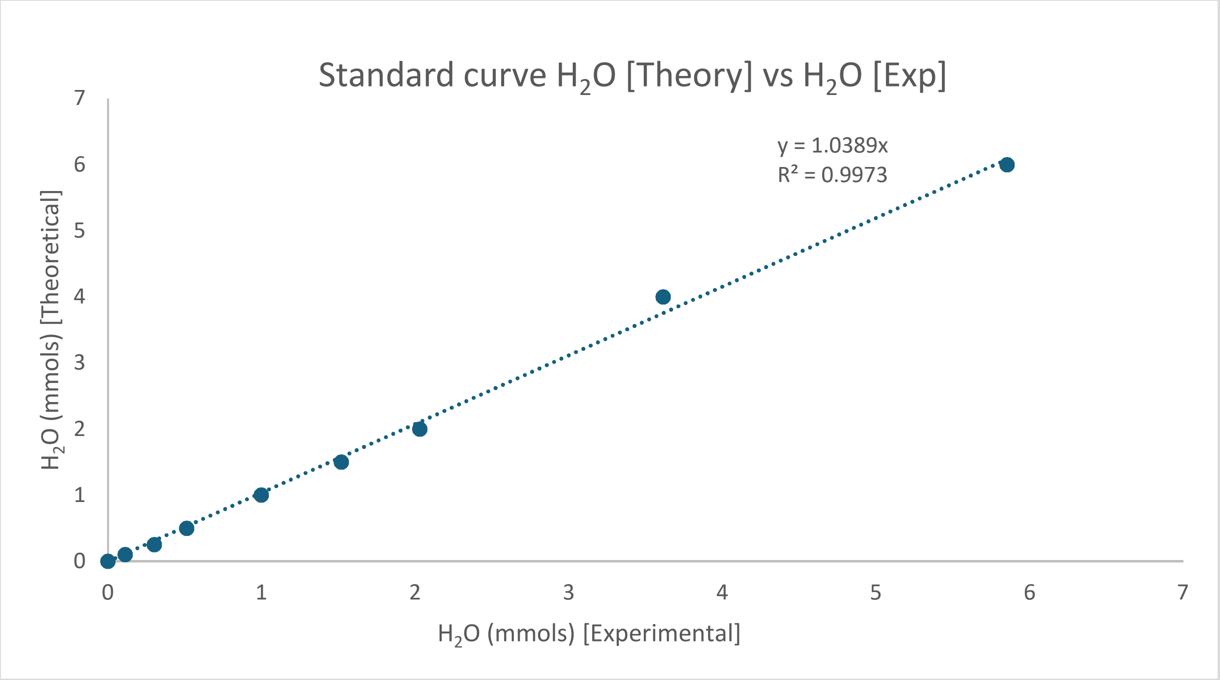

Figure S47. Standard curve of H_2O theoretical versus experimental amount.

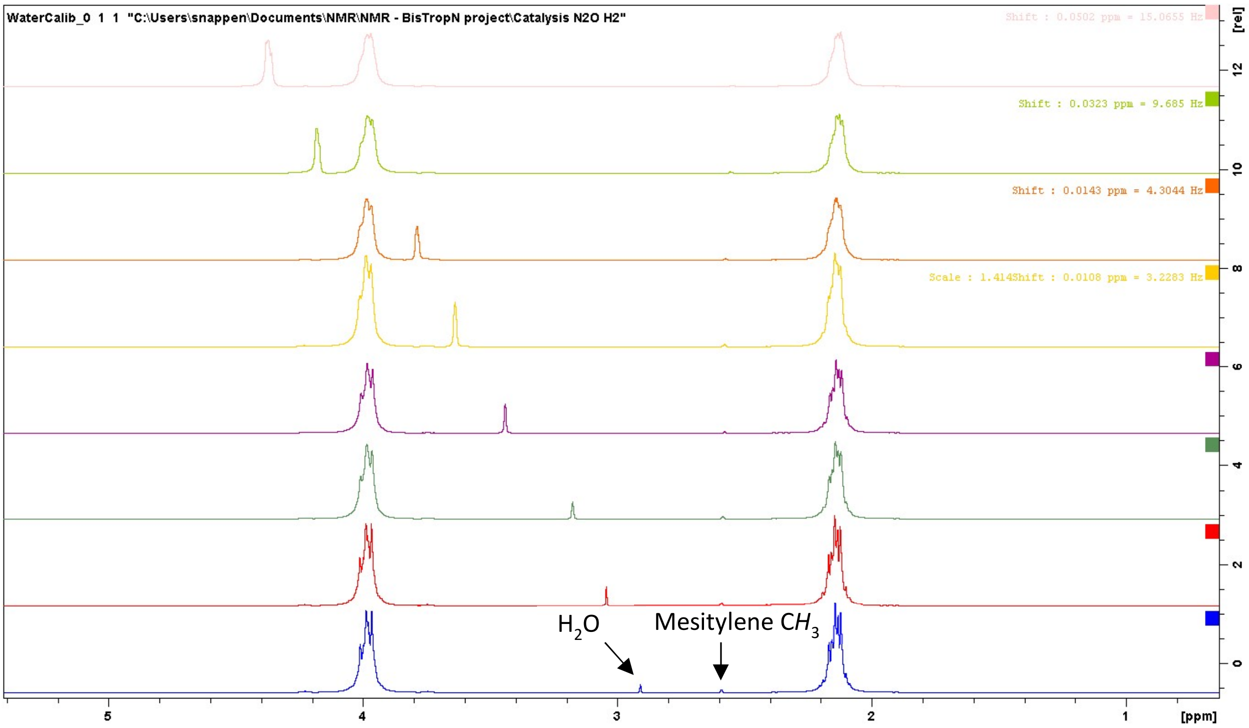

Figure S48. ^{1}H-NMR spectra of H_2O at different concentrations in THF.

3) Blank test:

A blank test was performed by loading a Schlenk flask with THF (5.0 mL) and internal standard (mesitylene, 20 µL) in a glovebox. The flask was then transferred outside and degassed by three freeze-pump-thaw cycles, before N_2O was introduced (through a P_2O_5 drying column) at room temperature. The Schlenk flask was then cooled to −196°C and H_2 was introduced. After 1 hour an aliquot (0.5 mL) of the solution was quickly transferred to a dry NMR tube using Schlenk techniques. An insignificant amount of H_2O was observed (estimated quantity of 0.002 mmol, 0.4 mM), which is less than the catalyst amount used in catalysis (TON < 1).

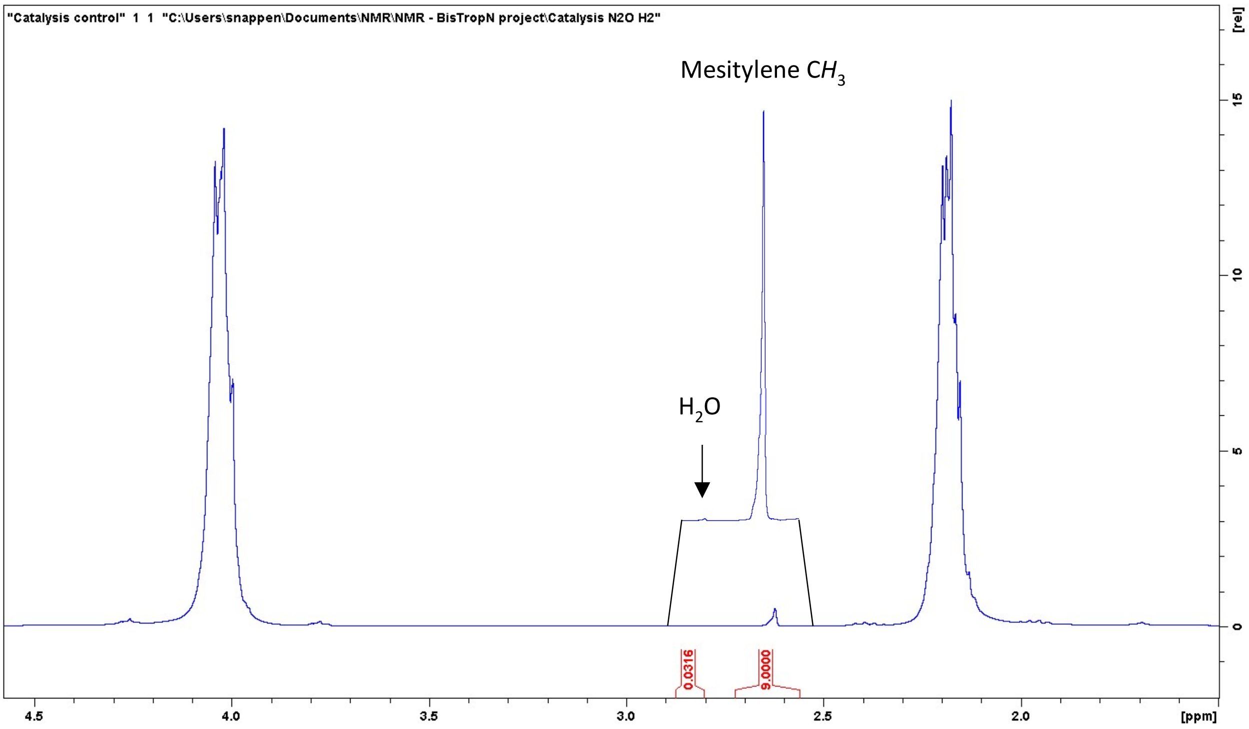

Figure S49. ^{1}H NMR spectrum of H_2O blank test.

14.7 Supplementary notes of the N_2 quantification using GC-TCD

1) GC-TCD Method specifications:

N_2/H_2: (Agilent Technologies 7890A GC system)

- *Oven:* 50 °C; Run time: Until stopped
- *Inlet:* Heater: 150 °C; Split ratio: 50:1; Split flow: 50 mL/min; Gas saver: OFF
- *Detector:* Heater: 180 °C; Reference flow: 20 mL/min; Makeup flow: 5 mL/min

N_2O: (Agilent Technologies 7890A GC system)

- *Oven:* 50 °C for 3 min; Then 40 °C/min to 160 °C for 5.25 min; Run time: 11 min
- *Inlet:* Heater: 150 °C; Split ratio: 50:1; Split flow: 50 mL/min; Gas saver: OFF
- *Detector:* Heater: 180 °C; Reference flow: 20 mL/min; Makeup flow: 5 mL/min

CO_2: (*SRI 8610C Gas Chromatograph)*

- *Oven:* 90 °C
- *Flow*: 50 mL/min
- *Detector*: FID detector equipped with a methanizer

2) Control experiments/blanks:

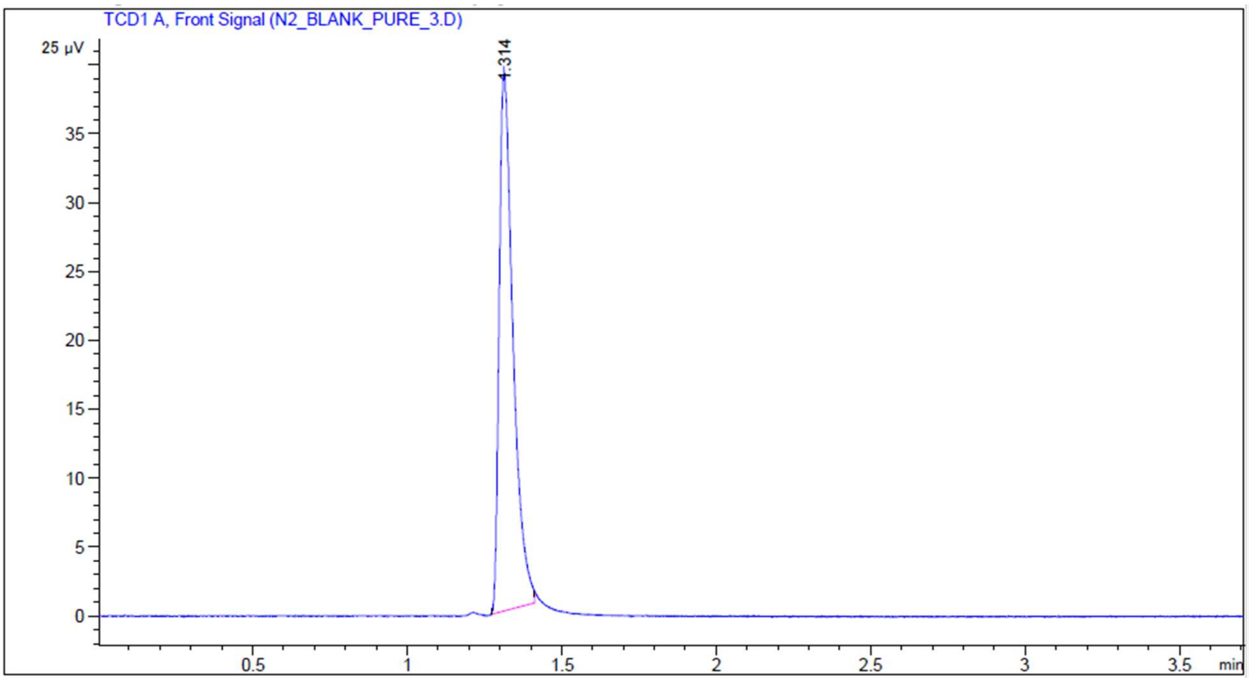

Figure S56. Pure N_2 GC-TCD signal.

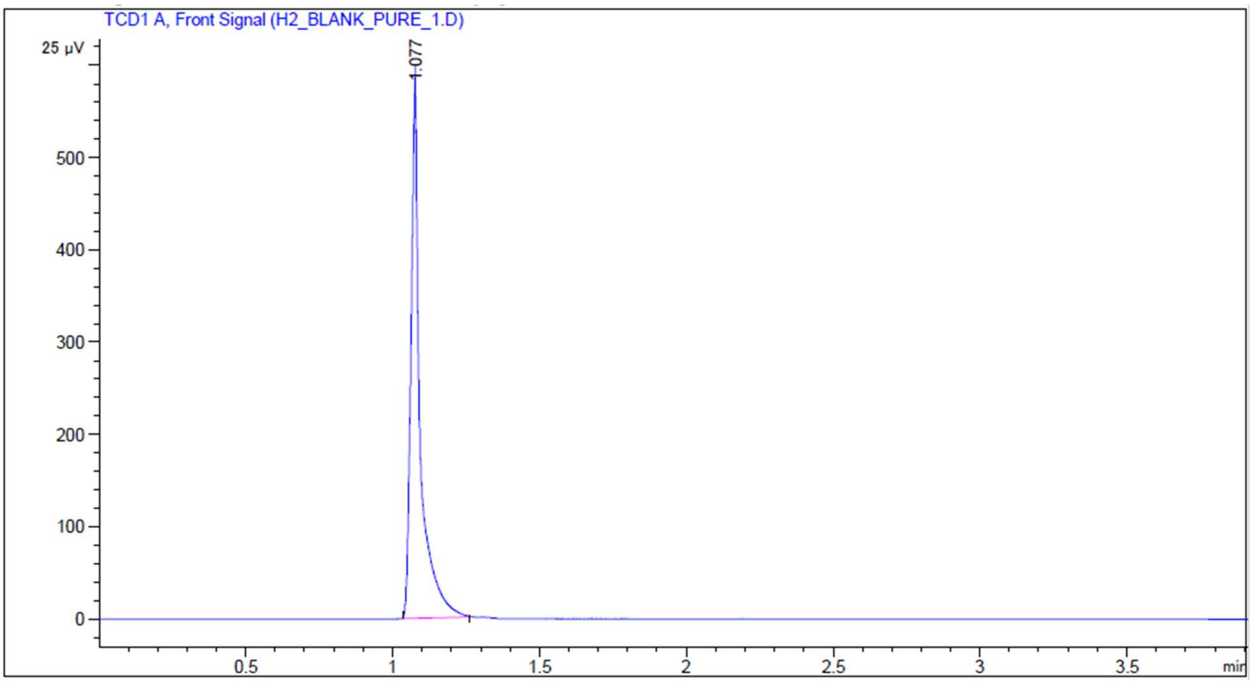

Figure S57. Pure H_2 GC-TCD signal.

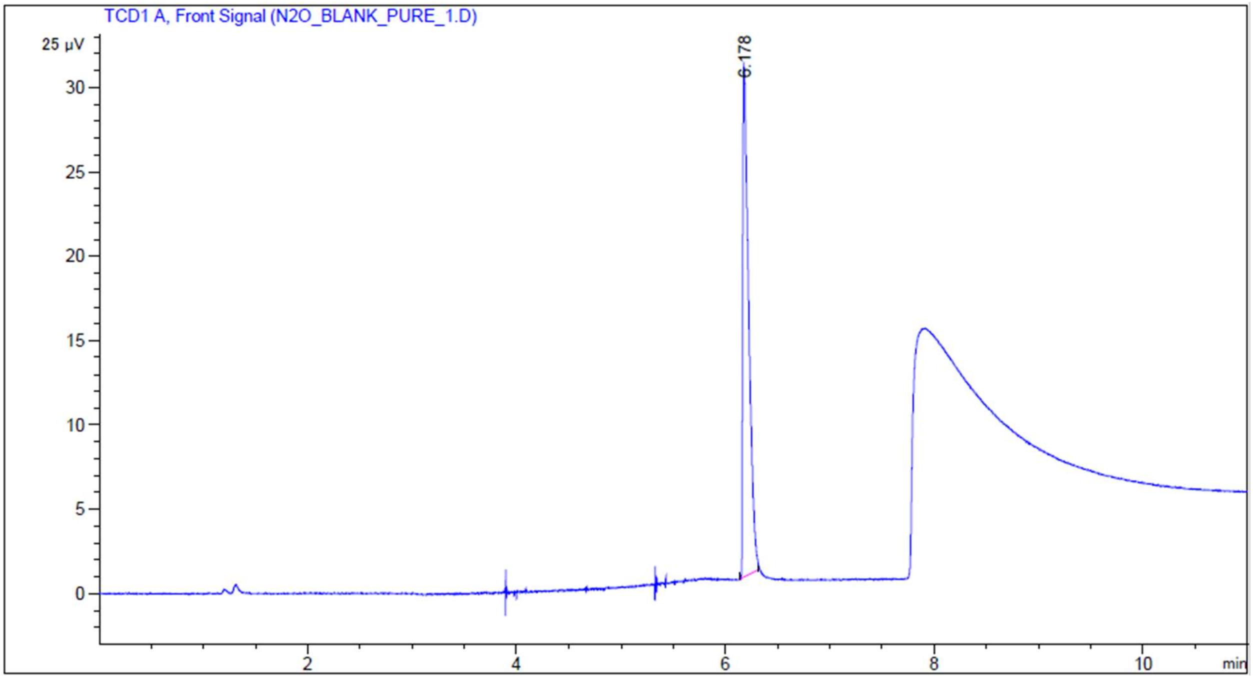

Figure S58. Pure N_2O GC-TCD signal.

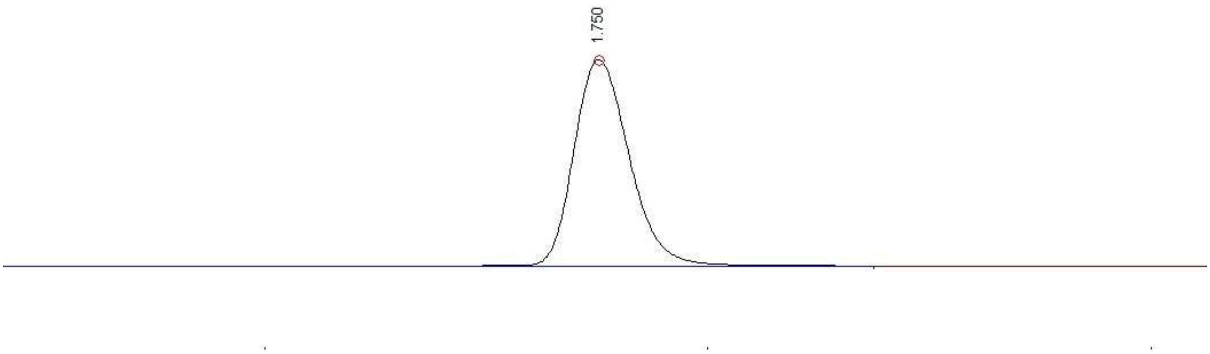

Figure S59. Pure CO_2 GC-TCD signal.

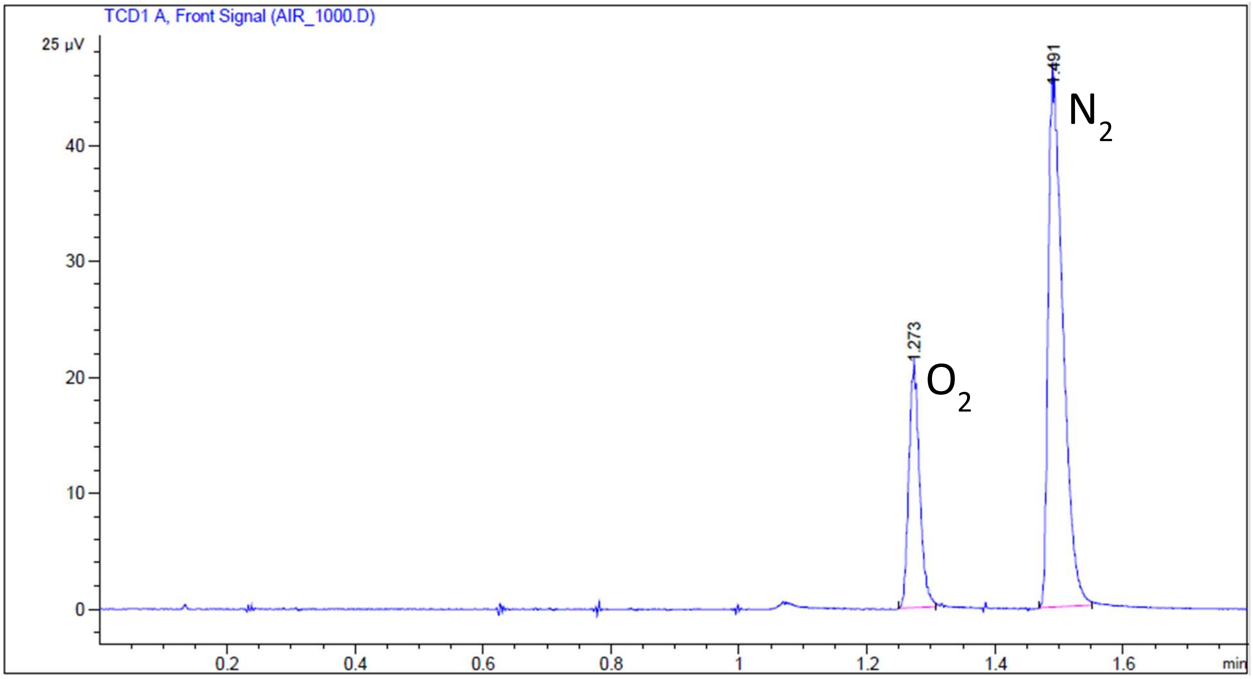

Figure S60. Pure air GC-TCD signal.

3) Pure N_2 calibration and testing/verification of method:

Nitrogen (N_2) was calibrated using a high-vacuum line. The reactor (35 mL) was evacuated (1x10^{-5} bar) and backfilled with an overpressure of N_2 (3.5 bar absolute). With a 500 µL gas tight syringe a total 5 aliquots of each distinct volumes, $V_{Syringe}^{Calib}$ = 100, 80, 60, 40, 20 µL were taken from each single control. The overpressure in the syringe was vented to atmospheric pressure (i.e. 1.0 bar absolute), before the sample was manually injected into the GC-TCD. The averages of the given areas (A_{GC-TCD}^{N2}) were plotted as a function of the normalized N_2 partial pressure (P_{N2}^{norm}). Note, due to normalization, the partial pressure equals the mole fraction of N_2 (x_{N2}) (See VI).

The proportion of the variance (R-squared) in the regression was found to be 0.9985 and the calibration factor (CF) of 8.5909 are used in the interpolation, i.e. when transferring the area (signal) to partial pressure (mbar).

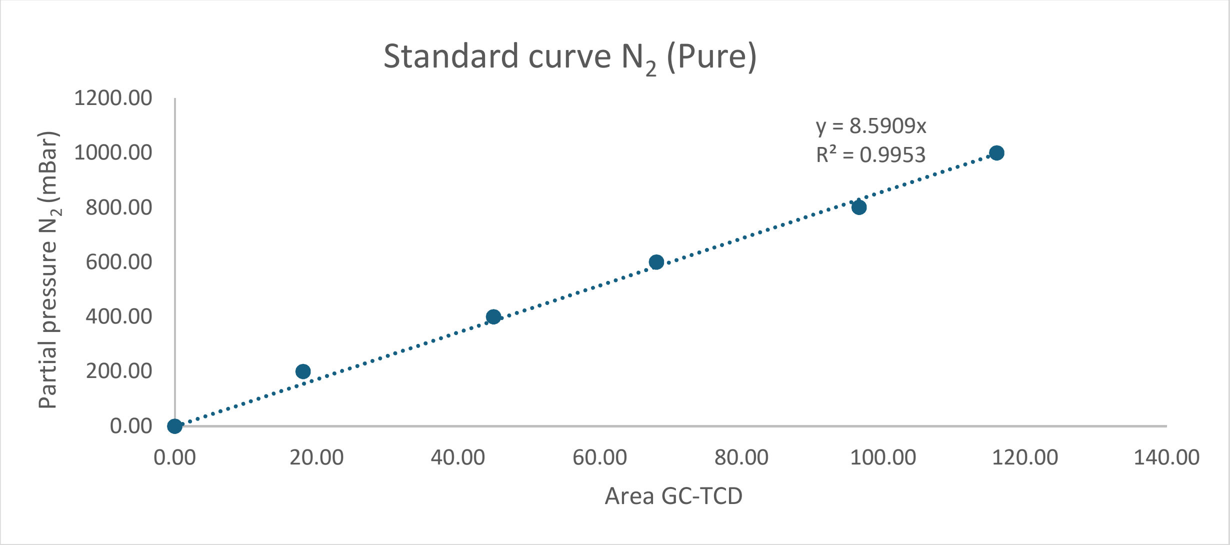

Figure S61. Pure N_2 calibration standard curve.

4) Method control and linearity

As a control of the method, air was also injected in different volumes (as in part III) and plotted alongside the plot of pure N_2. The relative response (Area/P_{N2}) was plotted to check if the signals were within the linear regime of the GC-TCD.

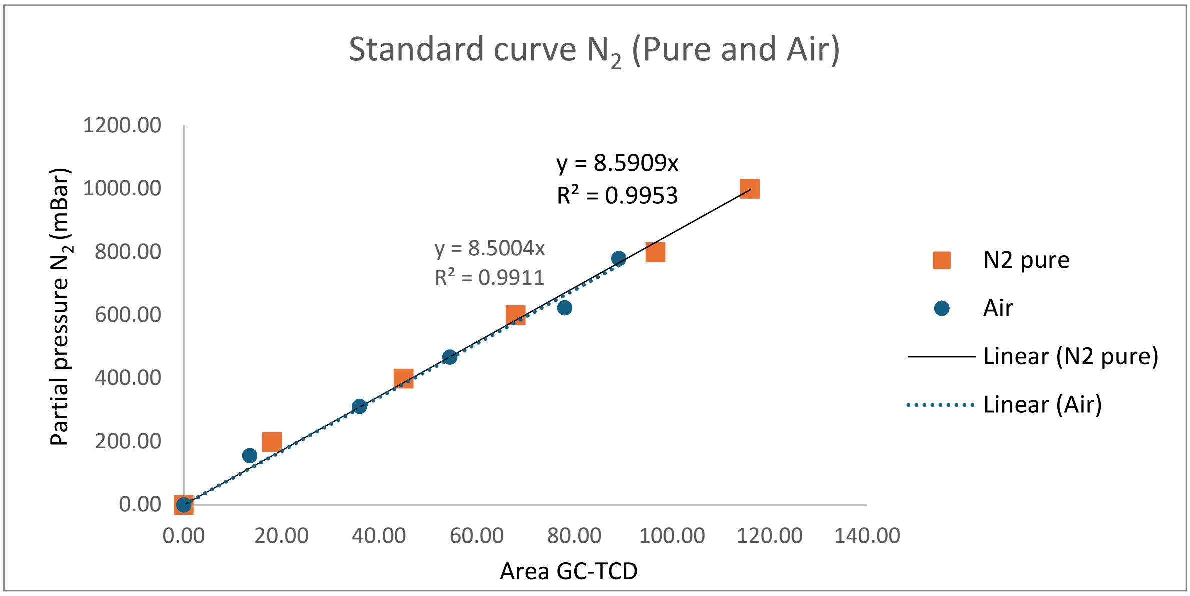

Figure S62. Pure N_2 and air calibration standard curve.

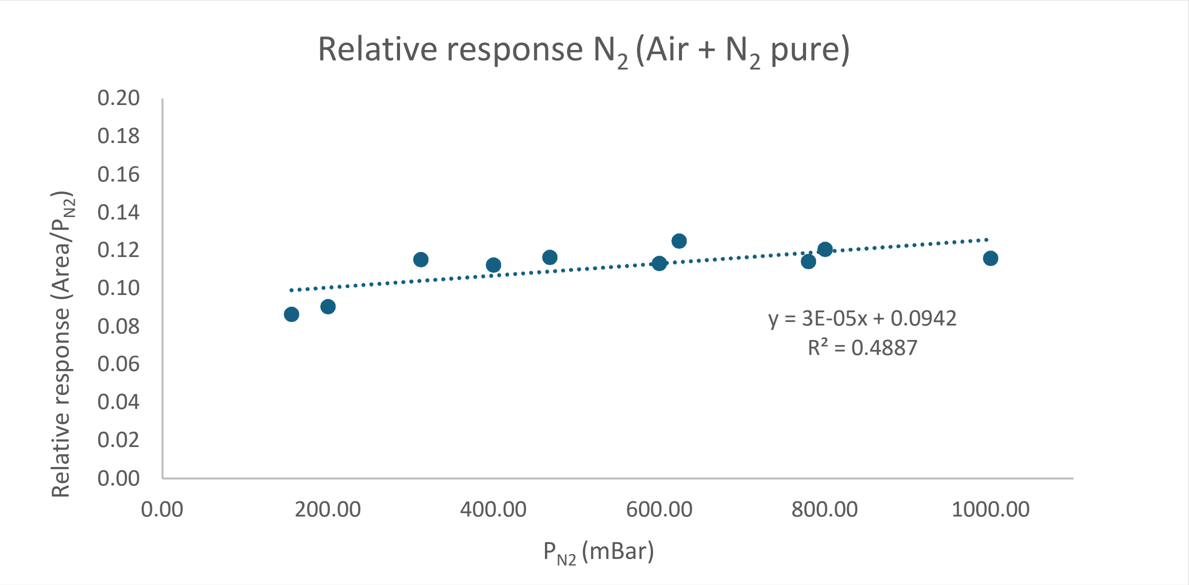

Figure S63. The relative response of N_2.

5) Calibration of N_2 under 'operating conditions':

Nitrogen (N_2) was calibrated in neat N_2 atmosphere under 'operating' conditions. The reactor (35 mL) was loaded with THF (5.0 mL) and degassed by three freeze pump thaw cycles. At room temperature, the reactor was quickly backfilled with an overpressure of N_2 (3.5 bar absolute). The reactor with the THF solution and N_2 atmosphere was then connected to the reaction setup and heated to 65 °C. After 30 minutes, while heated, a total 5 aliquots of each distinct volumes, $V_{Syringe}^{Calib}$ = 100, 80, 60, 40, 20 µL were taken from each single control (like in step III). The overpressure in the syringe was vented to atmospheric pressure (i.e. 1.0 bar absolute), before the sample was manually injected into the GC-TCD. The averages of the given areas (A_{GC-TCD}^{N2}) were plotted as a function of the normalized (vented) N_2 partial pressure (P_{N2}^{norm}). Due to normalization, the partial pressure equals the mole fraction of N_2 (x_{N2}). This calibration is made for sampling 100 µL of headspace and venting overpressure before injection into the GC-TCD during live experiments.

In the "operating" calibration, the proportion of the variance (R-squared) in the regression was found to be 0.98 and the calibration factor (C) of 17.484 was used when interpolating the measured signal (A_{GC-TCD}^{N2}) to partial pressure P_{N2} (mbar).

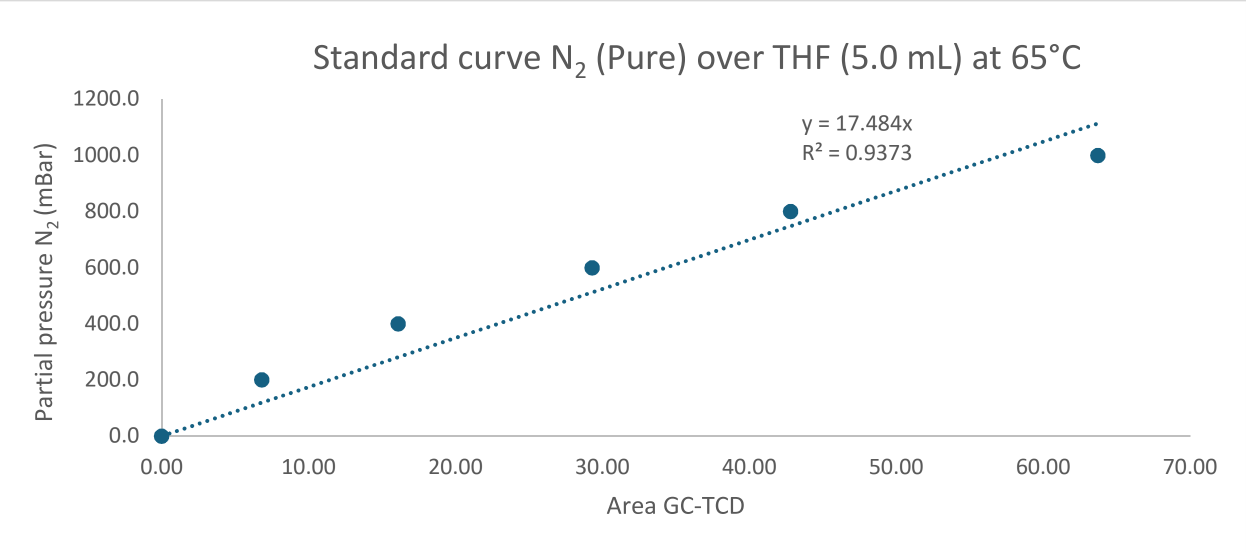

Figure S64. Standard Curve of N_2 under "operating" conditions.

Table S10. Raw data used for the calibration

	N_2					Total error estimation:
**Series**	1	2	3	4	5	**-**
**Syringe Volume (µL)**	100	80	60	40	20	**-**
**Corresponding Normalized Partial Pressure N_2 (mbar)**	**1000**	**800**	**600**	**400**	**200**	**-**
**Total Pressure (mbar) [nominal/real]**	1007	1007	1007	1007	1007	**-**
**Parallel/Area 1**	63.4	43.7	26.1	16.0	7.6	**-**
**Parallel/Area 2**	63.9	39.2	25.7	18.3	6.9	**-**
**Parallel/Area 3**	65.0	39.9	31.3	17.0	6.7	**-**
**Parallel/Area 4**	63.6	44.8	33.8	14.5	6.4	**-**
**Parallel/Area 5**	62.5	46.5	29.8	14.6	6.6	**-**
**Average area**	**63.7**	**42.8**	**29.3**	**16.1**	**6.8**	**-**
**Average deviation**	0.6	2.6	2.8	1.3	0.3	**1.5**
**Average deviation (%)**	1.0	6.1	9.4	7.8	4.8	**5.8**
**Standard deviation**	0.9	3.2	3.5	1.6	0.5	**1.9**
**Standard deviation (%)**	1.4	7.4	11.8	10.1	6.7	**7.5**

6) Quantifying moles N_2:

In the nitrogen quantification two methods can be used: A) GC Injection without normalization and the calculation becomes independent of pressure. B) Normalized GC injection and the calculation is dependent on the pressure monitoring of the reaction. Here, we call normalization when the syringe is vented so that the GC-values are found under or equal to 1 bar of pressure (atmosphere). With method A, we experienced larger errors and less reproducibility in the GC areas (A_{N2}). When the syringe is not vented the volumes are injected with varying amounts and with varying pressures (injection rates), and the areas of the GC signals becomes less reproducible. In method B, the syringe was vented, in other words the GC injection volume (or total moles) was normalized. This method ensured that the total molar amount of gas injected into the GC-TCD give rise to GC signals are within the calibrated area and, importantly, that both the injection volume and rate remains constant. However, the pressure must be monitored to back-calculate from the normalized pressure to the actual pressure and, thus, the amount of moles in the reactor system. In any case, monitoring the pressure was additionally done to ensure that the system was leak tight and it was a valuable experimental parameter that was qualitatively used to monitor the reaction in time as the pressure decreases over the course of the experiment. Method B was ultimately chosen.

During the experiment sampling the found GC-TCD area at time t (A_{N2}^{t}) is transformed into the partial pressure of N_2 (P_{N2}) using the calibration constant (C) by linear interpolation:

$$P_{N2}^{t} = C_{N2} * A_{N2}^{t}\ mbar \xrightarrow{norm.} P_{N2\,norm}^{t} = C_{N2} * A_{N2\,norm}^{t}\ mbar \qquad (1)$$

In the mixture of ideal gases, the mole fraction of nitrogen can be expressed as the ratio of the partial pressure nitrogen to total pressure of the mixture. By venting the pressure inside the syringe (injected into the GC-TCD) the partial pressure is normalized to atmospheric pressures, so that the partial pressure of nitrogen at time t (P_{N2}^{t}) becomes equal to the mole fraction of N_2 at time t (x_{N2}^{t}), by definition:

$$x_{N2}^{t} = \frac{P_{N2}^{t}}{P_{total}^{t}} \xrightarrow{norm.} x_{N2}^{t} = \frac{P_{N2}^{t}(vented)}{P_{atmosphere}^{t}} = \frac{P_{N2}^{t}(vented)}{1000\,mbar} = P_{N2\,norm}^{t} \qquad (2)$$

Then, the moles of N_2 at time t (n_{N2}^{t}) can be found by Dalton's law:

$$n_{N2}^{t} = x_{N2}^{t} \cdot n_{Total}^{t} = \frac{P_{N2}^{t}}{P_{total}^{t}} \cdot n_{Total}^{t} \xrightarrow{norm.} n_{N2}^{t} = P_{N2\,norm}^{t} \cdot n_{Total}^{t} \qquad (3)$$

Where, n_{Total}^{t}, follows the ideal gas law under constant volume and temperature:

$$n_{N2}^{t} = \frac{P_{N2}^{t}}{P_{total}^{t}} \cdot \frac{P_{total}^{t} V_{Total}}{RT} = P_{N2}^{t} \cdot \frac{V_{total}}{RT} \xrightarrow{norm.} n_{N2}^{t} = P_{N2\,norm}^{t} \cdot \frac{P_{total}^{t} V_{Total}}{RT} \qquad (4)$$

Where, V_{total} is the total volume of the reactor, and R is the gas constant and T is temperature, and ($\frac{V_{total}}{RT}$) is equal to a constant K under constant T and V.

We can then insert (1) into (4):

$$n_{N2}^{t} = C_{N2} \cdot A_{N2}^{t} \cdot K\ mmol \xrightarrow{norm.} n_{N2}^{t} = C_{N2} \cdot A_{N2\,norm}^{t} \cdot \frac{P_{total}^{t} V_{Total}}{RT}\ mmol \qquad (5)$$

7) Comment/evaluation of method:

The method includes calibration of the complete regime that is used in every catalytic experiment, the syringe pressure is normalized to atmospheric pressure, such that the measured data (area values) are always generated inside the calibrated regime (Interpolation). Due to interpolation, the largest amount of error of moles is related to the deviations found in the calibration and in the gas theory assumptions (ideal gas law/Dalton's law/Henry's law).

14.8 Supplementary notes of the H_2 quantification using GC-TCD

H_2 was calibrated and quantified following the same protocol as in the N_2 quantification (**12.7**).

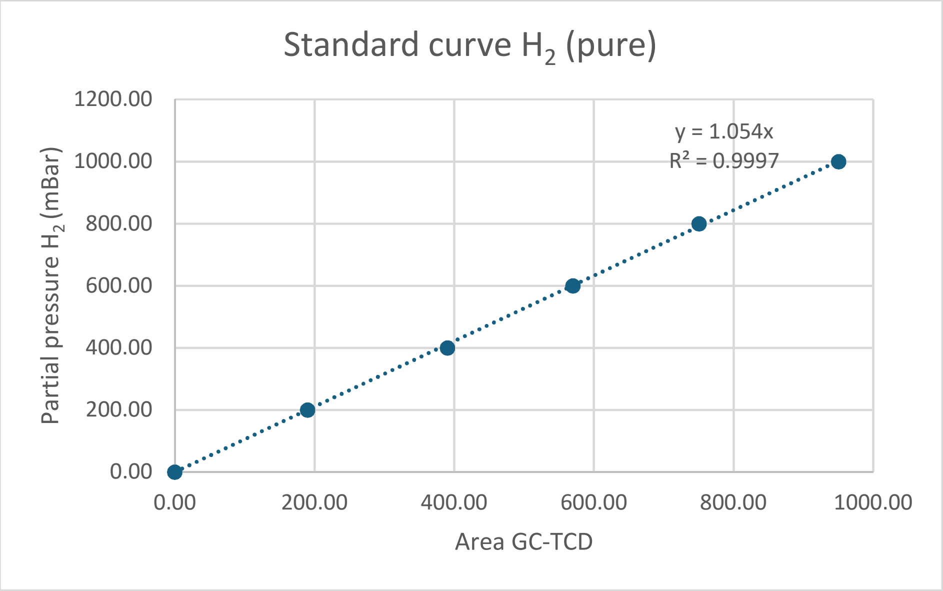

Figure S65. Pure N_2 calibration standard curve.

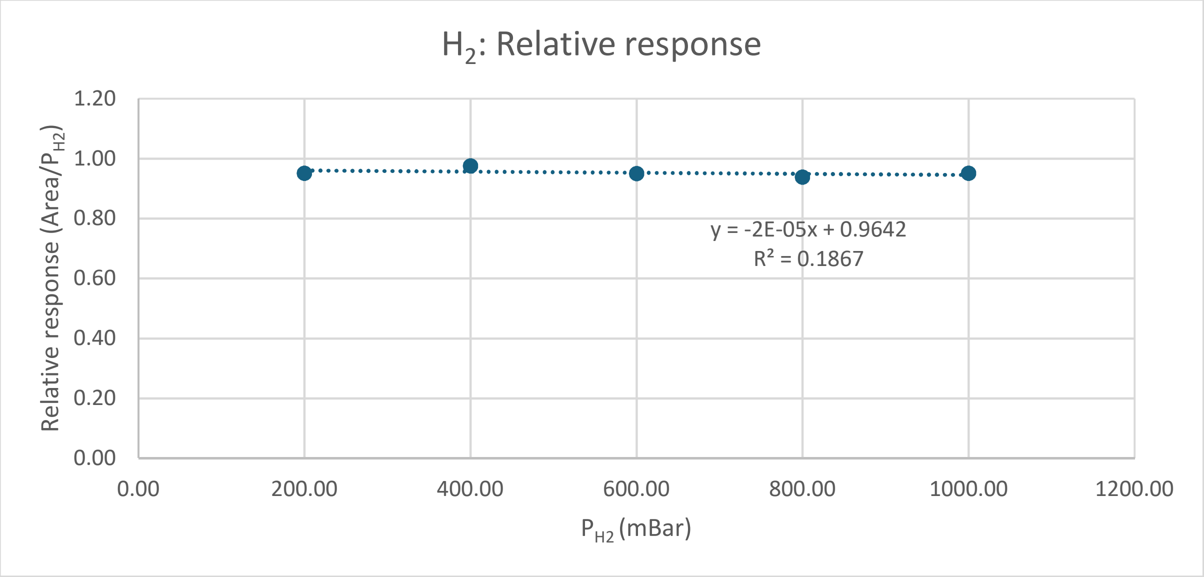

Figure S66. The relative response of N_2.

Table S11. Raw data used for the calibration of H_2.

Average H_2 (Area)	H_2 (Theoretical [mbar])	Relative response (area/mbar)
950.50	1000.00	0.95
750.50	800.00	0.94
570.10	600.00	0.95
390.20	400.00	0.98
190.10	200.00	0.95

15. Supplementary notes of the catalyst robustness

Table S12. Summary of catalyst robustness tests for N_2O hydrogenation

#	TEST	Comments
**1**	STEM analysis post-catalysis with catalyst **A2** or **NHC-POP-[Rh-N]**	High activity, no NPs found
**2**	Catalysis using precursor complex **3**	Decomposition to Rh metal particles, but inactive
**3**	Catalysis using non-activated catalyst **NHC-POP-[Rh-NH]** with no base	NO activity
**4**	Polymerization/hydrogenation of arenes test	NO hydrogenation/polymerization of C_6D_6 or toluene
**5**	Visuals	NO visual metal decomposition
**6**	Restart/recyclability test	Activity retained in diluted conditions over at least five cycles (reproduced in total x 4)
**7**	XPS of **NHC-POP-[Rh-N]** post-catalysis	OK – similar to pre-catalysis state
**8**	**Filtration test**	Catalyst was filtrated, the filtrate was inactive, the washed polymer remained active but some activity was lost

These tests were inspired by a number of reviews on the confirmation of homogeneous VS heterogenous catalysis using metal-organic complexes in solution.[9-12]

1) STEM analysis post-catalysis: The catalytic reaction mixture of **3e** after reaching completion (>99%) was analyzed to discard the formation of Rh nanoparticles with scanning transmission electron microscopy (STEM).

For the STEM investigation, a few drops of the reaction mixture solution were deposited onto a perforated carbon foil supported on a copper grid. After evaporation of the solvent in air, the grid was mounted on the single tilt holder of the microscope. STEM investigations were performed on an aberration-corrected, dedicated STEM microscope, a HD-2700CS (Hitachi). The microscope was operated at an acceleration potential of 200 kV (cold field emitter). A probe corrector (CEOS) that is incorporated in the microscope column between the condenser lens and the probe-forming objective lens provides a resolution below 0.1 nm. Images were recorded with a high-angle annular dark field [(HA)ADF] detector. In HAADF-STEM, the image is generated with incoherently scattered electrons resulting in an intensity strongly increasing with the atomic number (Z-contrast). The images (1024 × 1024 pixels) were recorded with a frame time of 15 s.

The precursor complex (**1** or **2a**), without a stabilizing carbene decomposes to form Rh particles under the catalytic conditions, but these do not show catalytic activity in hydrogenation of N_2O under the applied conditions. Thus, particle formation by itself does not correlate with catalytic turnover. TEM analysis of the complex **3e** post-catalysis showed a uniform distribution of rhodium atoms on the carbon support.

2) Precatalyst test / Decomposition-Activity Decoupling: Under the reaction conditions, the precursor (**3**) degrades into detectable Rh particles, but we do not see catalytic turnover in those "spent" samples. This strongly suggests that the observed particles are an off-cycle or inactive species rather than the main catalytic site.

3) Polymerization/hydrogenation test: We observe **no hydrogenation of arenes** (benzene and toluene) **nor polymerizations** that are frequently observed on reaction pathways involving nanoparticles (especially under forcing or reductive conditions). We would like to point out that in each experiment toluene was present within the reaction mixture because it was used as an internal standard. Reduction or hydrogenation of toluene was never observed. This absence of typical "extraneous" reactivity further supports the assumption that the hydrogenation of N_2O is not heterogeneously catalyzed on a metal surface.

5) Visual observations: When the NHC-bound catalyst (**NHC-POP-[Rh-N]**) is used in large amounts the reaction solutions remain clear, with **no darkening or precipitation**. When metallic particles form in sufficient concentrations, one typically observes visible turbidity or color changes indicative of colloids or precipitants. This is observed when the molecular precursor **3** is used, which would be the leached specie by decoordination from the polymer NHC-site, however, this specie is inactive.

6) Restart/recyclability test: We have performed recycling experiments in which additional substrate is introduced to a finished reaction and the conversions were highly retained (93–100 %) **without significant change in activity**. This observation supports the idea that the Rh–N molecular specie remains active rather than a stable metallic particle phase.

7) Post-catalysis analysis: XPS analysis do not reveal any change in the state of the catalyst (rhodium, nitrogen, carbon). ICP-OES, also reveals minimal metal leaching after use (maximum found 7 % leaching).

8) Filtration test: After catalytic use, the polymer (50 mg) was filtered and washed thoroughly with THF (30 mL). The combined filtrate and wash solution displayed no catalytic activity, indicating that soluble rhodium nanoparticles were not responsible for catalysis. Subsequently, the polymer was transferred to a new reaction vessel and retested. Although some loss in activity was observed, significant catalytic activity remained. These results strongly support the presence of active immobilized rhodium sites. This conclusion is further reinforced by the absence of detectable nanoparticles on the polymer and XPS spectra consistent with those of the original precatalyst.

16. References

1 Maire, P., Büttner, T., Breher, F., Le Floch, P. & Grützmacher, H. Heterolytic Splitting of Hydrogen with Rhodium(I) Amides. *Angewandte Chemie International Edition* 44, 6318-6323 (**2005**).

2 Büttner, T., Breher, F. & Grützmacher, H. Amine olefin rhodium(i) complexes: pKa and NH bond strength. *Chemical Communications*, 2820-2821 (**2004**).

3 Roberts, G. M., Pierce, P. J. & Woo, L. K. Palladium Complexes with N-Heterocyclic Carbene Ligands As Catalysts for the Alkoxycarbonylation of Olefins. *Organometallics* 32, 2033-2036 (**2013**).

4 Kuhn, N. & Kratz, T. Synthesis of Imidazol-2-ylidenes by Reduction of Imidazole-2(3H)-thiones. *Synthesis* 1993, 561 - 562 (**1993**).

5. S. T. Nappen, J. J. Gamboa-Carballo, E. Tschanen, F. Ricatto, M. D. Wörle, A. Thomas, M. Trincado, H. Grützmacher, *Angew. Chem. Int. Ed.* **2025**, e202502616.

6 Gianetti, T. L. *et al.* Nitrous Oxide as a Hydrogen Acceptor for the Dehydrogenative Coupling of Alcohols. *Angewandte Chemie International Edition* 55, 1854-1858 (**2016**).

7 Huang, N., Wang, P., Addicoat, M. A., Heine, T. & Jiang, D. Ionic Covalent Organic Frameworks: Design of a Charged Interface Aligned on 1D Channel Walls and Its Unusual Electrostatic Functions. *Angewandte Chemie International Edition* 56, 4982-4986 (**2017**).

8 Bösken, J., Rodríguez-Lugo, R. E., Nappen, S., Trincado, M. & Grützmacher, H. Reduction of Nitrous Oxide by Light Alcohols Catalysed by a Low-Valent Ruthenium Diazadiene Complex. *Chemistry – A European Journal* 29, e202203632 (**2023**).

9 Whitesides, G. M. *et al.* Suppression of unwanted heterogeneous platinum(0)-catalyzed reactions by poisoning with mercury(0) in systems involving competing homogeneous reactions of soluble organoplatinum compounds: thermal decomposition of bis(triethylphosphine)-3,3,4,4-tetramethylplatinacyclopentane. *Organometallics* 4, 1819-1830 (**1985**).

10 Widegren, J. A. & Finke, R. G. A review of the problem of distinguishing true homogeneous catalysis from soluble or other metal-particle heterogeneous catalysis under reducing conditions. *Journal of Molecular Catalysis A: Chemical* 198, 317-341 (**2003**).

11 Crabtree, R. H. Resolving Heterogeneity Problems and Impurity Artifacts in Operationally Homogeneous Transition Metal Catalysts. *Chemical Reviews* 112, 1536-1554 (**2012**).

12 Chernyshev, V. M. *et al.* Pd and Pt Catalyst Poisoning in the Study of Reaction Mechanisms: What Does the Mercury Test Mean for Catalysis? *ACS Catalysis* 9, 2984-2995 (**2019**).
